# A wearable motion capture suit and machine learning predict disease progression in Friedreich’s ataxia

**DOI:** 10.1038/s41591-022-02159-6

**Published:** 2023-01-19

**Authors:** Balasundaram Kadirvelu, Constantinos Gavriel, Sathiji Nageshwaran, Jackson Ping Kei Chan, Suran Nethisinghe, Stavros Athanasopoulos, Valeria Ricotti, Thomas Voit, Paola Giunti, Richard Festenstein, A. Aldo Faisal

**Affiliations:** 1 Brain & Behaviour Lab, Department of Bioengineering, Imperial College London, London, UK; 2 Brain & Behaviour Lab, Department of Computing, Imperial College London, London, UK; 3 Epigenetic Mechanisms and Disease Group, Department of Brain Sciences, Imperial College London, London, UK; 4 NIHR Great Ormond Street Hospital Biomedical Research Centre, UCL Great Ormond Street Institute of Child Health, London, UK; 5 Great Ormond Street Hospital for Children, NHS Foundation Trust, London, UK; 6 Institute of Neurology, UCL, National Hospital for Neurology and Neurosurgery (UCLH), London, UK; 7 MRC London Institute of Medical Sciences, London, UK; 8 Behaviour Analytics Lab, Data Science Institute, Imperial College London, London, UK; 9 Brain & Behaviour Lab, Institute for Artificial and Human Intelligence, University of Bayreuth, Bayreuth, Germany; 10 Chair in Digital Health, Faculty of Life Sciences, University of Bayreuth, Bayreuth, Germany

**Keywords:** Machine learning, Neurodegenerative diseases

## Abstract

Friedreichʼs ataxia (FA) is caused by a variant of the *Frataxin* (*FXN*) gene, leading to its downregulation and progressively impaired cardiac and neurological function. Current gold-standard clinical scales use simplistic behavioral assessments, which require 18- to 24-month-long trials to determine if therapies are beneficial. Here we captured full-body movement kinematics from patients with wearable sensors, enabling us to define digital behavioral features based on the data from nine FA patients (six females and three males) and nine age- and sex-matched controls, who performed the 8-m walk (8-MW) test and 9-hole peg test (9 HPT). We used machine learning to combine these features to longitudinally predict the clinical scores of the FA patients, and compared these with two standard clinical assessments, Spinocerebellar Ataxia Functional Index (SCAFI) and Scale for the Assessment and Rating of Ataxia (SARA). The digital behavioral features enabled longitudinal predictions of personal SARA and SCAFI scores 9 months into the future and were 1.7 and 4 times more precise than longitudinal predictions using only SARA and SCAFI scores, respectively. Unlike the two clinical scales, the digital behavioral features accurately predicted *FXN* gene expression levels for each FA patient in a cross-sectional manner. Our work demonstrates how data-derived wearable biomarkers can track personal disease trajectories and indicates the potential of such biomarkers for substantially reducing the duration or size of clinical trials testing disease-modifying therapies and for enabling behavioral transcriptomics.

## Abstract (web summary)

Full-body movements captured using a multisensor body suit predict the clinical trajectory of patients with Friedreichʼs ataxia, providing digital biomarkers of disease progression.

## Main

**Fig. 1:**
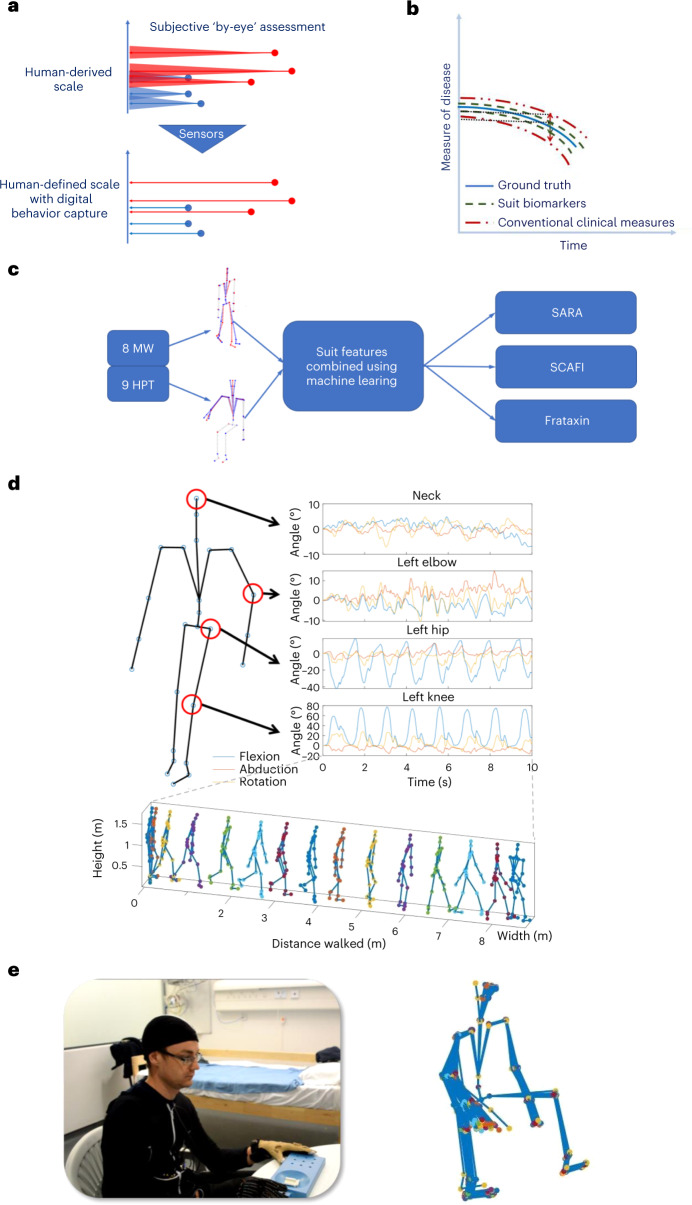
Overview of concepts, methodology and findings. **a**, Current gold-standard clinical assessments for monitoring the effects of neurodegenerative diseases are often measured ‘by-eyeʼ and lack objectivity. Sensors can help achieve accurate and objective monitoring of patientsʼ behavior. **b**, Because of the lack of precision, it can take months before conventional clinical measures reveal any change in disease progression. Motion capture suit biomarkers provide digital biomarkers that can potentially capture subtle changes in patient performance in a shorter time span than conventional clinical measures. **c**, Using a full-body motion capture approach, we analyzed two subassessments SCAFI, the 8 MW and the 9 HPT, for which clinicians use only their duration for estimating FA disease progression. Using a machine learning approach, we generated a series of markers of patient performance with the goal of reconstructing the full SARA and SCAFI scores and predicting *Frataxin* expression levels. **d**, Reconstructed body posture of a participant performing the 8 MW while wearing a motion capture suit, which uses 17 inertial sensors to monitor the movement of the limbs. A typical time series from the angular positions of the neck, elbow, hip and knee joints captured by the suit and a frame sequence of the motion capture data of an FA patient performing the 8-MW test (each frame is 0.5 s apart) is shown. **e**, The 9-HPT setup showing a participant wearing the motion capture suit and a frame sequence of the motion capture data of an FA patient performing the 9 HPT.

About 1 in 17 people suffer from rare diseases, and to date, 6,000 rare diseases have been identified^1,2^. The challenge for drug development and regulatory approval in many rare diseases is their slow progression and small populations. The clinical scales currently used to quantify the progression of neurodegenerative diseases in patients are insensitive to the extremely slow progression of such diseases^3,4^. This means that it might take many months before they reveal any change. Additionally, some of their subcomponents have been shown to lack objectivity^5–7^ as they are mainly based on the subjective estimates collected by clinical personnel, often ‘by eyeʼ. Consequently, there is a pressing need for new methodologies that can achieve accurate and objective monitoring of patientsʼ behavior for neurodegenerative assessments (Fig. 1a). This is most important for monitoring progression in clinical trials that are often excessively long as a result. This is because the length of disease-modifying treatment trials is determined by the time it takes for current gold-standard methods of neurological clinical assessments to detect a slowing or stopping of disease progression (Fig. 1b), sometimes taking years (in the case of Friedreichʼs ataxia (FA) for 2 years) to conclusively establish disease progression.

We use FA as a proof-of-principle model to show how an artificial intelligence (AI)-defined digital biomarker can help to rapidly accelerate and shrink clinical trials for disease-modifying treatments. FA afflicts 1 in 40,000 Caucasian people^8,9^ and is a rare triplet-repeat-expansion neurodegenerative disease with slow progression, frequently leading to an early death by cardiomyopathy by affecting the heart and nervous system. FA predominantly affects the sensory proprioceptive system causing deterioration in patientsʼ coordination that worsens over time. It typically starts during childhood and the main symptoms include poor balance, gradual loss of strength and position-sense in the limbs, spasticity, the curvature of the spine (kyphoscoliosis), impaired speech, hypertrophic cardiomyopathy and it has also been associated with an increased incidence of diabetes, bladder symptoms, impairment of vision and hearing^10,11^.

We are at an exciting time in the research of the underlying disease mechanisms in FA that promises to reveal radical new therapies. FA is caused by homozygosity for a noncoding guanine–adenine–adenine (GAA) triplet repeat expansion within the first intron of the *Frataxin* (*FXN*) gene in about 96% of affected individuals. The other 4% are compound heterozygous for an expansion and another variant in the other *FXN* allele^12^. The expansion, on both alleles of the *FXN* gene, leads to its partial silencing sufficient to cause Frataxin deficiency and hence the disease. Frataxin repression in the dorsal root ganglia and heart is probably the most clinically relevant. Frataxin is essential for normal mitochondrial function and the synthesis of iron–sulfur cluster enzymes, and its deficiency leads to increased susceptibility to oxidative stress. Recent studies by some of us and others have demonstrated that *FXN* gene silencing triggered by the large GAA-repeat expansions involves epigenetic heterochromatinization of the *FXN* gene^13–16^. These findings led to potential disease-modifying therapies aimed at restoring Frataxin expression in patients by using epigenetic modifiers such as histone deacetylase inhibitors^17–19^. Other promising therapies which act downstream of Frataxin deficiency have also been recently trialed^20^ and several large pharmaceutical companies are investing in gene-therapy approaches following recent findings in mouse models^21^. There is currently no cure for FA, despite intensive research and trials of such new therapies. However, a recent trial of omaveloxolone^20^ has shown benefits in FA, and several other promising new treatments are under investigation.

Clinicians observe patients performing various tasks and extract scores, which can semiquantitatively define the stage of the disease. Among the most used tests are the Scale for the Assessment and Rating of Ataxia (SARA)^22^, Spinocerebellar Ataxia Functional Index (SCAFI)^23^ and Friedreichʼs Ataxia Rating Scale (FARS)^4^. All these scales can assess patients’ motor control and coordination skills through a series of evaluations, such as the 8-m walk (8 MW), the 9-hole peg test (9 HPT), the finger-nose test, the finger tapping test and the heel-shin-slide test. Evaluating the overall clinical stage of FA disease requires converting patientsʼ physiological information (for example, stance, walking gait, sitting posture, etc.) into numerical scores, which in some cases (FARS) include patient-reported ability to perform activities of daily living (ADL). Therefore, in many cases, the process of deriving discrete integer scores results in losing information about the disease state and is subjective.

Although these score-based metrics can quantify the ataxia disease, they are still subject to variability due to the subjective estimates of some of their components^6,24^. This variability can be partially reduced with extensive training of the assessors^25^. These metrics also suffer from low accuracy and sensitivity, resulting in extended periods to measure changes in the behavior in FA and the need for much larger numbers of patients in clinical trials: for a 2-year parallel-group trial, 230 patients would be required to detect a 50% reduction in SARA progression at 80% power; 118 if only ambulatory individuals are included. With ADL questionnaire as the primary outcome, 190 patients would be needed, and less patients would be required if only individuals with early onset are included^26,27^.

Here we have applied behavior analytics to capture subtle changes more objectively and accurately and therefore in a shorter time span than the conventional clinical measures. Several motor-behavior-based digital biomarkers for ataxia have been reported in the literature recently^28–32^. While these biomarkers are shown to differentiate between patients and controls, they are gait-based and focused on lower body performance and hence exclude nonambulant patients. In our study, we have taken a more holistic view of the patientsʼ motor capability and have used full-body motion data to define kinematic features that comprised gait and not-gait activities. We used machine learning to evaluate how well these features are predictive of the disease progression and *FXN* levels. We have demonstrated the utility of the digital kinematic features by showing that their predictive power far exceeds that of clinical scales.

## Results

### Overview of approach

Nine FA participants and nine age- and sex-matched healthy controls participated in the study (see Table 1 for the characteristics of the study participants). To enable monitoring of the FA disease progression on a longitudinal timescale, the trial consisted of four clinical measurement points as follows: baseline visit on day-1 visit and follow-up visits on 3 weeks and 3 and 9 months. We used a motion capture suit to record the behavior of FA patients and healthy controls. Participants came into the clinic and performed clinical scales wearing a motion capture suit. We analyzed the movement data and identified kinematic features from the suit data, which were different between the patients and the controls. Then, we established the cross-sectional and longitudinal predictive capacity of the suit features using machine learning and tested the effectiveness of our predictions against the current gold-standard approach using data from a separate and larger EFACTS study^26^ (a two-year longitudinal study with a larger cohort size). Finally, we used the kinematic suit features to regress against *FXN* gene expression—a key FA molecular biomarker.

**Table 1:** Characteristics of the FA participants and healthy controls in our study. Data are presented as mean (range)

	FA participants				Healthy controls
Visit number	Visit 1	Visit 2	Visit 3	Visit 4	Visit 1
*N*	9	9	8	8	9
Female:male	6:3	6:3	5:3	5:3	6:3
Ambulatory:nonambulatory	8:1	8:1	8:0	8:0	9:0
Age (years)	42.54 (24.18–63.32)	42.59 (24.25–63.36)	41.16 (24.50–63.55)	41.80 (24.92–64.16)	44.11 (25.00–66.00)
Disease duration (years)	14.32 (3.93–27.20)	14.37 (3.97–27.24)	13.12 (4.18–27.45)	13.76 (4.85–28.14)	NA
SARA	12.44 (4.00–33.50)	12.22 (5.00–32.50)	10.69 (6.00–16.00)	14.06 (7.50–21.50)	0 (0–0)
SCAFI	−0.74 (−1.69 to 0.16)	−0.67 (−1.63 to 0.08)	−0.60 (−0.96 to 0.16)	−0.70 (−1.07 to −0.09)	1.41 (0.56–2.29)
8 MW (s)	11.46 (6.20–26.90)	10.64 (5.85–18.35)	10.47 (5.45–15.50)	11.69 (5.95–22.45)	5.55 (3.60–8.00)
9 HPT—dominant hand (s)	59.42 (30.10–88.60)	52.28 (33.15–71.35)	49.67 (30.55–84.55)	53.00 (34.00–68.00)	19.98 (15.85–23.95)
9 HPT—nondominant hand (s)	81.07 (34.80–178.20)	60.09 (23.35–103.50)	58.77 (34.20–72.20)	61.55 (39.50–80.85)	23.16 (18.30–28.57)
PATA rate	18.94 (15.50–25.00)	18.56 (13.50–22.00)	18.94 (15.50–23.00)	18.50 (17.00–22.00)	32.56 (25.00–40.00)
*FXN* mRNA levels (in normalized Ct values)	6.06 (4.94–7.06)	6.55 (4.90–8.43)	6.50 (5.44–7.93)	6.27 (5.41–7.36)	–
Age of onset (years)	28.14 (15.00–51.29)				NA
GAA short allele repeats	309 (88–582)				–
GAA long allele repeats	803 (203–1,117)				–

### Kinematic features

To benchmark how FA patientsʼ movement differs from normal, we analyzed the following two subassessments of the SCAFI scale: the 8 MW and the 9 HPT. Currently, clinicians only use the crude measure of duration of these activities for quantifying disease severity^23^. We defined a series of kinematic features of patient performance (Table 2), which can be used to objectively distinguish the differences in the behavior of FA patients from the control population. More specifically, in the 8-MW case, we focused on the behavioral changes of the full-body kinematics whereas for the 9 HPT, we focused on the upper body kinematics because participants were seated during the task (Fig. 1c–e). The latter is important as current scales exhibit ceiling effects^26^, resulting in the exclusion of wheelchair-bound patients from clinical trials. These features have been inspired by the currently used clinical scales, standard gait analysis methods, works on other neurodegenerative diseases with similar movement disorders^33–38^ and direct clinical experience. Several of these features are intuitively meaningful, perhaps even subjectively visible by the eye, while others capture complex and subtle spatiotemporal patterns that may escape even very experienced clinicians.

**Table 2:** Suit features from the 8-MW task (F1–F10) and 9-HPT task (F11–F18) used to train the GP regression algorithm

ID	Feature name	Description	Number of features
F1	Workspace probability density volume and entropy	Volume occupied by the joints calculated using the 3D location of the joints	2
F2	Lower body joint variability	Average variability of the hip and knee joint velocities	2
F3	Walk autocorrelation	PCs of the autocorrelation of the joint angular velocities	2
F4	Channel-delay cross-correlation	Eigen spectrum values (1, 5 and 35) of the channel-delay cross-correlation matrix	3
F5	Extremities velocity	Average peak velocities of the lower body extremities (ankles)	2
F6	Walk complexity	Human movement complexity metric and degrees of freedom to explain 90% variance	2
F7	Legʼs root mean square power spectrum	Average energy per walk cycle of the hip and knee joint velocities	6
F8	Joint velocities correlation coefficient	Pearsonʼs correlation coefficients between lower body joints	9
F9	Head spine movement plane area	Area and variability of the head movements on the frontal and sideways plane	3
F10	Average upper body joint velocity	Average joint angular velocities of the shoulder and elbow joints	5
F11	Upper body complexity	Human movement complexity metric and degrees of freedom to explain 90% variance of the upper body joint velocities	2
F12	Workspace probability density volume and entropy	Volume occupied by the joints calculated using the 3D location of the joints of the upper body	2
F13	Upper body autocorrelation full width at half-maximum	The width of the autocorrelation curve (of the joint angular velocities of the upper body joints) at the point when it reaches a value of 0.5	5
F14	Channel-delay cross-correlation	Eigen spectrum values (1, 5, 30 and 300) of the channel-delay cross-correlation matrix	4
F15	Arm root mean square power spectrum	Average energy of the shoulder and elbow joint angular velocities	5
F16	Wrist average velocity	Average velocities of the wrist in space	1
F17	Logistic fit on upper body jointsʼ velocity	Scale parameter of the logistic distribution of upper body jointʼs angular velocities and wrist’s velocity in space	8
F18	Head spine movement plane area	Area of the head movements on the frontal and sideways plane	1

### Validation of features

**Extended Data Fig. 1:**
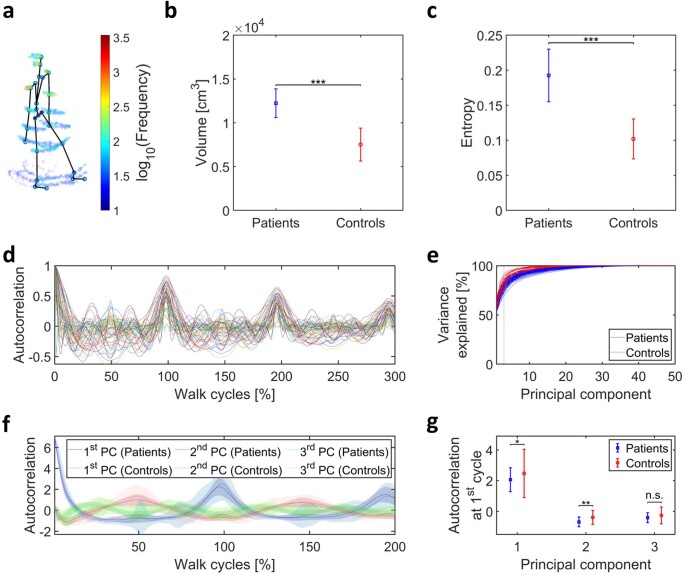
Suit features from the 8 MW task. **a**. The density plot generated using the 3D location of joints from a typical FA patient. The space is segmented in blocks of size 2 × 2 × 2 cm. Note that the color bar is in log10 scale. **b**. The workspace volume calculated using the density plot is significantly higher for FA Patients than Controls suggesting a more irregular movement with more compensatory mechanisms in place. Data are presented as mean ± standard deviation (n = 62 patient measurements and n = 18 control measurements) and ∗ represents p ≤ 0.05, ∗∗ is p ≤ 0.01 and ∗∗∗ is p ≤ 0.001 for Kruskal-Wallis one-way ANOVA. For the exact p-values of the ANOVA tests, see Supplementary Table 2. **c**. The FA Patients’ entropy showing a significant increase with respect to Controls thus suggesting a more disordered and less predictable walking pattern. Data are presented as mean ± standard deviation (n = 62 patient measurements and n = 18 control measurements) and ∗ represents p ≤ 0.05, ∗∗ is p ≤ 0.01 and ∗∗∗ is p ≤ 0.001 for Kruskal-Wallis one-way ANOVA. For the exact p-values of the ANOVA tests, see Supplementary Table 2. **d**. The autocorrelation of a typical FA patient performing the 8 MW task calculated from the suits’ 51 DoF for up to a lag of 3 walk cycles. **e**. The variability explained when applying a PCA analysis on the autocorrelation signals showing that the first three Principal Components (PCs) can explain more than 80% of the variability in data. **f**. The first three PCs of the autocorrelation signal for FA Patients (solid lines) and Controls (dashed lines). Data are presented as mean ± standard deviation (n = 62 patient measurements and n = 18 control measurements) **g**. A comparison of the value of the autocorrelation PCs after the 1st walk cycle, showing a significant difference between FA Patients and Controls (Kruskal-Wallis one-way ANOVA). Data are presented as mean ± standard deviation (n = 62 patient measurements and n = 18 control measurements) and ∗ represents p ≤ 0.05, ∗∗ is p ≤ 0.01 and ∗∗∗ is p ≤ 0.001 for Kruskal-Wallis one-way ANOVA. For the exact p-values of the ANOVA tests, see Supplementary Table 2.

**Extended Data Fig. 2:**
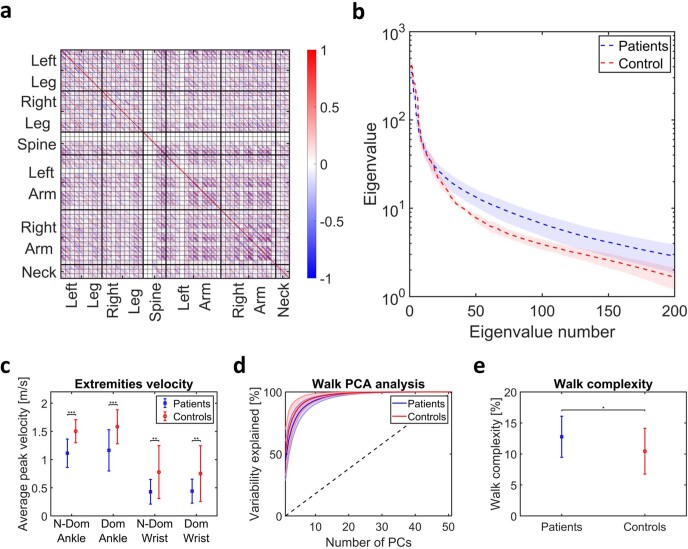
Suit features from the 8 MW task. **a**. The channel delay cross-correlation matrix for a typical FA patient. It is a 51 x 51 block matrix where the blocks along diagonal contain the joint angular velocity within- channel correlations and off-diagonal blocks contain the correlations across different joints. Within each block, the correlations are calculated for 10 different lags (linearly spaced from zero up to the subject’s average walk cycle). **b**. The average eigen spectrum for FA Patients and Controls showing a decreased power in the patients’ lower eigenvalues that reflects the increased dynamical complexity in their walk. The dotted lines represent the mean and the shade along the dotted line represents the standard deviation. **c**. The averaged peak velocity of subjects’ extremities during the 8 MW task showing a statistical difference between FA Patients and Controls on the D & ND ankles and on the ND wrist (Kruskal-Wallis one-way ANOVA). Data are presented as mean ± standard deviation (n = 62 patient measurements and n = 18 control measurements) and ∗ represents p ≤ 0.05, ∗∗ is p ≤ 0.01 and ∗∗∗ is p ≤ 0.001 for Kruskal-Wallis one-way ANOVA. For the exact p-values of the ANOVA tests, see Supplementary Table 2. **d**. The variability explained by combining a different number of PCs based on a PCA analysis of the subjects’ angular velocities. The blue and red lines represent the mean results obtained for the FA Patients and Controls respectively while the shade along the lines represents the standard deviation. **e**. Our human movement complexity metric applied to all subjects showing a statistically higher walk complexity in the FA Patients than Controls (Kruskal-Wallis one-way ANOVA). Data are presented as mean ± standard deviation (n = 62 patient measurements and n = 18 control measurements) and ∗ represents p ≤ 0.05, ∗∗ is p ≤ 0.01 and ∗∗∗ is p ≤ 0.001 for Kruskal-Wallis one-way ANOVA. For the exact p-values of the ANOVA tests, see Supplementary Table 2.

**Extended Data Fig. 3:**
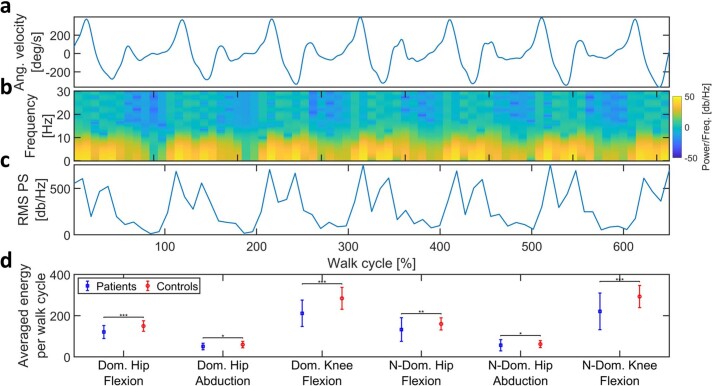
Power spectrum of the joint velocities. **a**. The angular velocity from the knee joint of a typical FA patient performing the 8 MW task. **b**. The Short-Time Fourier Transform (STFT) of the joint velocities on a 200 ms window with 100 ms overlap. **c**. The RMS of the power spectrum coefficients on each window which summarizes the information of the power spectrum. **d**. A comparison between the average energy per walk cycle of FA Patients and Controls per individual joint, showing a statistically higher energy on the D hip & knee flexion and the ND knee flexion of Controls (Kruskal-Wallis one-way ANOVA). Data are presented as mean ± standard deviation (n = 62 patient measurements and n = 18 control measurements) and ∗ represents p ≤ 0.05, ∗∗ is p ≤ 0.01 and ∗∗∗ is p ≤ 0.001 for Kruskal-Wallis one-way ANOVA. For the exact p-values of the ANOVA tests, see Supplementary Table 2.

**Extended Data Fig. 4:**
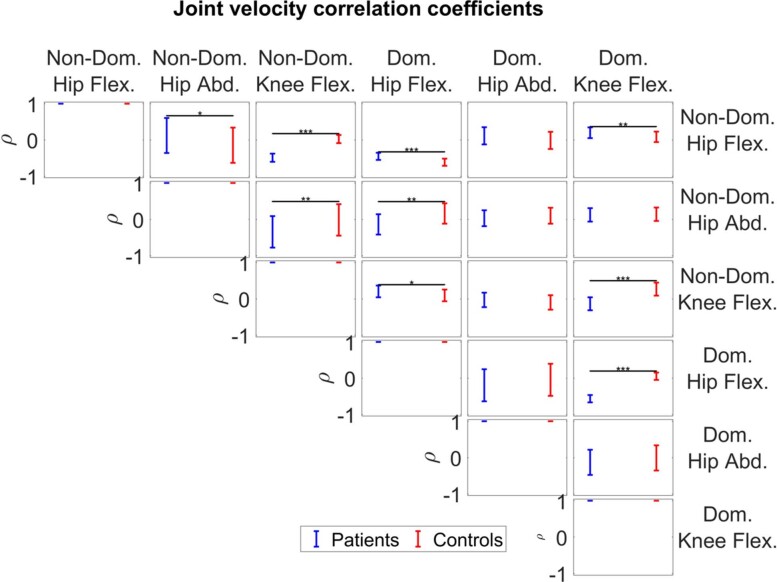
Correlation coefficients of the joint velocities. The Pearson’s product-moment correlation coefficients (ρ) between different lower- body joints separated by leg dominance with most showing a statistical difference between FA Patients and Controls (Kruskal-Wallis one-way ANOVA). Data are presented as mean ± standard deviation (n = 62 patient measurements and n = 18 control measurements) and ∗ represents p ≤ 0.05, ∗∗ is p ≤ 0.01 and ∗∗∗ is p ≤ 0.001 for Kruskal-Wallis one-way ANOVA. For the exact p-values of the ANOVA tests, see Supplementary Table 2.

**Extended Data Fig. 5:**
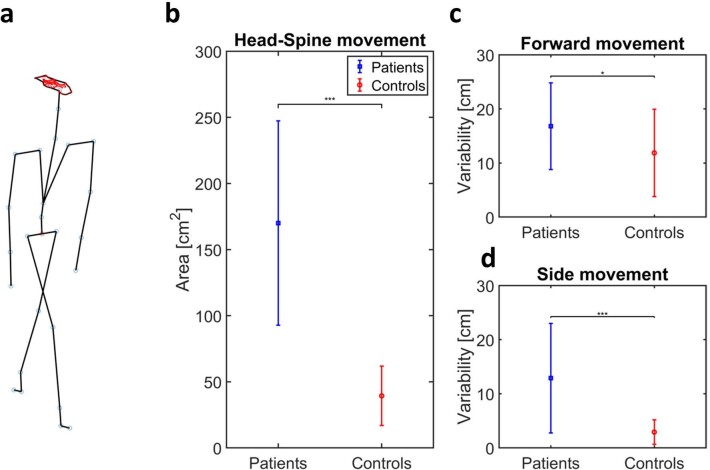
Head-spine movement. **a**. The head movement trajectory from a typical FA patient (red line) with respect to a fixed hip during the 8 MW task. **b**. The FA Patients’ head movements exhibit a statistically higher area than the Controls. Data are presented as mean ± standard deviation (n = 62 patient measurements and n = 18 control measurements) and ∗ represents p ≤ 0.05, ∗∗ is p ≤ 0.01 and ∗∗∗ is p ≤ 0.001 for Kruskal-Wallis one-way ANOVA. For the exact p-values of the ANOVA tests, see Supplementary Table 2. **c-d**. Observing the head movements on the frontal and sideways plane independently shows that there is a statistical difference between FA Patients and Controls only when the variability of sideways movements is considered. Data are presented as mean ± standard deviation (n = 62 patient measurements and n = 18 control measurements) and ∗ represents p ≤ 0.05, ∗∗ is p ≤ 0.01 and ∗∗∗ is p ≤ 0.001 for Kruskal-Wallis one-way ANOVA. For the exact p-values of the ANOVA tests, see Supplementary Table 2.

**Extended Data Fig. 6:**
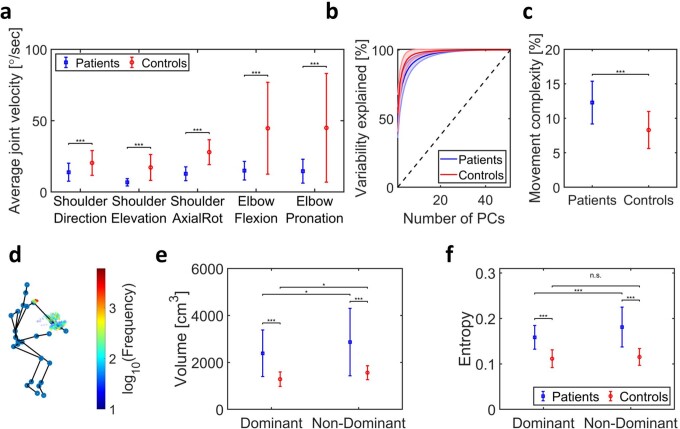
Suit features from the 9HPT task. **a**. The average joint velocity of FA Patients and Controls during the 9HPT. The patients exhibit significantly slower velocities in all joints. Data are presented as mean ± standard deviation (n = 66 patient measurements and n = 18 control measurements) and ∗ represents p ≤ 0.05, ∗∗ is p ≤ 0.01 and ∗∗∗ is p ≤ 0.001 for Kruskal-Wallis one-way ANOVA. For the exact p-values of the ANOVA tests, see Supplementary Table 3. **b**. A PCA analysis of the angular velocities of the upper body showing the variability explained by having a different number of PCs. FA Patients require slightly more PCs to explain the variability in their movements than the Controls. Data are presented as mean ± standard deviation (n = 66 patient measurements and n = 18 control measurements). **c**. Our complexity metric showing an increased complexity in the FA Patients during the 9HPT. Data are presented as mean ± standard deviation (n = 66 patient measurements and n = 18 control measurements) and ∗ represents p ≤ 0.05, ∗∗ is p ≤ 0.01 and ∗∗∗ is p ≤ 0.001 for Kruskal-Wallis one-way ANOVA. For the exact p-values of the ANOVA tests, see Supplementary Table 3. **d**. A density plot generated using the kinematics of a typical FA patient during the 9HPT. The space was separated in 2x2x2cm blocks and the color of each block represents the time any joint (blue circles) spent in that area. Note that the color bar is on log10 scale. **e**. The workspace volume generated from the subjects’ density plot comparing the performance of FA Patients and Controls when performing the task with either hand. The plot shows a statistical difference between the two hands of FA Patients as well as a significant difference between the workspace volume generated by the FA Patients’ and Controls’ dominant hand. Data are presented as mean ± standard deviation (n = 66 patient measurements and n = 18 control measurements) and ∗ represents p ≤ 0.05, ∗∗ is p ≤ 0.01 and ∗∗∗ is p ≤ 0.001 for Kruskal-Wallis one-way ANOVA. For the exact p-values of the ANOVA tests, see Supplementary Table 3. **f**. The entropy of subjects’ density plots as a measure of their disorder showing that FA Patients have a lot more disorder than Controls across both hands. Data are presented as mean ± standard deviation (n = 66 patient measurements and n = 18 control measurements) and ∗ represents p ≤ 0.05, ∗∗ is p ≤ 0.01 and ∗∗∗ is p ≤ 0.001 for Kruskal-Wallis one-way ANOVA. For the exact p-values of the ANOVA tests, see Supplementary Table 3.

**Extended Data Fig. 7:**
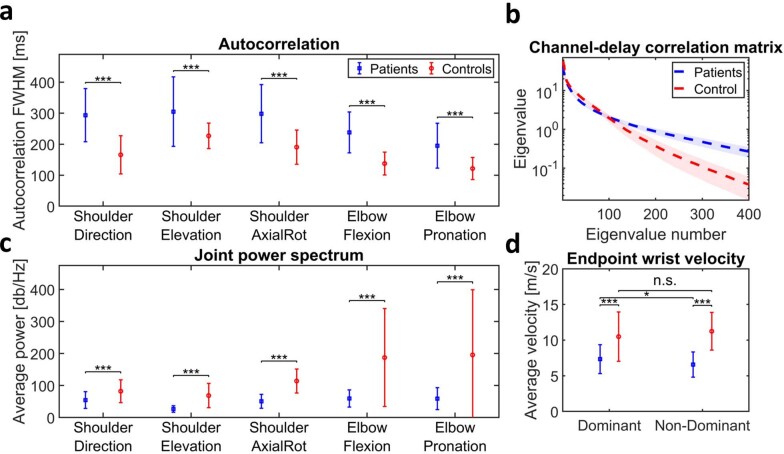
Suit features from the 9HPT task. The autocorrelation full-width at half-maximum (FWHM) of the subjects upper body joints showing a significantly slower drop for the FA Patients than the Controls in most joints’ dimensions. Data are presented as mean ± standard deviation (n = 66 patient measurements and n = 18 control measurements) and ∗ represents p ≤ 0.05, ∗∗ is p ≤ 0.01 and ∗∗∗ is p ≤ 0.001 for Kruskal-Wallis one-way ANOVA. For the exact p-values of the ANOVA tests, see Supplementary Table 3. **b**. The averaged eigen spectrum based on the subjects’ channel delay cross-correlation matrices, showing that FA Patients have a decreased power in the left end and increased power in the right end. This implies that they have an increased complexity in their movements with respect to the Controls. Data are presented as mean ± standard deviation (n = 66 patient measurements and n = 18 control measurements. **c**. A comparison on the average power of the upper body joints between FA Patients and Controls, showing a statistically lower energy in all FA Patient joints except the elbow pronation. Data are presented as mean ± standard deviation (n = 66 patient measurements and n = 18 control measurements) and ∗ represents p ≤ 0.05, ∗∗ is p ≤ 0.01 and ∗∗∗ is p ≤ 0.001 for Kruskal-Wallis one-way ANOVA. For the exact p-values of the ANOVA tests, see Supplementary Table 3. **d**. The subjects’ average wrist speed during the 9HPT task showing a statistical difference between FA Patients and Controls but also a difference between the D and ND hand of FA Patients. Data are presented as mean ± standard deviation (n = 66 patient measurements and n = 18 control measurements) and ∗ represents p ≤ 0.05, ∗∗ is p ≤ 0.01 and ∗∗∗ is p ≤ 0.001 for Kruskal-Wallis one-way ANOVA. For the exact p-values of the ANOVA tests, see Supplementary Table 3.

**Extended Data Fig. 8:**
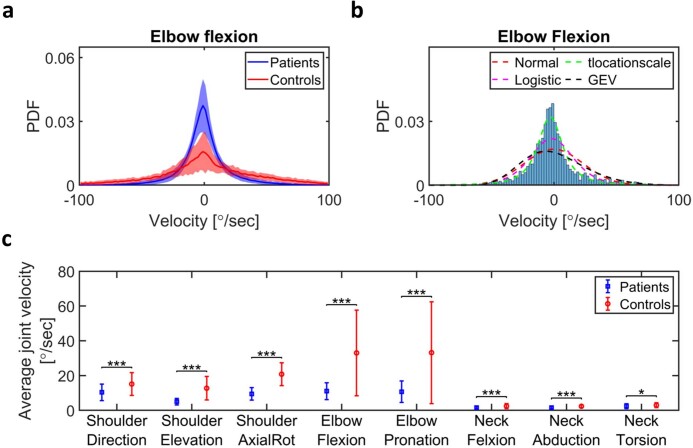
Logistic fit on joints' angular velocities. **a**. A comparison between the probability density functions (PDF) of angular velocities for a typical joint (elbow flexion) showing a much wider spread in Controls than the FA Patients. This effect has been consistently observed across all upper body joints. The lines represent the mean and the shaded area around the lines represents the standard deviation in each population group. **b**. We fitted various parametric probability distributions on the joints’ angular velocities, and we found that a logistic distribution can provide the best fit in the data. **c**. A comparison between the variability (σ) of the logistic fit between the FA Patients and Controls after applying it on all upper body joints independently. The results reveal a much larger σ in some of the Controls’ joints meaning that they used a much wider range of velocities during the 9HPT than the FA Patients. Data are presented as mean ± standard deviation (n = 66 patient measurements and n = 18 control measurements) and ∗ represents p ≤ 0.05, ∗∗ is p ≤ 0.01 and ∗∗∗ is p ≤ 0.001 for Kruskal-Wallis one-way ANOVA. For the exact p-values of the ANOVA tests, see Supplementary Table 3.

**Extended Data Fig. 9:**
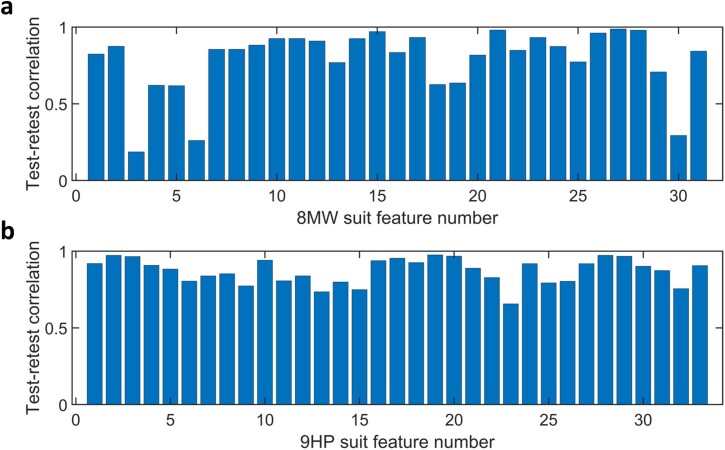
Test-retest correlation of suit features. The 8 MW and 9HPT tasks were repeated twice as part of the SCAFI assessment and the suit features were calculated for each repeat of the tasks and the test-retest correlation for the suit features from the 8 MW task (**a**) and the 9HP task (**b**) was calculated between both the repeats for all visits of 9 FA patients and 9 controls.

A detailed explanation of all these features is presented in the Methods section (and Extended Data Figs. 1–8) and their validation by test–retest correlation is presented in Extended Data Fig. 9. Most of these features are substantially different between FA patients and controls thereby quantitatively capturing the effects of FA on movement. To develop a clinically useful and improved measure of deterioration, we set out to predict the continuous values of the clinical scales quantitatively. We calculated Pearsonʼs correlation coefficient of each feature with respect to the SARA and SCAFI scales and most of the features presented absolute correlations in the range of 0.3–0.5 with respect to the two clinical scales. Therefore, none of them can be independently used for monitoring disease progression. However, a more robust prediction can be potentially achieved by combining all these behavioral features—the same way as is applied in the standard clinical scales.

The relationships linking our features and clinical scales are nonlinear. Hence, we used a Gaussian Process (GP) Regression algorithm to find the mapping between the extracted behavioral features and the SARA and SCAFI clinical assessments. GP regression is a state-of-the-art method that applies a nonlinear regression and can capture the uncertainty in the presence of high variability in the data in a principled manner^39^.

### Cross-sectional predictions of SARA and SCAFI

**Fig. 2:**
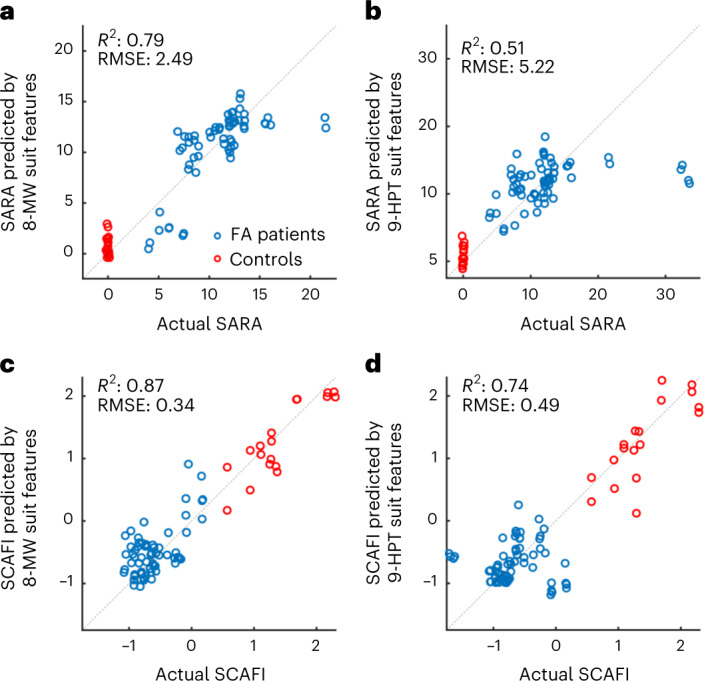
Cross-sectional predictions of SARA and SCAFI scores. **a**–**d**, Actual SARA (**a**,**b**) or actual SCAFI (**c**,**d**) scores are plotted versus predicted values, as derived from the cross-sectional prediction of the clinical scales using suit features from the 8-MW (**a**,**c**) and 9-HPT (**b**,**d**) tasks. Each data point represents a single actual versus predicted score of an FA patient (9 participants, 33 visits) or control (9 participants, 9 visits). One participant could not perform the 8-MW task and performed only the 9 HPT. Two data points were obtained per clinical visit of each participant, as two suit measurements for both 8 MW and 9 HPT were obtained from each visit. A small jitter is added to the points to show overlapping points. A leave-one-subject-out cross-validation and GP regression were used for the predictions of the SARA and SCAFI scores.

Firstly, we did a cross-sectional prediction of the clinical assesments using the suit features from the corresponding visits and the leave-one-participant-out cross-validated results are shown in Fig. 2a–d. The algorithm achieved a coefficient of determination (*R*^2^) of 0.79 and a root mean square error (RMSE) of 2.49 when predicting SARA scales using suit features of 8 MW and an *R*^2^ of 0.51 and an RMSE of 5.22 when using suit features of 9 HPT. When predicting SCAFI, the algorithmʼs performance increased in both the cases of 8-MW and 9-HPT suit features with *R*^2^ of 0.87 and 0.74, respectively. It should be noted that one participant could not do the 8-MW test and did only the 9 HPT. This establishes that our methodology can be used to predict the clinical scales for nonambulatory patients too. The challenge with leave-one-subject-out cross-validation is that every time the algorithm is tested it is on a new subject with completely new dynamics. Nevertheless, the suit features can still predict the disease state of the patients with good accuracy. The features selected by the feature selection algorithm for predicting SARA and SCAFI are presented in Supplementary Figs. 1 and 2.

Because our patients do not cover the whole range of the clinical scores (0–40), the algorithmʼs performance is not very good in predicting the SARA scores at the higher end of the scale (for example, the two sporadic values at the top right corner of the plot). It is clear from the results that the GP regression performance can be improved with a bigger dataset. It can also be observed that both the 8-MW and 9-HPT suit features are better at predicting SCAFI when compared to SARA. This should not be surprising as both 8 MW and 9 HPT are part of the SCAFI test suite and the suit features of the 8-MW and 9-HPT subtasks will have more predictive power at predicting the SCAFI score.

### Longitudinal predictions of SARA and SCAFI

**Fig. 3:**
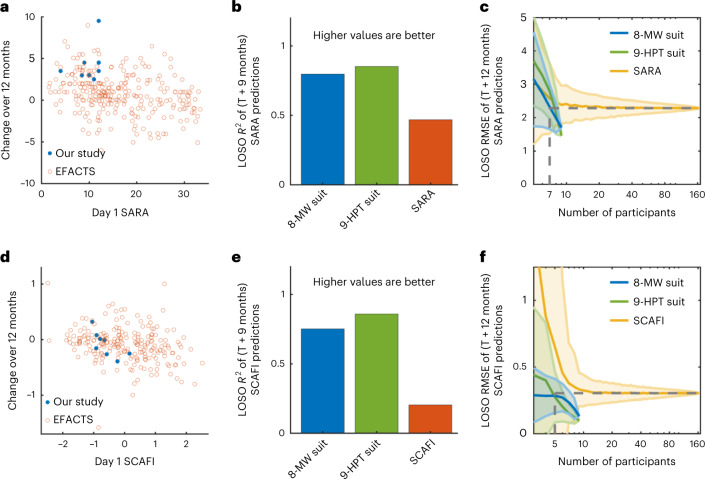
Longitudinal predictions of SARA and SCAFI scores. **a**, Scatter plot of the day 1 SARA score versus the change in the SARA score over 9 months for data from participants from our study (*n* = 8) and the change in the SARA score over 12 months for patients from the EFACTS study (*n* = 164). A small jitter is added to the points to show overlapping points. **b**, Comparison of *R*^2^ values of leave-one-subject-out (LOSO) cross-validated predictions of SARA scores at 9 months after initiation of our study using suit features from the 8-MW and 9-HPT tasks, and SARA scores from the first visit of our study **c**, Plot of the mean aggregate leave-one-subject-out cross-validated RMSE of the predictions of SARA scores at 9 months after initiation of our study or 12 months after study initiation for the EFACTS study, as a function of the number of participants used to build the machine learning models. The vertical dotted lines refer to the number of participants needed to build models using suit features that have the same performance as the models built using SARA scores from 164 participants. The shaded regions indicate the standard deviation of the results from different models built for each number of participants. **d**–**f**, Corresponding data for the SCAFI scores are shown.

We then wanted to analyze how well the kinematic features extracted from the suit data of 8 MW and 9 HPT can accurately predict the longitudinal disease progression occurring in FA patients when compared to scales obtained following conventional assessment by clinicians (Fig. 3a–f). First, we wanted to understand how the clinical scales change over a year as a function of their day-1 clinical scale. In Fig. 3a,d, we have plotted the change in SARA and SCAFI scales, respectively, against their day-1 clinical scale for the FA patients of our study and also patients from EFACTS study^26^ (a two-year longitudinal study with a larger cohort size).

We used GP regression to predict the month-9 SARA and SCAFI scales of the participants from our study using the suit features from day-1 8-MW and suit features from day-1 9 HPT as predictors. We then compared these longitudinal predictions against the predictions of the month-9 SARA and SCAFI scales using the day-1 SARA and SCAFI scales from our study (Fig. 3b,e). For the longitudinal predictions of SARA, both the day-1 suit features of 8 MW and 9 HPT achieved a good leave-one-subject-out cross-validated *R*^2^ of 0.80 and 0.85 in comparison with an *R*^2^ of 0.47 using day-1 SARA. Again, for the longitudinal predictions of SCAFI, the 8-MW and 9-HPT day-1 suit features outperformed day-1 SCAFI (*R*^2^ of 0.75 and 0.86 versus 0.21). Please see Supplementary Fig. 3 for plot of the RMSE of the results. This implies that our suit features contained sufficiently rich information not only to score the disease state of the patient in the present but also to predict how the disease would evolve. The features selected by the feature selection algorithm for the longitudinal predictions of SARA and SCAFI are presented in Supplementary Figs. 4 and 5.

We then plotted the RMSE of the longitudinal predictions as a function of number of participants used to build the machine learning model (Fig. 3c,e). Here we used the data from the larger EFACTS study to build the models using SARA and SCAFI as predictors to allow a larger data size for the models using the clinical scales. The model using suit features achieved better performance with a smaller number of participants (*n* = 7) compared to the model using the clinical scales with a larger cohort from the EFACTS study (*n* = 164). This establishes that a small population size is sufficient to build prediction models with high accuracy when using the rich set of suit features, which would therefore substantially reduce the numbers of patients required in the context of drug development.

### Cross-sectional prediction of *FXN* mRNA levels

**Fig. 4:**
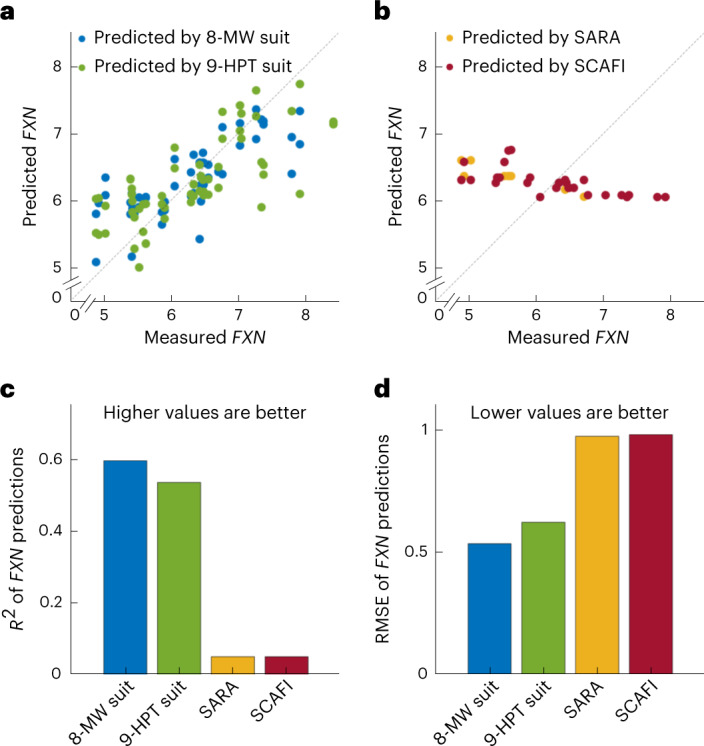
Cross-sectional predictions of *FXN* gene expression. *FXN* mRNA levels were predicted using the following four sets of predictors: suit features from the 8-MW task, suit features from the 9-HPT task, SARA and SCAFI scores. **a**, Scatter plot of measured *FXN* mRNA levels against *FXN* mRNA levels predicted by the suit features from the 8-MW and 9-HPT tasks of the corresponding visits. Two data points were obtained per clinical visit of each participant, as two suit measurements for both 8 MW and 9 HPT were obtained from each visit. **b**, Scatter plot of measured *FXN* mRNA levels against *FXN* mRNA levels predicted by the SARA and SCAFI clinical scales of the corresponding visits. **c**,**d**, Leave-one-subject-out cross-validated *R*^2^ (**c**) and RMSE (**d**) values of the predictions by regression models using the features of the suit data of 8-MW and 9-HPT tasks, SARA and SCAFI scales as predictors.

FA is caused by a GAA-repeat expansion in the *FXN* gene leading to transcriptional repression of *FXN* and the disease. The length of the shorter GAA-repeat has been shown to correlate inversely with the age of onset^10,26^. We have confirmed this in our data (Supplementary Fig 6) and shown that this correlation improves when removing patients with interruptions in the short GAA repeat length^40^. We cross-sectionally predicted the *FXN* mRNA levels of the participants using four sets of predictors: 8-MW suit features, 9-HPT suit features, SARA and SCAFI, and the results for the leave-one-subject-out cross-validation are presented in Fig. 4a–d. Suit features of 8 MW and 9 HPT achieved an *R*^2^ of 0.59 and 0.53 (and an RMSE of 0.53 and 0.62) for the leave-one-subject-out cross-validated case. In comparison, both SARA and SCAFI achieved only *R*^2^ values close to zero (with RMSE values of 0.97 and 0.98). (Please see Supplementary Fig. 7a,b for the features selected by the feature selection algorithm for the prediction of *FXN* and Supplementary Fig. 7c for a scatter plot of the *FXN* against SARA and SCAFI).

The total scores of SARA and SCAFI might be poorer at prediction because they contain less information. We reasoned that by using the individual components of the SARA and SCAFI scales as predictors, we would improve the predictive capacity (Supplementary Fig. 8). Although this led to an improvement (*R*^2^ of predictions using the components of SARA increased to 0.36 and that of SCAFI increased to 0.20), the prediction using the suit features of the 8 MW (*R*^2^ of 0.59) and 9 HPT (*R*^2^ of 0.53) still outperformed the individual components of SARA and SCAFI in predicting *FXN* levels.

## Discussion

Clinical scales, which attempt to quantify the clinical examination, are widely used as gold-standard neurological and cardiovascular assessments. These are often measured ‘by-eyeʼ and are subject to intrarater variability (the same assessors reporting different outcomes on repeated trials)^7^, inter-rater variability (different assessors scoring the same assessment differently)^41^ and variability in the patient performance ability (which may vary based on the time and days of the week, seasons, etc.). Such clinical scales are composed of a battery of different behavioral subassessments (for example, walking and picking up things) to capture the performance across different domains of the bodyʼs capabilities and average out variability across individual subassessments (for example, FA SCAFI has three subassessments whereas SARA has eight subassessments). This results in longer times to perform clinical tests and more opportunity for rater variability. Moreover, variability from each subassessment may, in the worst case, be additive.

We show here that the kinematic features from one test can predict the clinical scales of another test and future scores. and indeed, the key molecular marker—*FXN* levels, indicating that they carry crucial information about the disease and can directly link behavior with *FXN* gene expression. Our data-driven approach also revealed new observational information that can complement the current clinical evaluations. For example, one of our main findings, which was supported by multiple metrics, was the substantially reduced movement structure and complexity in the patientsʼ activities compared to controls during the two subtests. The high accuracy of the collected data can also be used to better understand the subtle changes in behavior secondary to the disease or ameliorated by therapeutic intervention.

Our features quantify the complex and subtle movement patterns that may escape even very experienced clinicians. The larger workspace volume and the entropy of the workspace density of the FA patients quantify the more disordered movement patterns of FA patients. Ataxia patients develop complex compensatory movement mechanisms to balance out their sensorimotor dysfunction and this is captured by our movement complexity metric. The low principal component (PC) values of the autocorrelations of the joint angular velocities reflect the low repeatability of body postures while walking in FA patients and what clinicians subjectively characterize as the ataxic gait. The reported muscle weakness in the arms and legs of FA patients is consistent with the low power spectrum values of the hip and joint velocities. The qualitative ‘by-eyeʼ observations of the swaying of the head of the FA patients are captured by our planar movement area feature.

The fact that the kinematic features of each of the subtasks (8 MW and 9 HPT) can predict the overall clinical scores with good accuracy suggests that our data-driven approach can predict the overall performance of a patient with a minimal number of tasks in the clinical assessment although they will have to have the suit fitted, which takes about 10 min. A further benefit is that subjective, by-eye measurements are replaced with data-rich, digitally accurate data. Our data-driven analysis also allowed us to characterize the kinematic alterations in FA patients and verify and objectify the various observations that clinicians use in their verbal characterization such as slower irregular movements.

Recent efforts^42–46^ used movement sensors in an attempt to overcome the errors caused by subjective clinical scales. Patients with Parkinsonʼs disease^47^ donned full-body suits to capture the patientsʼ behavior during clinical tasks. The analysis on cross-sectional classification of the severity of the disease (mild versus severe) rather than capturing changes in the disease progression through time. Similarly, a recent cross-sectional study used wearable motion sensors to quantify the SARA scale and thereby identify very early signs in the spinocerebellar ataxias^48^. Here we go further in developing algorithms to accurately predict outcomes longitudinally and moreover predict a molecular biomarker of FA.

The digital kinematic features we have developed do not depend on a specific ’suitʼ or even the use of wearable sensors. The only information needed for the generation of the kinematic features is the skeletal movement data, that is, time series of body poses. The kinematic features are agnostic to the source of the skeletal movement data. We believe that as technology progresses for the accurate skeletal reconstruction of human movement from video, remote clinical evaluation of patientsʼ motor performance, even in a home setting, will become possible.

This work promises to shorten clinical trials that would otherwise be prohibitively long or reduce the number of patients required to measure the deterioration of the neurological state. The latter is especially important in rare neurodegenerative diseases. Therefore, such technology is likely to facilitate the possibility of finding treatment for these relatively neglected and incurable conditions.

The study presented here was exploratory in nature and has provided proof of concept that this approach would outperform clinical scales in a clinical trial setting. Notwithstanding the results, our study has some limitations. Our cohort consisted of predominantly late-onset ambulatory patients. Further studies are needed to validate our technology and methodology in a larger and wider demographic cohort with participants across different ambulatory statuses. Also, the superiority of our digital kinematic features over the existing clinical scales needs to be established in interventional drug trials of FRDA and other motor diseases. We have already demonstrated the usefulness of our kinematic features in a different patient group, children, for another degenerative disease^49^. Further studies will establish this in a larger and wider cohort and will also investigate the use of kinematic features from daily life activities at home. We believe that these features have the potential to be universal and not only recognize disease progression in one disease but also allow us to distinguish between diseases and potentially multimorbidities.

In addition, the finding that the motion capture features could be used to predict the *FXN* mRNA level, in contrast to the clinical scales, was an interesting result as the kinematics do change over time and *FXN* levels do not change appreciably. Consistent with our results, a previous study could also correlate clinical scales with *FXN* levels (*R*^2^ of 0.24 for FARS)^50^. Our population consisted of predominantly late-onset ambulatory patients, which may have contributed to the accuracy of the predictions (*R*^2^ of 0.53). Another clinically relevant possibility is that *FXN* levels might be able to predict disease trajectory and further studies are required to address this. Notwithstanding, our results are consistent with the knowledge that FXN deficiency causes movement disorder in FA.

We show here that FA provides a model neurodegenerative condition where a holistic machine learning analysis of full-body kinematics from a longitudinal study demonstrates accurate prediction of dysfunction progression in individual patients. We not only determine clinical phenotype but also predict the molecular cause of the disease (repressed Frataxin) cross-sectionally from movement data alone, albeit on a restricted population. While high-resolution behavioral genomics was previously successful in genetic model organisms such as *Caenorhabditis elegans*^45^ at differentiating mutants through pure digital behavioral analysis and was able to pick up subtle changes in physiological and reproductive state from the movement behavior of *Drosophila* *Melanogaster*^46^, such data-driven approaches have been lacking in human clinical and genomic applications. Existing digital biomarkers applied in human clinical settings involve supplanting conventional measures such as distance walked on a treadmill with digitally measured proxies obtained through digital devices (such as step counters). However, these approaches overlook the richness of signal contained in full-body kinematic data and instead revert to reusing existing clinical measures. To date, the monitoring of neurodegenerative disease progression frequently fails to adequately test disease-modifying therapies because it is slow and of low precision, making drug development risky and expensive. Our digital behavioral biomarker approach promises to be of benefit to patients with rare diseases where potential disease-modifying treatments are becoming available.

## Methods

### Subjects and measurements

Nine FA patients and nine age- and sex-matched controls participated in this study. FA patients had all been diagnosed with Friedreichʼs ataxia based on clinical criteria and a genetically confirmed GAA-repeat expansion on both alleles of the *FXN* gene. More information on the patient selection process is available in ref. ^19^. Study participants had provided written informed consent before any study-related procedures were initiated. Consent was obtained from the individual whose image is shown in Fig. 1 for publication of the image. Our clinical trial was approved by the UK Medicines and Healthcare Products Regulatory Agency (EudraCT 2011-002744-27), the Riverside Research Ethics Committee (11/LO/0998) and the Imperial College London Joint Research & Compliance Office (see supplementary note for the study protocol). All measurements were done at our clinical facility (NIHR Imperial Clinical Research Facility, Imperial Centre for Translational and Experimental Medicine, Hammersmith Hospital, London).

Measurements were taken four times from the FA patients and once from the controls during the course of the study (day-1/baseline, after 3 weeks and 3 and 9 months) and this enabled collection of behavioral data that can be used in monitoring the progression of the ataxic disease on a longitudinal scale. One of the FA patients dropped out of the study after second visit due to personal reasons; however, we still included the collected data in further analysis where possible. Motion capture suit data was not collected during the last visit of another patient because of technical issues. A blood sample for *FXN* measurement was not collected for a patient. The characteristics of the participants in our study are listed in Table 1.

The EFACTS study data comes from a longitudinal study^26^ of a large cohort of Friedreichʼs ataxia patients from 11 European study sites where the patients were seen at baseline, 1 and 2 years, and the different clinical scales were recorded. All our study participants have an onset of FA over 18 years of age (except one participant who had an age of onset of 15 years). We, therefore, included only participants from EFACTS who had onset after 18 years. The characteristics of the EFACTS study participants included in our analysis are listed in Supplementary Table 1. The EFACTS study (ClinicalTrials.gov Identifier, NCT02069509) was approved by the local ethics committees of each participating site in the study. All patients or their authorized surrogates provided written informed consent upon enrollment into EFACTS.

### Wearable full-body motion capture system (the ’suitʼ)

The movement from the entire body was recorded using an IGS-180 motion capture suit (Animazoo). The suit consists of an elastic Lycra trouser and jacket with 17 sensors embedded into the fabric to measure the movement of various limbs. The sensors are nine-axis inertial measurement units and the data from all sensors can be streamed wirelessly to a laptop at 60 Hz. The calibration of the motion capture suit is performed using a simple routine provided by the manufacturer as part of the control software. The suit reports the motion data fused with a skeleton structure in a Biovision hierarchical data (BVH) format, which saves each jointʼs position as Euler angles. In Fig. 1d, we show a FA patient performing the 8-MW clinical assessment while wearing the motion capture suit. The same figure also shows a few of the reported joint angular positions throughout the task and a reconstruction of the body posture at a single frame.

### Standard operating procedures for the suit

The suit required the assistance of a trained health professional (not the carer) when putting it on or taking it off during the experiments, to avoid damaging the sensors in the process. First, the participants took off their shoes and trousers and put on the suit trousers (we allowed participants to wear tights, leggings or other tight-fitting clothes underneath). The suit trousers were tightened by adjusting the Velcro straps around the hips, thighs and calves. The participants then put the shoes back on and the foot sensors were attached on top of each foot using Velcro straps. Afterward, the participants took off any heavy clothes worn on top (jackets, jumpers or any loose/warm clothes) and put on the suitʼs jacket. The trouser cables were connected to the splitters inside the jacket and the jacket was zipped and tightened using the Velcro straps on both sides of the trunk, the upper and the lower arms. The jacket was also attached to the trousers using Velcro patches. Then the cap was placed on the participantsʼ head with the sensor on the left-hand side. Finally, the transmitter was connected to the suit and was placed in the left front pocket along with the battery pack. Taking the suit off followed exactly the reverse process. All these steps are part of our Standard Operating Procedures in accordance with the Good Clinical Practice standards, and they have been approved for studies involving monitoring of patients with neurodegenerative disorders. These steps ensured patientsʼ safety during the experiments and protected the integrity of the suit.

### Standard FA clinical assessments (SARA and SCAFI clinical scales)

We used two clinical assesments, the SARA and the SCAFI. The SARA has been found to be one of the most appropriate ways to measure disease severity and progression in FA patients^51^. SARA is a discrete scale scored from 0 (no ataxia) to 40 (severe ataxia) and it is based on an aggregate score of the following eight subtests: gait (0–8 score), stance (0–6 score), sitting (0–4 score), speech disturbance (0–6 score), lack of coordination in finger chase (0–4 score), tremor during nose to finger test (0–4 score), fast alternating hand movements (0–4 score) and heel-shin slide (0–4 score)^22^. The SCAFI is a continuous index composed of the following three time-based subtests: the 8 MW, the 9 HPT and the rate of repetition of the phrase ‘PATAʼ over 10 s (PATA). These components are then *z*-scored across all participants and expressed as a standard deviation from the baseline mean. Being further away from the baseline suggests a more severe ataxia^23^.

We focused on the analysis of the 8 MW and 9 HPT, two simple scenarios that are closely related to activities we perform on a daily basis (that is, walking around in the house and performing tasks while seated on a table).

The 8-MW clinical subscale is measured as the time needed to walk an 8 m distance with any assistive device as quickly as possible but safely (without the help of another person). The 8 MW is measured from standing with feet behind the start line until one of the legs reaches the 8-m mark^23^. Figure 1d presents a typical 8-MW time-lapse (every frame shown is 0.5 s apart) as collected by the motion capture suit. The second clinical subscale we focused on is the 9 HPT, which is defined as the time taken by a participant to complete the 9-hole pegboard (Rolyan 9-hole peg test apparatus, plastic one-piece model (Patterson Medical)) and then remove all pegs^23^. During our clinical trials, the 9 HPT was repeated twice for each hand separately (D, dominant; ND, not dominant) with the writing hand considered to be the dominant one. The hand that was not involved in the test was rested on the participanatʼs lap. Figure 1e presents our setup where a participant is wearing the motion capture suit while performing the 9 HPT, and the blue 9-hole board is clearly shown on the table. Additionally, in Fig. 1e, we present a typical 9-HPT time-lapse (every frame shown is 0.5 s apart) from a single participant as collected by the motion capture suit.

### Feature generation

The full-body suit supplied us with 51 degrees of freedom (DoF) (3 DoF × 17 joints) joint angular data and 78 DoF (3 DoF × 26 segments) 3D position data of the body segments. The BVH files from all participants and visits were imported in Matlab R2015b and Matlab R2019b (The Mathworks) for analysis. A simple preprocessing has been applied to the data to transform the data into biomechanically meaningful values^52^. Using the joint angular and 3D body segment positions from the suit data of the 8-MW and 9-HPT subtasks, we extracted the features (F1–F18) listed in Table 2. A detailed description of the generation of these features is presented later in the methods. Both the 8-MW task and the 9-HPT task (for each hand) were repeated twice as per the SCAFI protocol. For each repeat of the task, we have a suit recording. Features were generated for each of the two suit recordings separately. So, for each visit of the participant, we have two sets of features for the 8-MW task and two sets of features for the 9-HPT task.

### Feature selection and model evaluation

We applied a GP regression algorithm to combine the extracted behavioral features and find a mapping against the clinical scales. We used a nested cross-validation procedure for feature selection and model evaluation (to avoid leakage of the test data during the feature selection process). The inner cross-validation loop was used for the feature selection and the outer cross-validation loop was used to evaluate the performance of the model. We used a leave-one-subject-out (leave the rows corresponding to all visits of a subject) for both the inner feature selection cross-validation loop and the outer model evaluation cross-validation loop. We used an exhaustive feature selection approach to select the most optimal subset of features.

For *s* number of participants with each *v* visits, the data consists of *s* × *v* rows and the outer cross-validation splits the data into *s* folds (ensuring all the visits of a participant are in a single fold and each fold contains only the rows corresponding to the visits of a single participant). So, we have *s* training and test folds. For example, for the cross-sectional predictions of the SARA scale using 8-MW suit features to predict the SARA scores of patient-1, all the visits of patient-1 were the test data and all the visits of the other eight FA patients and nine healthy controls were the training data. To predict the SARA scores of patient-2, all the visits of patient-2 were the test data and all the visits of the other eight FA patients and nine healthy controls were the training data, and so on. Feature selection of features was done for each of the *s* training folds using the leave-one-subject-out cross-validation error of the inner cross-validation loop as the objective function and *s* subsets of features were generated. The most frequent subset among the *s* subsets was selected as the optimal subset as the frequency of the subset of features is a measure of the robustness of the subset of selected features to changes in the training data. Finally, the overall performance of the GP regression was evaluated for the selected optimal subset of features using the outer cross-validation for the *s* test sets. This method ensured that an optimum subset of features is selected without any data leakage of the test set into the training set. This nested cross-validation approach ensured that the test data in each fold of the outer cross-validation loop was never used during feature selection in the inner cross-validation loop and therefore provides a reliable estimate of the model performance. The hyperparameters of the GP were chosen based on the cross-validation error on the inner nested loop. The predicted values from all the test folds of the outer fold were aggregated and the aggregate RMSE and the coefficient of determination (*R*^2^) were calculated and reported in the results section.

The cross-sectional predictions of the SARA and SCAFI scores were done for the FA patients and healthy controls. The longitudinal prediction of the clinical scales (SARA and SCAFI) using the scales themselves (for example, predicting SCAFI at T + 9 months using SCAFI at T + 0 months) was done on the clinical scales data from our study and a larger EFACTS study. This longitudinal prediction performance of the scales was then compared against the longitudinal prediction performance of the suit features from our study (for example, predicting SCAFI at T + 9 months using suit features from T + 0 months in our study cohort). The longitudinal prediction of the clinical scales using the scales themselves was done on a much larger cohort of EFACTS patients to increase the predictive power of the clinical scales. All our study participants have an onset of FA over 18 years of age (except one participant who had an age of onset of 15 years). We, therefore, included only participants from EFACTS cohort who had onset after 18 years. These EFACTS participants (Supplementary Table 1) were found to be matched for age, sex and age of onset with our study cohort. For the longitudinal results (Fig. 3c,f), which show the performance of the predictions as a function of the number of participants used to build the machine learning models, nCk (up to a maximum of 1,000) combinations (where *n* is the total number of participants in the dataset and *k* is the number of participants used for building the machine learning model) of the models were built for each *k* and the mean and standard deviation of the aggregate performance of the nCk models was reported.

### FXN measurement—RNA extraction and quantitative RT-PCR

Human blood samples were obtained from FA patients in accordance with UK Human Tissue Authority ethical guidelines. Peripheral blood mononuclear cells (PBMC) were isolated from the blood samples using a Ficoll-Hypaque TM gradient (Sigma) kit by following the manufacturerʼs protocol. Total RNA was isolated from the pelleted PBMC using Trizol (Invitrogen) and reverse transcribed using the ThermoScript TM Reverse Transcription system (Invitrogen) by following the manufacturerʼs instructions. Multiplexed qRT-PCR using TaqMan gene expression assays (ThermoFisher) targeting *FXN* (Assay ID = Hs00175940_m1) and TATA-box binding protein (TBP) (Assay ID = Hs00427621_m1) were performed in TaqMan Fast Advanced Master Mix (Applied Biosystems). The measured FXN mRNA levels were expressed relative to TBP as the endogenous control mRNA levels. The data presented are the normalized Ct values and therefore the higher the value the lower the level of *FXN* mRNA.

### GAA repeat tract analysis

Long-range PCR was performed with the Expand High Fidelity PCR system (Roche Diagnostics), using 200 ng input DNA per 50 µl reaction and GAA-B-F (5′-AATGGATTTCCTGGCAGGACGC-3′) and GAA-B-R (5′-GCATTGGGCGATCTTGGCTTAA-3′) primers^53,54^. The thermocycling conditions were 94 °C for 5 min; 10 cycles of 94 °C for 20 s, 61 °C for 30 s and 68 °C for 5 min; 20 cycles of 94 °C for 20 s, 62 °C for 30 s and 68 °C for 5 min with 20 s increments; and a final cycle of 68 °C for 10 min. The amplified PCR products contain the GAA repeat tract with flanking sequences of 157 bp at the 5′ end and 125 bp at the 3′ end. GAA repeat tracts were sized by separating on a 1% (wt/vol) SeaKem LE agarose TBE gel (Lonza). The number of GAA repeats was calculated from the size of the PCR product (*S*_PCR_, in bp) using the formula (*S*_PCR_ – 282)/3.

Triplet repeat (TP) PCR assays were used to examine interruptions at the 5′ and 3′ ends of the FXN GAA repeat tract independently^40^.

### Suit features

This section gives a description of the behavioral features that we extracted from the data of the 8-MW test and the 9 HPT. Features F1–F9 were extracted from the suit data of the 8-MW task and features F10–F18 were extracted from the suit data of the 9-HPT task.

### F1—workspace probability density volume and entropy

We measured the workspace volume, a concept that is extensively applied in biomechanics and rehabilitation applications for measuring the rigidity of body parts^55,56^. Workspace volume can be described as the volume generated by the movements of the limbs in space. The idea of workspace volume is illustrated in Extended Data Fig. 1a. Because the participants were not stationary in the 8-MW test, we adapted the concept of the workspace volume to make the computation more robust. We set the participantʼs trunk to a fixed reference point and adjusted the position of the other joints relative to the trunk. We then computed the occupancy density of the joints by separating the jointʼs 3D locations in space in a grid of 2 × 2 × 2 cm voxels. An example is shown in Extended Data Fig. 1a, where the color of each voxel represents the occupancy frequency on a log10 scale. Using the generated occupancy density of the joints, we computed the workspace volume by counting the nonempty voxels and multiplying the result by the volume of a single voxel (that is, 8 cm^3^). Applying this analysis across all participants, we observed that FA patients needed substantially more space (almost double) than the controls to perform the 8-MW task (Extended Data Fig. 1b, *P* < 0.001, Kruskal–Wallis one-way ANOVA).

To obtain a measure that is sensitive to the variability of workspace used, we defined the workspace entropy as negative log (probability) averaged over all nonempty voxels. Zero entropy implies no variability in the system: the body part occupies only a single voxel, while maximum entropy is achieved when a body part occupies all reachable voxels with equal probability, thus higher entropy implies more disorder/variability. We found that patients applied a wider range of joint configurations, which correlated with an increased entropy of the workspace density, as shown in Extended Data Fig. 1c, demonstrating a higher entropy in FA patients than controls (*P* < 0.001, Kruskal–Wallis one-way ANOVA). This suggested that not only was the FA patientsʼ workspace volume larger than controls, but their movements were also more variable, within their workspace volume. Thus, the walking patterns of FA patients were more disordered and less predictable, probably caused by the presence of compensatory mechanisms to balance their sensorimotor dysfunction during the walk.

### F2—lower body joint variability

We examined the variability of hip and knee velocities during the walk in the same way as standard gait analysis methods had been previously applied to Parkinsonʼs patients^57,58^. Using a step detection algorithm, we segmented the time-series data into walking cycles, afterward we Z-scored the joint angular velocities of hip and knee joints of each leg across all angular joint movement dimensions (flexion, abduction and rotation) to make a comparison across participants fairer. We calculated the average variability along each joint dimension. Patients exhibited a statistically higher variability across hip flexion and knee rotation (Kruskal–Wallis one-way ANOVA, where an asterisk represents *P* < 0.05, **P < 0.01 and ***P < 0.001), which supports our initial hypothesis that FA patientsʼ walk is more disordered.

### F3—walk autocorrelation and decay

While our measures up to this point focused on spatial features, as defined by the posture of the body, we next wanted to investigate the temporal structure. Autocorrelation is defined as the cross-correlation of a signal with itself at different points in time. We calculated the autocorrelation of the joint angular velocities of each one of the 51 DoF as collected by the suit and the result is shown in Extended Data Fig. 1d. While the 51 individual DoF exhibit some clear periodical patterns and some fewer periodical patterns, we wanted to analyze if there were underlying commonalities that were conserved. Because the autocorrelation was in a periodic setting, we converted the individual jointʼs autocorrelations across time into vectors: We discretized at 50-time points the autocorrelation function using bins of a width corresponding to 4% of a walk cycle. This produces a 50-dimensional vector covering two walk cycles (200%). We then performed Principal Component Analysis (PCA) on the collection of these 51 autocorrelation vectors and found that just three PCs can explain 80% of the variability in the autocorrelation signals across the 51 joints (Extended Data Fig. 1e). The first, second and third PCs are plotted in Extended Data Fig. 1f. These data-driven features of the walk cycle correspond to the various swings of legs, arms and body during walking. Comparing the PC value at the first walk cycle (Extended Data Fig. 1g), there is a significant difference between patients and controls on the first (*P* < 0.05) and second (*P* < 0.01) PCs but not on the third (*P* = 0.63, Kruskal–Wallis one-way ANOVA). This means that both leg and arm swings are more variable in duration than in controls. This suggests that the repeatability of body postures while walking is substantially reduced in FA patients in the temporal domain–they effectively accelerate and decelerate their swings more randomly than healthy controls.

### F4—channel delay cross-correlation

While the measures so far focused on spatial or temporal measures, we wanted to capture the correlation structure of the data across space and time. Therefore, we used the channel delay cross-correlation measure, which has been applied for extracting abnormalities in high dimensional neurological and motion capture data^59,60^. We applied the channel delay cross-correlation on our 8-MW dataset to capture the spatiotemporal correlation changes between all 51 joints of FA patients and controls. The main step for applying channel delay cross-correlation method is the construction of a 51 × 51 block matrix based on the joint angular velocities we get from the motion capture suit (51 DoF). The blocks along the main diagonal contain the within-channel autocorrelations and the off-diagonal blocks contain the cross-channel correlations. Within each block, the correlations are calculated for ten different lags (ten linearly spaced lags from zero up to the average walk cycle of the participant). Therefore, the channel delay cross-correlation matrix captures both spatial and temporal correlations of the joints during the walk. The matrix for one participant is shown in Extended Data Fig. 2a.

The next step is to calculate the matrix eigen spectrum, that is the array of eigenvalues, sorted from largest to smallest. The eigen spectrum encodes the magnitude of covariance in each (decorrelated) dimension and it is invariant to the ordering of the channel delay cross-correlation matrix columns and thus to relationships among particular time-delayed joints of the body. The eigen spectrums are reported in Extended Data Fig. 2b where the averaged eigen spectrum for patients and controls is shown in red and blue, respectively. The decreased power in the patient’s first few eigenvalues (that is, eigenvalues with indices 1–20) and the equivalent increase in power for the patientʼs remaining eigenvalues (that is, eigenvalues with indices 20–200) reflect the increased dynamical complexity in their walk when compared with controls. We speculate that this is a reflection of what clinicians subjectively characterize as the more ataxic gait of FA patients.

### F5—extremities velocity

The next feature we were interested to explore was the difference in the velocity profiles of subjectsʼ extremities as performed in standard gait analysis practices^61^. Using the extremitiesʼ 3D locations in space (wrists, ankles and head), as provided by the suitsʼ biomechanical model, we estimated the velocity on each body plane (sagittal, frontal and transverse) and then calculated the magnitude of the velocity by applying RMS operation. Using our step detection algorithm, we segmented the velocity signal at each walking step and then averaged the peak velocities observed at extremities across all voxels. The results for each extremity (separated in dominant (D) and nondominant (ND) side) are presented in Extended Data Fig. 2c where there is a statistical difference between FA patients and controls on the D and ND ankles and the ND wrist (*P* < 0.05, Kruskal–Wallis one-way ANOVA).

### F6—walk complexity

We have quantified the complexity of human walking using a dimensionality reduction algorithm PCA, where we observed the number of PCs required to successfully explain the variability in walking motion (higher number of PCs implies a more complex movement)^62,63^. We have applied the same analysis to our participantsʼ joint angular velocities and the results are presented in Extended Data Fig. 2d. It seems like the patientsʼ kinematics are a lot more complex because they require more PCs to explain the variability present (that is, to explain 80% of the motion variability in FA patients it requires at least five PCs, but only two PCs are needed for the controls). This observation is supported by the fact that ataxia patients are known to develop compensatory mechanisms to balance out the effects of the disease^64^.

To achieve quantification of the human movement complexity, we developed a metric based on the previous observations. For each participant, we calculated the area developed under the arc of the variability curve, divided that by the whole area of the upper left triangle and expressed the results as a percentage. We applied this analysis to all participants and the results are shown in Extended Data Fig. 2e, highlighting the statistically increased walk complexity of FA patients when compared to controls (*P* < 0.001, Kruskal–Wallis one-way ANOVA).

### F7—legs movement root mean square power spectrum

Our next feature is based on the analysis of the walking signature on the frequency domain. Spectral analysis of kinematic signals has been efficiently used for detecting abnormal gait patterns^65^. We low-pass filtered the angular velocities of hip flexion, hip abduction and knee flexion with a 10 Hz cut-off and then extracted a Short-Time Fourier Transform (STFT) based on a 200 ms window with 100 ms overlap. An example is shown in Extended Data Fig. 3a, where we calculated the STFT on the angular velocities from a participantʼs dominant (D) knee flexion and used different colors in the spectrogram (Extended Data Fig. 3b) to represent the power carried by a specific frequency of the signal at a specific moment in time. Afterward, we computed RMS on each window, which summarizes the information content of the power spectrum at each time point (Extended Data Fig. 3c). Finally, for each walk cycle, we calculated the area under the curve, which combines the total energy used by that joint. We applied the same analysis on the lower body joints of each leg and then averaged the energy presented across all walk cycles. The results are shown in Extended Data Fig. 3d where the FA patients present substantially lower energy in their D hip flexion, D knee flexion and ND knee flexion (Kruskal–Wallis one-way ANOVA) than controls. This finding is consistent with the muscle weakness in the arms and legs clinically reported to frequently occur in FA^10^.

### F8—joint velocities correlation coefficient

We additionally investigated the correlations between the movement of the joints of the lower body during the walk by estimating the Pearsonʼs product–moment correlation coefficients (*ρ*)^66^. Because FA patients exhibit a less standardized walking pattern, we would expect the correlations between various joints to be lower. Therefore, we used the angular velocities from the hip flexion and abduction and knee flexion joints and evaluated the correlation coefficients both across joints but also between D and ND leg. The results are shown in Extended Data Fig. 4, which compares the correlation coefficients of FA patients (blue) and controls (red) with most presenting a statistical difference between the two groups (Kruskal–Wallis one-way ANOVA, where an asterisk represents *P* < 0.05, **P < 0.01 and ***P < 0.001).

### F9—head-spine movement plane area

The last feature we extracted from the 8-MW data is based on the clinical observations that subjectively characterize FA patients as ‘wobblingʼ or sideways ’swayingʼ during the 8 m walk. The degree of sway has been suggested to be an early indication of the disease stage in ataxia^22^. To objectively quantify the ‘wobblingʼ walking effect, we used the following approach: we tracked the location of the head in a coordinate system that is stationary with respect to the axes of the hips. Thus, all motion in this coordinate system is seen relative to the hips and is hence always centered on the hips even during locomotion. Then, we calculated the location of the head marker with respect to the hip as shown in Extended Data Fig. 5a. The distance between the head and hips is relatively constant and so the head movements with respect to the hips are localized mainly in a 2-dimensional surface that is orthogonal to the line connecting the hipʼs center of mass with the head maker. During walking, the head markers move through this plane, and hence we calculated the area covered by the head movements generated during the walk of all subjects. Our results show a statistically higher area covered by the FA patients than the controls (Extended Data Fig. 5b; *P* < 0.001, Kruskal–Wallis one-way ANOVA), which confirms the qualitative observations ‘by eyeʼ during the clinical trial. Analyzing the variability of head movements independently for the frontal and sideways axis shows that there is a statistical difference between FA patients and controls for the forward movement (Extended Data Fig. 5c; *P* < 0.05; Kruskal–Wallis one-way ANOVA) and the sideways movement (Extended Data Fig. 5d; *P* < 0.001, Kruskal–Wallis one-way ANOVA).

### F10—average joint velocity

The first feature we extracted from the 9-HPT data is based on the average angular velocities of the shoulder and elbow joints. The FA disease causes progressive neurodegeneration, and this should result in slower joint velocities during the 9-HPT task. The results in Extended Data Fig. 6a support our hypothesis as the joint velocities of the FA patients are statistically lower than controls (*P* < 0.001, Kruskal–Wallis one-way ANOVA).

### F11—upper body complexity

Our next feature was also used in the 8-MW analysis, and it is based on a PCA of the joint angular velocities during the 9 HPT. In this case, although we only applied the analysis to the upper body joints, we also excluded the joints of the hand not performing the task. Extended Data Fig. 6b shows the variability explained by using different numbers of PCs. Observing the plot, we can see that FA patients require slightly more PCs to explain the variability in their movements than the controls. This result is consistent with our findings in the 8-MW task. We have additionally calculated our complexity metric in the same way as described in the 8-MW section and found that FA Patients have statistically more complexity in their movements than controls (Extended Data Fig. 6c; *P* < 0.001, Kruskal–Wallis one-way ANOVA).

### F12—workspace probability density volume and entropy

Similar to the workspace density analysis done for 8 MW, we first calculated the density plot for each subject (an example is shown in Extended Data Fig. 6d), which was then used to estimate the overall workspace volume. Because the task was performed twice per hand, we averaged the volume of each trial based on hand dominance. The results are shown in Extended Data Fig. 6e where the FA patients seem to have a significant difference between the volume generated by each hand (*P* = 0.003, paired *t* test), something that is not true for controls as they occupy roughly the same space when performing the task with either hand (*P* = 0.08, paired *t* test). Furthermore, comparing the performance between the two subject groups, the FA patients use a much larger workspace than controls when they perform the task with the D hand (*P* < 0.001, Kruskal–Wallis one-way ANOVA) and the ND hand (*P* < 0.001, Kruskal–Wallis one-way ANOVA). We additionally extracted the entropy of the subjectsʼ density plot as a measure of how ordered the space occupancy was. Based on the results in Extended Data Fig. 6f, FA patients have a lot more disorder across both hands than the controls during the 9 HPT.

### F13—upper body autocorrelation full width at half-maximum

The next feature we explored for the 9-HPT analysis was the jointsʼ autocorrelation full-width at half-maximum (FWHM), which is used as an indication of how rapidly the joint kinematics change^52^. We first calculated the autocorrelation up to a 10 s lag. Because the 9-HPT task is not a cyclic task, the output was a single bell-shaped curve centered around the 0 s lag. The FWHM is defined as the width of the bell-shaped curve at the point when it reaches a 0.5 autocorrelation value (half-maximum). We calculated the FWHM of all the joints of the arm performing the 9 HPT and the results are shown in Extended Data Fig. 7a. The FA patients have a substantially higher autocorrelation FWHM than controls in all jointsʼ dimensions except the shoulder elevation (Kruskal–Wallis one-way ANOVA), which indicates that patientsʼ movements are changing more slowly.

### F14—channel delay cross-correlation

Another feature that has been previously used in the 8-MW analysis and could potentially capture the differences between FA patients and controls in the 9 HPT is the channel delay cross-correlation. We estimated a 31 × 31 channel delay cross-correlation matrix based on the cross-correlations between the angular velocities of the upper body joints (31 DoF) within a window of 10 s with steps of 100 ms. We then calculated the eigen spectrum for each participant and the results averaged per participant group are shown in Extended Data Fig. 7b. In a similar fashion as the 8 MW, we observe a decreased power in the patientsʼ lower eigenvalues and an equivalent increase in power of the higher eigenvalues. This reflects an increased complexity in their FA patientsʼ upper body movements with respect to Controls.

### F15—arm root mean square power spectrum

We additionally extracted the average power from the upper body joints during the 9 HPT. This was achieved using the RMS power spectrum analysis explained earlier. However, because the task cannot be separated in cycles (like the 8 MW), we simply averaged the power intensity throughout the whole task. The results shown in Extended Data Fig. 7c reveal a substantially reduced power in the FA Patientsʼ joints except for the shoulder pronation (Kruskal–Wallis one-way ANOVA).

### F16—wrist average velocity

We also looked into the average velocity of the participantsʼ wrist during the 9 HPT. Using the suit biomechanical model, we calculated the 3D position of the wrist in space (only for the hand performing the task), we estimated the velocity on each plane (sagittal, frontal and transverse) and then estimated the velocity magnitude by applying rms operation. The participantsʼ average velocity separated by hand dominance is shown in Extended Data Fig. 7d, where FA patients have a substantially reduced speed in both hands compared to controls (Kruskal–Wallis one-way ANOVA) but they also have a slower speed in the ND hand than the D (*P* < 0.05, paired *t* test), something that is not true for controls as both hands can perform the task with the same average speed (*P* = 0.41, paired *t* test).

### F17—logistic fit on jointsʼ angular velocity

An additional feature we extracted that can successfully capture the differences between the FA patients and controls is the variability of jointsʼ angular velocities during the 9 HPT. We first compared the distributions of the joint velocities between FA patients and controls. We observed that the controls consistently have a much wider distribution across joints (see Extended Data Fig. 8a for an example), meaning they were applying much faster movements. To examine these differences in a more principled manner, we fitted multiple parametric probability distributions (Extended Data Fig. 8b) on the velocitiesʼ probability density function of each joint and examined the parameters of the distribution that best fits the data that is, the one that minimizes the Akaike Information Criterion. From our analysis, we have consistently found that the participantsʼ joint velocities are best described by a logistic probability distribution. So, we fitted a logistic distribution on all jointsʼ velocities and compared the scale parameter (σ) of the logistic distribution between FA patients and controls. The FA patients exhibit a substantially lower σ than the controls (Extended Data Fig. 8c) for most joints indicating that they apply a smaller range of velocities during the 9 HPT.

### F18—head-spine movement

Finally, as performed with the 8-MW analysis, we have explored the head movements as an indication of how well subjects can balance their body. Comparing the area generated by the FA patientsʼ heads during the 9 HPT, we have found that it is substantially increased when compared to the controls (*P* < 0.001, Kruskal–Wallis one-way ANOVA).

### Reporting summary

Further information on research design is available in the Nature Portfolio Reporting Summary linked to this article.

## Online content

Any methods, additional references, Nature Portfolio reporting summaries, source data, extended data, supplementary information, acknowledgements, peer review information; details of author contributions and competing interests; and statements of data and code availability are available at 10.1038/s41591-022-02159-6.

## Supplementary information

Supplementary Information Supplementary Tables 1–3, Supplementary Figs. 1–8 and Supplementary Note.

Reporting Summary

## Data Availability

The data used in the study are not publicly available due to them containing information that could compromise research participant privacy/consent. Anonymized data for academic purposes can be made available upon request via email to the corresponding author.

## Code availability

The machine learning code that supports the findings of this study is available for academic purposes at 10.6084/m9.figshare.20440449
