# Supplementary material for: A wearable motion capture suit and machine learning predict disease progression in Friedreich’s ataxia
Source: Nat Med. 2023 Jan 19;29(1):86–94. doi: 10.1038/s41591-022-02159-6 (PMC9873563; doi:10.1038/s41591-022-02159-6)
Supplement: Supplementary file 1 — Supplementary Tables 1–3, Supplementary Figs. 1–8 and Supplementary Note. [file 41591_2022_2159_MOESM1_ESM.pdf]

nature medicine

Article https://doi.org/10.1038/s41591-022-02159-6

A wearable motion capture suit and machine learning predict disease progression in Friedreich's ataxia

In the format provided by the authors and unedited

Supplementary Table 1. Characteristics of the EFACTS study participants included in our analysis. Data is presented as mean (range) or N(%).

N	164
Women<br>N (%)	91<br>(55.5%)
Non-ambulatory<br>N (%)	31<br>(19%)
Age at study entry (in years)	46.34<br>(22 - 76)
Age of onset (in years)	29.26<br>(18 - 65)
Disease duration (in years)	17.08<br>(2 - 40)
SARA	16.81<br>(1.50-34.00)
SCAFI	-0.05<br>(-2.46-2.54)
8MW<br>(in seconds)	11.24<br>(3.25-99.00)
9HPT – Dom. Hand<br>(in seconds)	50.67<br>(11.50-289.00)
9HPT – Non-Dom. Hand<br>(in seconds)	56.35<br>(16.30-248.00)
PATA	21.08<br>(7.00-39.00)
GAA short allele repeats	352<br>(43 - 1000)
GAA long allele repeats	787<br>(150 - 1250)

Supplementary Table 2. List of suit features from the 8MW task along with their mean and standard error values for the FA patients and control cohorts and p-value of the Kruskal-Wallis one-way ANOVA test between the cohorts. Data are presented as mean (standard deviation). Please note that there are 2 measurements per visit.

Feature name	FA patients mean(std) (N=62 measurements)	Controls mean(std) (N=18 measurements)	p-value
Workspace volume	12234.84 (1634.98)	7511.11 (1876.85)	5.69E-10
Workspace entropy	0.19 (0.04)	0.1 (0.03)	1.26E-09
Variability in velocity - hip flexion	0.13 (0.04)	0.19 (0.25)	0.044987291
Variability in velocity - knee rotation	0.5 (0.13)	0.42 (0.24)	0.048814512
Principal component 1 of autocorrelation at first walk cycle	2.06 (0.78)	2.47 (1.57)	0.019340498
Principal component 2 of autocorrelation at first walk cycle	-0.68 (0.32)	-0.39 (0.45)	0.00511391
1st eigen spectrum values of the channel-delay cross-correlation matrix	349.09 (66.48)	432.28 (136.82)	0.024657373
5th eigen spectrum values of the channel-delay cross-correlation matrix	112.88 (29.36)	183.36 (69.24)	5.95E-06
35th eigen spectrum values of the channel-delay cross-correlation matrix	17.96 (3.76)	13.5 (4.37)	3.90E-05
Average peak velocity - dominant ankle	111.27 (35.92)	157.5 (27.37)	6.44E-07
Average peak velocity - non-dominant ankle	109.34 (28.91)	149.59 (18.57)	5.39E-07
Average energy per walk cycle of dominant hip flexion velocity	121.07 (31.58)	149.95 (25.66)	0.000648632
Average energy per walk cycle of dominant hip abduction velocity	50.6 (16.01)	59.65 (15.63)	0.028588101
Average energy per walk cycle of dominant knee flexion velocity	211.66 (64.02)	284.34 (53.26)	1.40E-05
Average energy per walk cycle of non-dominant hip flexion velocity	132.77 (57.35)	160.15 (29.48)	0.001067297
Average energy per walk cycle of non-dominant hip abduction velocity	56.63 (27.44)	62.28 (16.69)	0.041416272
Average energy per walk cycle of non-dominant knee flexion velocity	221.31 (89.05)	292.89 (53.72)	3.90E-05
Number of Principal components to explain 90% variance	7.49 (2.45)	5.8 (2.65)	0.016552015
Walk complexity metric	12.77 (3.32)	10.44 (3.69)	0.012014184
Correlation between non-dominant hip abduction and non-dominant hip flexion	0.12 (0.48)	-0.15 (0.47)	0.038088284
Correlation between non-dominant knee flexion and non-dominant hip flexion	-0.49 (0.11)	0.02 (0.28)	2.57E-09

Correlation between dominant hip flexion and non-dominant hip flexion	-0.45 (0.1)	-0.61 (0.08)	9.58E-08
Correlation between dominant hip rotation and non-dominant hip flexion	0.2 (0.15)	0.09 (0.13)	0.002095939
Correlation between non-dominant knee flexion and non-dominant hip abduction	-0.35 (0.43)	-0.02 (0.26)	0.005490941
Correlation between dominant hip flexion and non-dominant hip abduction	-0.14 (0.28)	0.16 (0.34)	0.001793972
Correlation between dominant hip flexion and non-dominant knee flexion	0.21 (0.16)	0.09 (0.19)	0.012014184
Correlation between dominant knee flexion and non-dominant knee flexion	-0.14 (0.18)	0.27 (0.12)	1.44E-08
Correlation between dominant knee flexion and dominant hip flexion	-0.57 (0.1)	0.06 (0.33)	3.18E-09
Area of head movement on the transverse plane	170.06 (77.29)	39.38 (22.44)	5.69E-10
Variance of head movement in the frontal axis	16.81 (8)	11.87 (8.07)	0.03917026
Variance of head movement in the sideways axis	12.88 (10.12)	2.92 (2.25)	5.38E-08

Supplementary Table 3. List of suit features from the 9HP task along with their mean and standard error values for the FA patients and control cohorts and p-value of the Kruskal-Wallis one-way ANOVA test between the cohorts. Data are presented as mean (standard deviation). Please note that there are 2 measurements per visit.

Feature name	FA patients mean(std) (N=62 measurements)	Controls mean(std) (N=18 measurements)	p-value
Average velocity - Shoulder direction	13.87 (6.32)	20.36 (8.7)	1.17E-05
Average velocity - Shoulder elevation	6.77 (2.58)	17.22 (9.07)	1.39E-15
Average velocity - Shoulder rotation	12.75 (4.85)	27.92 (8.73)	7.81E-17
Average velocity - Elbow flexion	14.93 (6.52)	44.67 (32.16)	8.68E-16
Average velocity - Elbow pronation	14.62 (8.35)	44.94 (38.09)	4.40E-10
Workspace volume	2625.94 (1252.23)	1423.11 (330.83)	2.39E-10
Workspace entropy	0.17 (0.04)	0.11 (0.02)	1.48E-15
Autocorrelation FWHM - shoulder direction	293.53 (85.75)	166 (61.54)	3.75E-12
Autocorrelation FWHM - shoulder elevation	305.15 (111.99)	227.03 (40.9)	7.73E-06
Autocorrelation FWHM - shoulder rotation	298.33 (93.8)	190.65 (55.11)	4.74E-10
Autocorrelation FWHM - elbow flexion	238.07 (66.09)	137.69 (36.75)	7.40E-15

Autocorrelation FWHM - elbow pronation	195.23 (72.24)	121.71 (35.6)	5.57E-09
1st eigen spectrum values of the channel-delay cross-correlation matrix	32.19 (9.86)	42.42 (13.89)	2.08E-05
5th eigen spectrum values of the channel-delay cross-correlation matrix	22.66 (5.71)	33.17 (10.84)	1.39E-08
30th eigen spectrum values of the channel-delay cross-correlation matrix	6.44 (0.65)	8.23 (1.13)	3.33E-15
300th eigen spectrum values of the channel-delay cross-correlation matrix	0.35 (0.11)	0.05 (0.06)	2.72E-19
Average velocity - wrist	6.95 (1.93)	10.87 (3.05)	6.46E-11
Average power - shoulder direction	54.45 (26.18)	82.11 (35.7)	1.81E-06
Average power - shoulder elevation	26.67 (10.16)	68.56 (37.92)	1.96E-15
Average power - shoulder rotation	50.71 (21.69)	114.02 (37.38)	6.42E-17
Average power - elbow flexion	59.49 (26.96)	187.25 (152.96)	1.23E-15
Average power - elbow pronation	58.95 (34.41)	195.46 (203.34)	3.70E-10
Area of head movement on the transverse plane	53.81 (47.62)	8.69 (14.02)	1.36E-14
Variability of wrist velocity	1.52 (0.55)	2.38 (0.66)	5.36E-10
Number of Principal components to explain 90% variance	7.29 (2.55)	4.43 (2)	6.05E-09
Movement complexity metric	12.27 (3.1)	8.3 (2.68)	2.75E-10
Variability of shoulder direction	10.36 (4.75)	15.12 (6.54)	1.66E-05
Variability of shoulder elevation	4.99 (1.9)	12.71 (6.75)	1.63E-15
Variability of shoulder flexion	9.47 (3.6)	20.78 (6.53)	6.85E-17
Variability of elbow flexion	11.03 (4.87)	33.02 (24.62)	8.96E-16
Variability of elbow pronation	10.72 (6.19)	33.17 (29.26)	6.07E-10
Variability of neck flexion	1.56 (0.63)	2.44 (1.46)	0.000158969
Variability of neck abduction	1.59 (0.64)	2.33 (0.75)	4.83E-07

Supplementary Figures

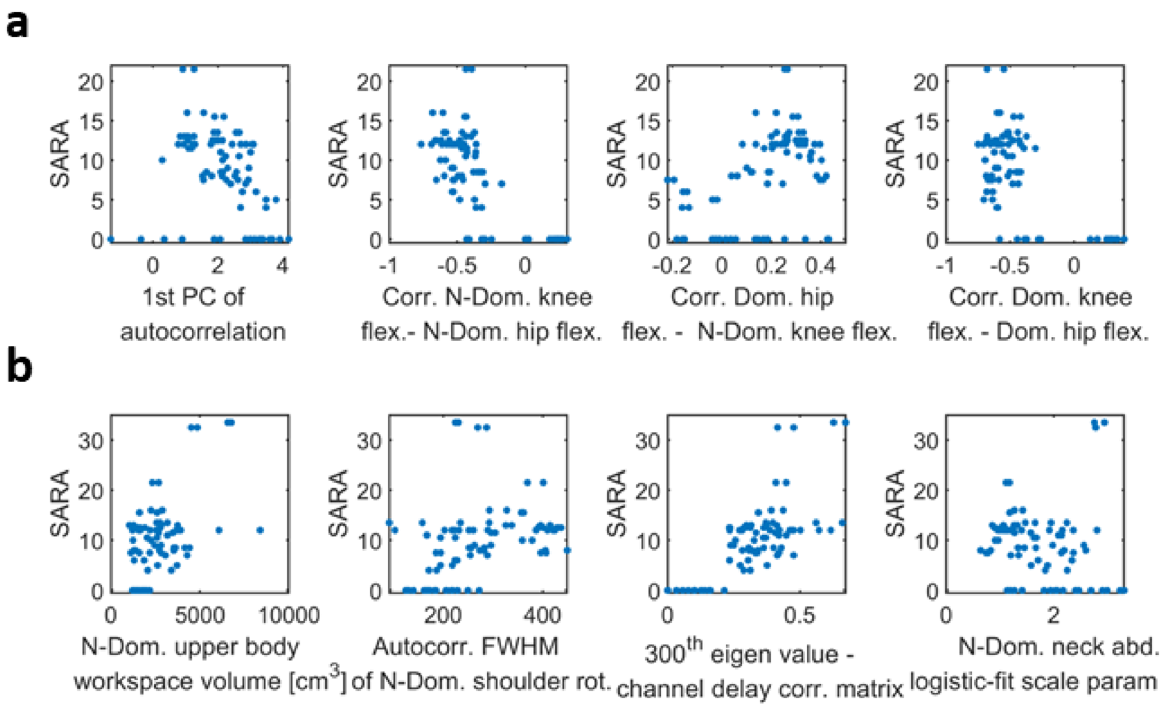

Supplementary Fig. 1 | Suit features selected by feature selection algorithm for cross sectional predictions of SARA. Scatter plot of the selected suit features of 8MW **(a)** and 9HPT **(b)** against SARA.

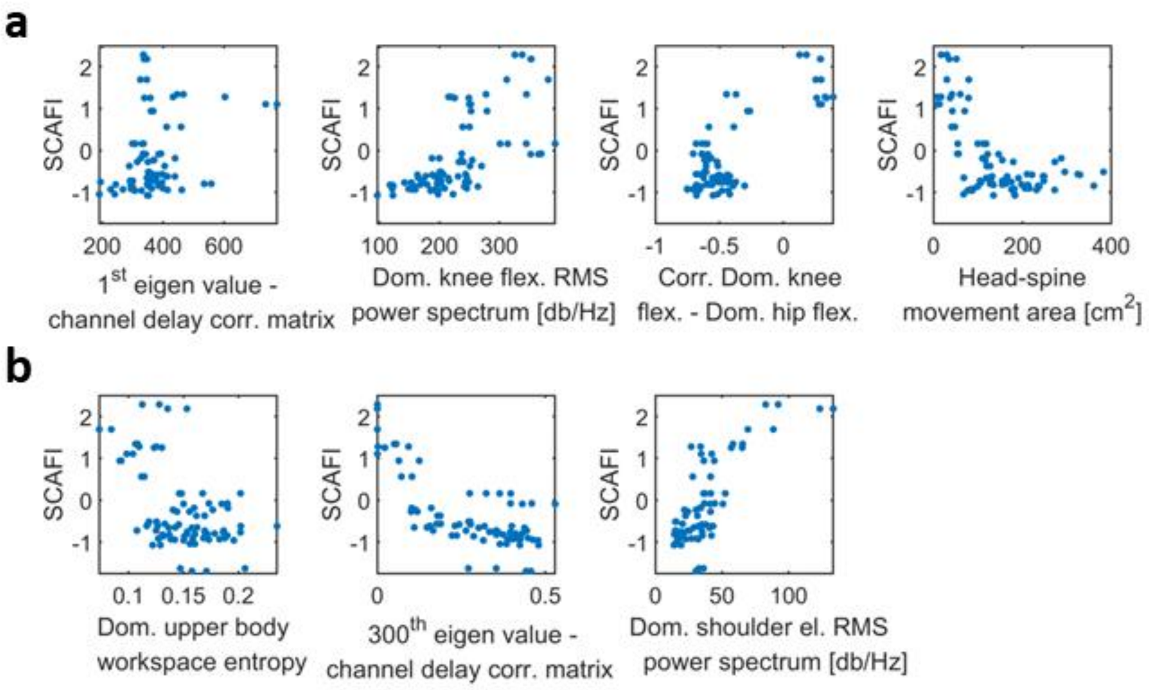

Supplementary Fig. 2 | Suit features selected by feature selection algorithm for cross sectional predictions of SCAFI. Scatter plot of the selected suit features of 8MW **(a)** and 9HPT **(b)** against SCAFI.

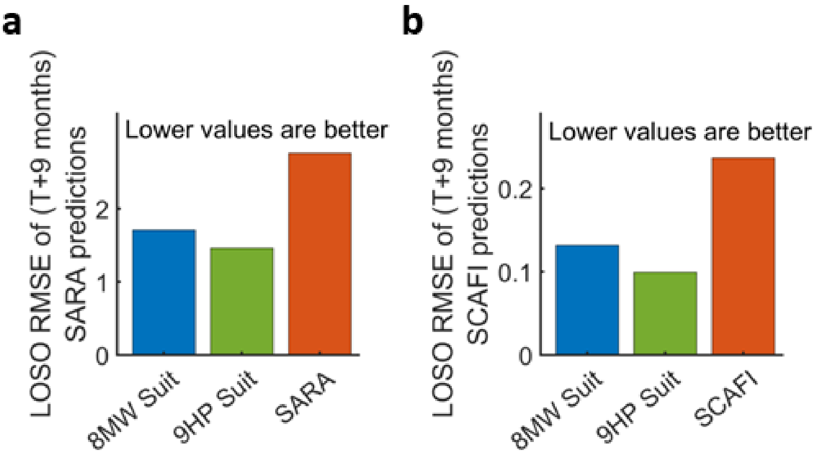

Supplementary Fig. 3 | Longitudinal predictions of SARA and SCAFI | a. Comparison of the aggregate RMSE of the leave-one-subject-out cross-validated predictions of the SARA at T+ 9 months by the suit features of 8MW and 9HPT tasks and SARA scale from visit T of our study. Subplot **b** is the corresponding plot for SCAFI.

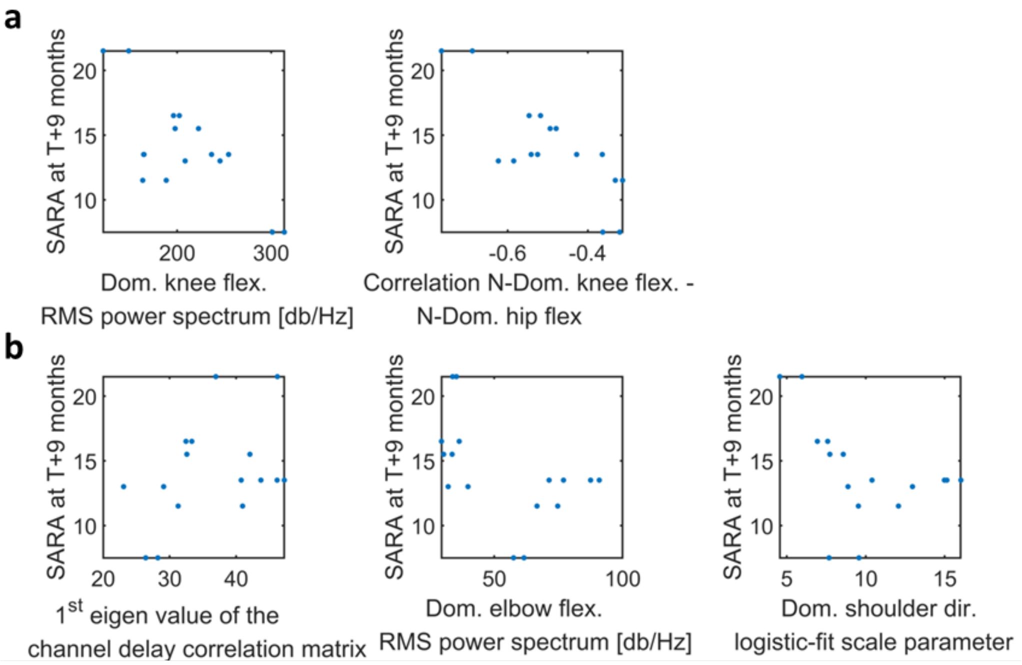

Supplementary Fig. 4 | Fingerprints selected by feature selection algorithm for longitudinal predictions of SARA. Scatter plot of the selected suit features of 8MW **(a)** and 9HPT **(b)** at time T against SARA at visit T+9 months.

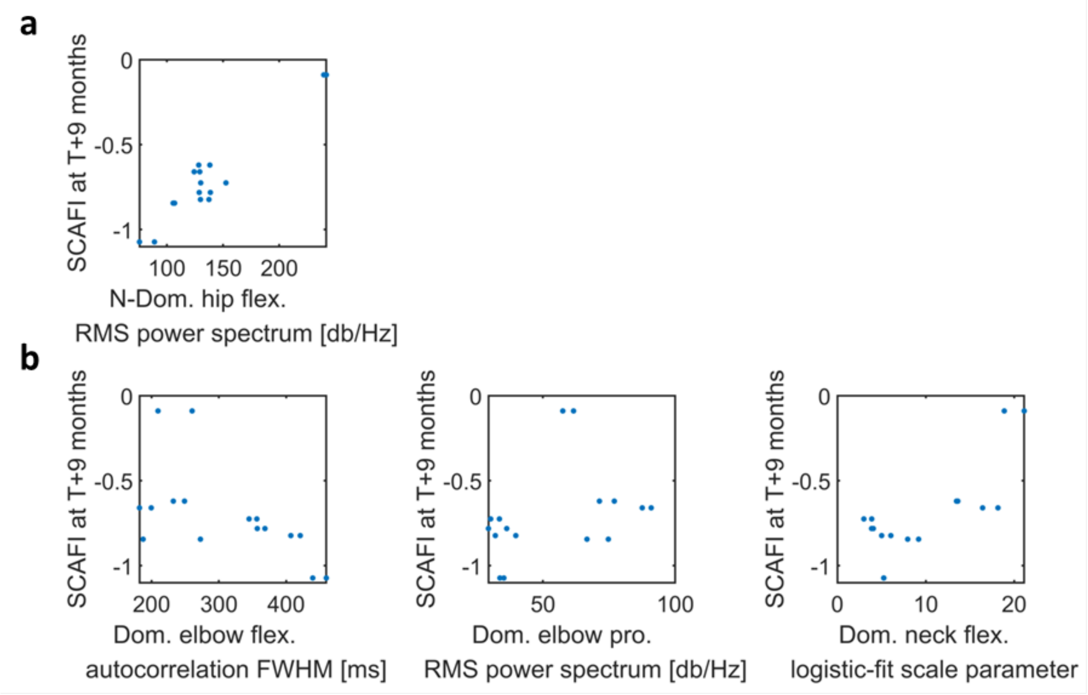

Supplementary Fig. 5 | Fingerprints selected by feature selection algorithm for longitudinal predictions of SCAFI. Scatter plot of the selected suit features of 8MW **(a)** and 9HPT **(b)** at time T against SCAFI at visit T+9 months.

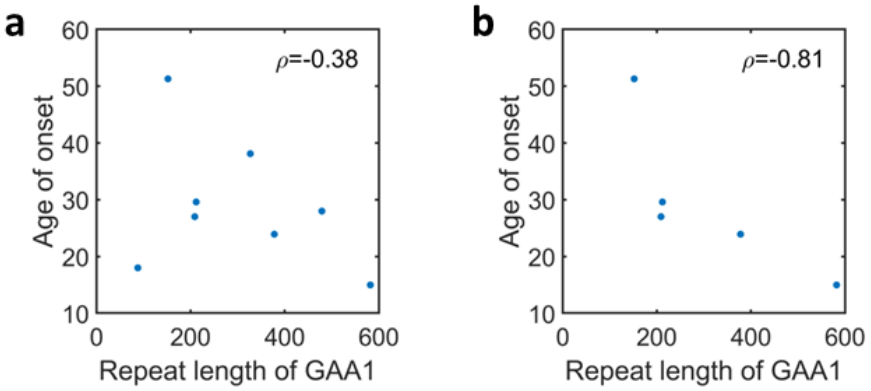

Supplementary Fig. 6 | Plot of short GAA length against the age of onset: a. Plot of the baseline repeat length of GAA short allele against the age of onset including patients with 3′ interruptions. **b.** Plot of the baseline repeat length of GAA short allele against the age of onset excluding patients with 3′ interruptions. There is a good correlation between short GAA repeat lengths and age of onset when the repeats with interruptions are excluded.

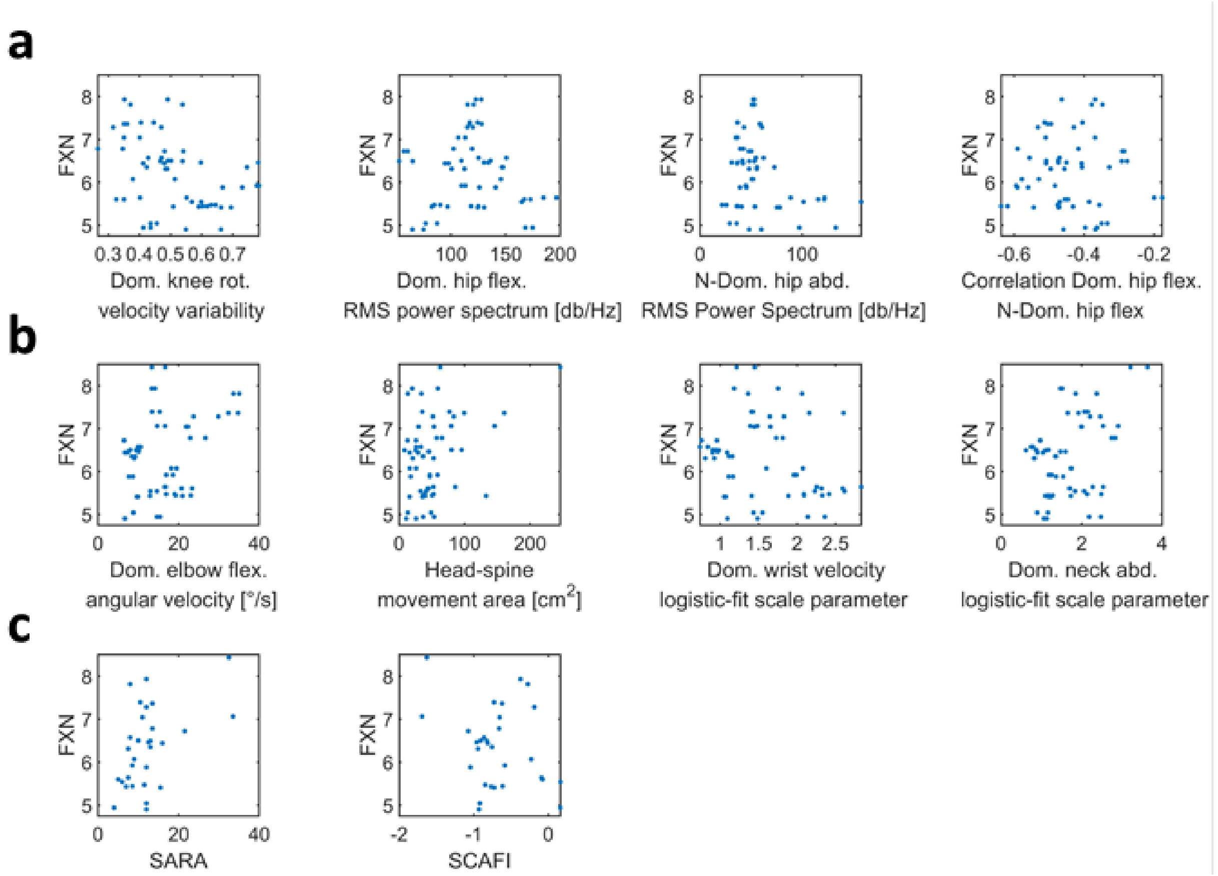

Supplementary Fig. 7 | Suit features selected by feature selection algorithm for predictions of *FXN*. Scatter plot of the selected suit features of 8MW **(a)** and 9HPT **(b)** against *FXN*. **(c)** Scatter plot of the *FXN* against SARA and SCAFI. *FXN* mRNA was measured by Q-RTPCR and the CT values normalised to TBP are plotted.

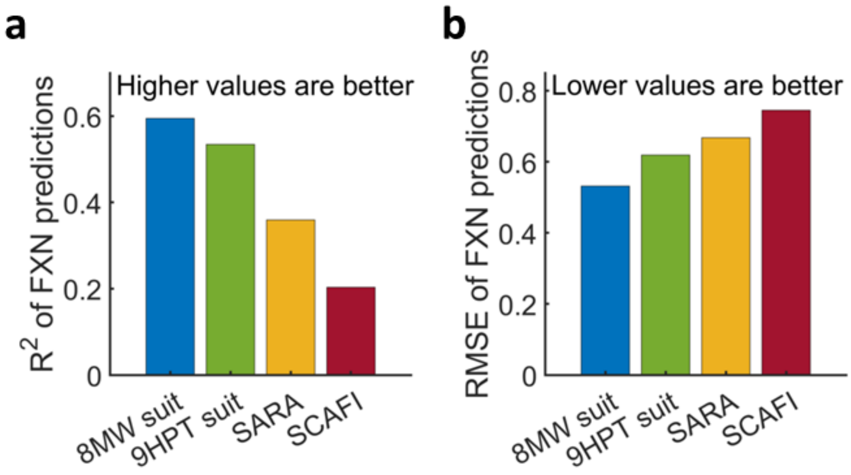

Supplementary Fig. 8 | Cross sectional predictions of *FXN* gene expression. *FXN* mRNA levels were predicted using the following 4 sets of predictors: suit features from the 8MW task, suit features from the 9HPT task, individual components of SARA score and individual time components of SCAFI scores. **a & b**. Leave-one-subject-out cross-validated R^2 and RMSE of the predictions by the 4 set of predictors. GP regression models using the features of the suit data of 8MW and 9HPT tasks as predictors perform better vis-a-vis GP regression models using the individual components of the SARA and SCAFI scales as predictors.

Imperial College London

Supplementary Note – Study Protocol

Pharmacodynamic Studies of a Histone Deacetylase Inhibitor in Friedreich's ataxia
(**Short title: The effect of Nicotinamide (Vitamin B3) in Friedreich's ataxia / Nicotinamide Study**)

**Substance:**	Nicotinamide
**Protocol Number:**	CR01849
**EudraCT No:**	2011-002744-27
**REC REF No:**	11/LO/0998

Chief Investigator:
Professor Richard Festenstein, MB BS PhD FRCP Imperial College London, Hammersmith Hospital, United Kingdom (UK)

Co-Investigators:
Dr Vincenzo Libri [National Institute of Health Research/Wellcome Trust Imperial Clinical Research Facility, (Imperial CRF) / UCL]
Dr Paola Giunti (National Hospital for Neurology and Neurosurgery)
Dr Aldo Faisal (Part IV, Imperial College London)

Sponsor: Imperial College London

Clinic:
NIHR/ Wellcome Trust Imperial Clinical Research Facility (Imperial CRF)
Imperial Centre for Translational and Experimental Medicine
Hammersmith Hospital
DuCane Road
W12 0HS
Phone: 020 331 38070
Fax: 020 331 31763

The study will be conducted according to the principles of Good Clinical Practice (GCP) in the United Kingdom (UK).

CHIEF INVESTIGATOR

I have read the foregoing protocol and agree to conduct the study as outlined. I agree to conduct the study in compliance with all applicable regulations and guidelines as stated in the protocol. I will ensure that all safety data will be collected, processed, and reported in accordance with local SOPs and regulations, and that information about all serious adverse events will be transmitted according to timelines described in the protocol.		
**Hammersmith Hospital, Imperial College Healthcare NHS Trust**	Professor Richard Festenstein, MB BS PhD FRCP<br>Experimental Physiology<br>Imperial College London<br>Hammersmith Hospital<br>Du Cane Road<br>United Kingdom<br>Phone: +44-208-383-8310<br>Fax: +44-208-383-8306	Signature<br>Richard Festenstein

IMP, SAE NOTIFICATION

**Trial Medication**	Nicotinamide	
**Notification of Serious Adverse Events**	Professor Richard Festenstein MB BS PhD FRCP	Within UK<br>Phone: 0208-383-8310<br>Fax: 0208-383-8306

CLINIC PERSONNEL

**Imperial CRF**	Professor Richard Festenstein, PhD FRCP MB BS<br>Dr Vincenzo Libri MD<br>Dr Naomi Loyse, Clinical Projects Manager, PhD<br>Dr Les Huson, Statistician

FUNDING:

**Funding**	This study is funded by Ataxia UK, AISA, Ataxia Ireland, ACHAF and the Imperial Biomedical Research Centre.

NIHR CLINICAL RESEARCH NETWORK ADOPTION:

**NIHR CRN Support**	This study is supported by North Thames Dementias and Neurodegenerative Diseases Research Network (DeNDRoN) which is part of the NIHR Clinical Research Network

1. LIST OF ABBREVIATIONS AND TERMS 9
2. BACKGROUND 12
2.1. What previous work is this project based on? 12
2.2. Clinical Experience with Nicotinamide 14
2.3. Why is a study needed now? 14
2.4. Outcomes and impact 14
2.5. How will the results of the study be taken forward? 14
3. STUDY OBJECTIVES 15
3.1. Primary Objective 15
3.2. Secondary objectives 15
4. STUDY DESIGN 15
4.1. Overall Study Design and Plan Description 15
4.2. Discussion of Study Design 19
4.3. Selection of Study Population 20
4.4. Inclusion Criteria for the interventional study 20
4.5. Exclusion Criteria for the interventional study 21
4.6. Inclusion Criteria for the non-interventional study (Part IV) 21
4.7. Exclusion Criteria for the non-interventional study (Part IV) 21
4.8. Participant Completion 21
4.9. Withdrawal of Participants from Study 22
4.10. Prior and Concomitant Therapy 23
4.11. Dose Reduction Guidelines for Potential Side Effects 23
5. TREATMENTS 23
5.1. Study Product, Dose, and Route of Administration 23
5.2. Metabolism 23
5.3. Pharmacokinetics 23
5.4. Rationale for Nicotinamide Dose Selection 24
5.5. Study Product 24
5.6. Storage 24
5.7. IMP Labelling 24
6. EFFICACY AND SAFETY VARIABLES 24
6.1. Safety Variables 24
6.2. Efficacy variables 25
7. OBSERVATION AND METHODS 25
7.1. Physical Examination and Medical History 25
7.2. Body sensor devices (Motion capture device / Body suit; Part IV only) 25
7.3. Eye movement tasks (eyetracking; Part IV only - optional) 26
7.4. Balance control tasks (using a force plate) 26

7.5. Laboratory Assessments ... 29
7.6. Volume of Blood Collection ... 29
7.7. Prior and Concomitant Medications ... 31
7.8. Screening and Baseline ... 31
7.9. Single Dose Escalation Visits ... 32
7.10. Multiple dose study ... 32
7.11. End of Interventional Study Follow-up Visit ... 34
7.12. Part IV ... 35

8. SAFETY ... 36

8.1. Reference Safety Information for nicotinamide ... 36
8.2. Adverse Event Terminology / Definition ... 37
8.3. Adverse Drug Reaction ... 38
8.4. Serious Adverse Event/Serious Adverse Drug Reaction ... 38
8.5. Assigning Severity to an Adverse Event ... 38
8.6. Assigning Causality of the Adverse Event to the IMP ... 39
8.7. Adverse Event Recording and Reporting ... 40

9. DOCUMENTATION OF DATA ... 41

10. EVALUATION AND STATISTICS ... 42

11. ADMINISTRATIVE AND LEGAL CONSIDERATIONS ... 42

11.1. Pre-Trial Requirements ... 42
11.2. General Legal Requirements ... 42
11.3. Protection of Participants ... 42
11.4. Amendments ... 43
11.5. Premature Termination of the Trial ... 43
11.6. Sponsor's / Chief Investigator Responsibilities ... 43
11.7. Record Retention ... 44
11.8. Confidentiality ... 44
11.9. Study Monitoring ... 45
11.10. End of Clinical Trial Report ... 45
11.11. Publications ... 45
11.12. Audits and Inspections ... 45

REFERENCES ... 46

12. SUMMARY TIMELINES ... 47

12.1. Interventional Study (single and multiple dose, Parts I, II & III) ... 47
12.2. Non-Interventional Study (Part IV) for FRDA participants ... 51
12.3. Non-Interventional Study (Part IV) for healthy volunteers ... 51

13. APPENDICES ... 52

13.1. Appendix 1: SARA scale ... 52

13.2. Appendix 2: SCAFI 54
13.3. Appendix 3: FARS SCALE PART II / Activities of Daily Living 56
13.4. Appendix 4: Edinburgh handedness score 58
13.5. Appendix 5: Possible scenarios for the motion capture suit task 59
13.6. Appendix 6: Balance Control Tasks 60

SYNOPSIS

**Title of study:** Pharmacodynamic studies of a histone deacetylase inhibitor in Friedreich's ataxia<br>Short title: Nicotinamide Study	
**Chief Investigator:**<br>Richard Festenstein (Imperial College London)<br>**Co-Investigators:**<br>Dr Vincenzo Libri (Imperial CRF, Imperial College London / University College London)<br>Dr Paola Giunti (National Hospital for Neurology and Neurosurgery, University College London)<br>Dr Aldo Faisal (Part IV, Imperial College London)<br>**Clinical Projects Manager:**<br>Dr Naomi Loyse (Imperial CRF, Imperial College London)	
**Study centres:**<br>Imperial CRF, Hammersmith Hospital, UK;<br>Imperial College Centre Imaging Facility (CIF), UK (Part IV only)	
**Study period:**<br>1 year	**Phase of development:**<br>Phase IIa – Proof-of-concept study to determine the dose of nicotinamide which may be effective in upregulating Frataxin (FXN) levels in Friedreich's ataxia (FRDA) participants. We will also assess the long-term clinical effects on FRDA participants of taking nicotinamide daily over a two month period.
9-12 months	In Part IV, we will investigate the use of novel, highly-sensitive technology to capture clinical deficit and reliably measure subtle changes in the activities of daily living (ADL). We will also improve the accuracy and specificity of FXN protein measurement, identify informative biomarkers that might track FXN levels and analyse the spatial organisation of the FXN locus. This part of the study will be conducted over a 9-12 month period without nicotinamide.

Methodology: This is a proof-of-concept study to determine the dose of nicotinamide which may be effective in up-regulating FXN levels in FRDA participants. We will also assess the long term clinical effects in FRDA participants of taking nicotinamide daily over the course of two months.
Part IV of the study will investigate the use of novel, highly-sensitive technology [functional Magnetic Resonance Imaging (fMRI) and motion capture devices, eye tracking (optional) and balance control tasks (optional) to capture clinical deficit and reliably measure subtle changes in the ADL over a 9-12 month period without nicotinamide. Functional changes will be correlated to levels of expression of FXN protein and the epigenetic structure of the FXN gene.

Clinical measurements

Laboratory measurements: The study will employ quantitative Real Time Polymerase Chain Reaction (Q-RT-PCR) to measure FXN transcript levels and estimate FXN protein levels using the Mitosciences dipstick reader following acute dosing with nicotinamide. Western blotting and mass spectrometry may be employed for further verification of the FXN protein levels and Ribonucleic acid-Sequencing (RNAseq) for the FXN messenger RNA (mRNA) and biomarker levels.
In Part IV, the study will investigate the use of a novel targeted mass spectrometric assay (Liquid chromatography-tandem mass spectrometry, LC-MS/MS) to improve the accuracy and specificity of FXN protein measurement, identify informative biomarkers that might track FXN levels using RNAseq and analyse the spatial organisation of the FXN locus by Chromosome-Conformation Capture coupled with high-throughput sequencing (3C-sequencing).

Number of participants:
A minimum of ten up to a maximum of 20 FRDA participants will participate in each part of the study.

A maximum of 20 healthy volunteers (HVs) will participate in Part IV only.

Diagnosis and main criteria for inclusion and exclusion for FRDA participants:
Inclusion Criteria:
FRDA participants over the age of 18 years, living in the UK and registered with a GP.
A confirmed molecular genetic diagnosis of FRDA, demonstrating GAA repeat expansions on both alleles.
Exclusion criteria:
Participants with significant clinical dysphagia
Participants with any serious intercurrent abnormalities or illness or history of liver disease.
Participants with fMRI contraindications (Part IV only).

Main criteria for inclusion and exclusion for healthy volunteers (Part IV)
Inclusion Criteria:
Participants over the age of 18 years.
Exclusion criteria:
Participants with fMRI contraindications.

Test product and mode of administration (Parts I, II and III): Nicotinamide, oral.

Duration of treatment:
Ten visits for the single dose escalation (Part I).
Participants will be admitted for four nights for Part II of the multiple dose study.
If participants tolerate the multiple dose dosing regimen and changes in FXN level are observed, participants will continue to take nicotinamide daily and come in for weekly visits over a two month period (Part III of the multiple dose study).
Eight visits (or 4 overnight stays) for Part IV for FRDA participants.

Several visits for the HVs (Part IV).

Comparator therapy, dose and mode of administration:
No comparator therapy will be used in this study (pre-post therapy study design).

Criteria for evaluation:
The primary outcome:
The primary endpoint in this study is significant upregulation of FXN in participants with FRDA. The pathological repression of FXN in FRDA is reported to be approximately 25% of normal. Ideally upregulation above this threshold would be predicted to be beneficial on long-term treatment. Theoretically a more modest increase in levels could also be of benefit. This study will determine whether nicotinamide can upregulate FXN levels in participants providing a potential radical treatment for this currently incurable and devastating disease.

Secondary outcomes:
Further assessment of efficacy by means of:
Chromatin immunoprecipitation to look for epigenetic changes at the FXN locus compatible with transcriptional upregulation. Such information might also be useful in identifying participants more likely to respond to this therapy by up-regulating FXN. These data will maximize the information gained from drug exposure and facilitate interpretation of the primary measure by providing proof-of-mechanism and biological and physiological plausibility.
Assessment of the long-term clinical effects in FRDA participants taking nicotinamide daily over the course of two months.
Assessment of additional FRDA biomarkers using gene expression profiling.

In part IV, we will:
Investigate the use of novel, highly-sensitive technology to capture clinical deficit and measure subtle changes in the activities of daily living over a 9-12 month period without nicotinamide.
Correlate functional changes to levels of expression of FXN protein and the epigenetic structure of the FXN gene.

Analysis plan and Statistical Methods:
The interventional study (Parts I, II and III) is a proof-of-concept and dose finding study. Within-participant comparisons and kinetic studies to establish dose-response curves will reduce the numbers of participants required in this early phase study. The study was designed after discussion with the clinical trials unit to be amenable to analysis by ANOVA. The trial design has benefited from independent peer-review by Ataxia UK a charity which has provided the funding and collaboration with Dr Vincenzo Libri.

The non-interventional study (Part IV) will investigate the use of novel, highly-sensitive technology to capture clinical deficit and measure subtle changes in ADL over a 9-12 month period and the use of a novel assays/techniques to improve the accuracy and specificity of FXN protein measurement, identify informative biomarkers that might track FXN levels and analyse the spatial organisation of the FXN locus. The Imperial Biomedical Research Centre provided funding for this part of the study.

The study benefits from ongoing advice from Professor Martin Wilkins and Dr Les Huson, statistician at the Imperial CRF.

1. LIST OF ABBREVIATIONS AND TERMS

3C-sequencing	Chromosome-Conformation Capture coupled with high-throughput sequencing
AE	Adverse Event
ADL	Activities of Daily Living
ADR	Adverse Drug Reaction
ALT	Alanine Aminotransferase
AP	Alkaline Phosphatase
AST	Aspartate Aminotransferase
BD	Twice a day
BM	Blood Glucose
BMI	Body Mass Index
BP	Blood Pressure
ChIP	Chromatin Immunoprecipitation
CI	Chief Investigator
CIF	Centre Imaging Facility
CK	Creatine Kinase
CRF	Case Report Form
CS	Clinically Significant
DeNDRoN	North Thames Dementias and Neurodegenerative Diseases Research Network
DNA	Deoxyribonucleic acid
DSMB	Data Safety Monitoring Board
DSUR	Developmental Safety Update Report
eCRF	electronic Case Report Form
EBV	Epstein–Barr virus
ECG	Electrocardiogram
EFACTS	European Fridreich’s Ataxia Consortium for Translational Studies
ELISA	Enzyme-linked immunosorbent assay
ENDIT	European Nicotinamide Diabetes Intervention Trial
EOS	End of Study
EU	European Union
EudraCT	European clinical trials database
FARS	Friedreich's Ataxia Rating Scale
fMRI	functional Magnetic Resonance Imaging
FXN	FXN
FRDA	Friedreich’s ataxia
g	Gram
GI	Gastrointestinal
GP	General Practitioner
Gamma-GT	γ-glutamyl transpeptidase
GCP	Good Clinical Practice (according to EC Note for Guidance)
GOT	Glutamate Oxalo-acetate Transaminase
GPT	Glutamate Pyruvate Transaminase
HIV	Human Immunodeficiency Virus
HPLC	High-performance liquid chromatography

HV	Healthy Volunteers
ICH	International Conference on Harmonisation
Imperial CRF	NIHR/ Wellcome Trust Imperial Clinical Research Facility
IMP	Investigational Medicinal Product
IRB	Institutional Review Board
IUD	Intrauterine device
IUS	Intrauterine system
IV	Intravenous
JRCO	Imperial College Joint Research Compliance Office
kg	Kilogram
LC-MS/MS	Liquid chromatography-tandem mass spectrometry
LDH	Lactate Dehydrogenase
LFT	Liver Function Test
LVLP	Last visit of last participant
mins	Minutes
mg	Milligram
MHRA	Medicines HealthCare Regulatory Agency
mM	milliMolar
MR	Magnetic Resonance
MRC DPFS	Medical Research Council Development Pathway Funding Scheme
MRI	Magnetic Resonance Imaging
mRNA	messenger RNA
MTD	Maximum Tolerated Dose
mtDNA	mitochondrial DNA
NCS	Not Clinically Significant
NHNN	National Hospital for Neurology and Neurosurgery
NIHR	National Institute for Health Research
OD	Once daily
PD	Pharmacodynamic
Q-RT-PCR	Quantitative real time polymerase chain reaction
PI	Principal Investigator
PIS	Participant Information Sheet
PD	Pharmacodynamic
PEV	Position Effect Variegation
PK	Pharmacokinetic
PRN	*Pro re nata*, as ‘required’
REC	Research Ethics Committee
RNA	Ribonucleic acid
RNA-seq	RNA-sequencing
RSI	Reference Safety Information
SAE	Serious Adverse Event
SADR	Serious Adverse Drug Reaction
SAR	Serious Adverse Reaction
SARA	Scale for the Assessment and Rating of Ataxia
SCAFI	SpinoCerebellar Ataxia Functional Index

SIT	Speech Intelligibility Test
SmPC	Summary of Product Characteristics
SRM	Selected Reaction Monitoring
SUSAR	Suspected Unexpected Serious Adverse Reaction
UK	United Kingdom
VAS	Visual Analogue Scale

2. BACKGROUND

2.1. What previous work is this project based on?

Friedreich's ataxia (FRDA) is an autosomal recessive neurodegenerative disease characterized by degeneration of large sensory neurons and spinocerebellar tracts, cardiomyopathy and increased incidence of blindness, deafness and diabetes. FRDA is the most frequent inherited ataxia and is caused by a partial deficiency in the mitochondrial protein frataxin (FXN) as a result of a GAA-repeat expansion in the first intron of the FXN gene (Campuzano et al., 1996). In spite of a broad variation in phenotype, this progressive disease usually presents in childhood and is characterized by impaired coordination, slurred speech, peripheral neuropathy and cardiomyopathy, frequently leading to severe disability by early adulthood. Interestingly, FXN messenger ribonucleic acid (mRNA) is predominantly expressed in tissues with a high metabolic rate, including liver, kidney, brown fat and the heart. Although the exact function of FXN protein is still unknown, yeast strains carrying a disruption in the FXN homologue gene (YFH1) showed a severe defect in mitochondrial respiration and loss of mitochondrial Deoxyribonucleic acid (mtDNA) associated with elevated intra-mitochondrial iron (Koutnikova et al., 1997). Most of these mitochondrial defects have also been described in FRDA cell lines and conditional FXN knockout mouse models (Puccio and Koenig, 2002) which have less than 50% of normal levels of FXN protein. In participants, FXN repression leads to pathogenic levels of FXN with a threshold of below 25-30% of normal. Carriers have approximately 50% of normal FXN levels and are asymptomatic. Therefore a radical treatment would be to upregulate FXN expression to approximately carrier level (Festenstein, 2006; Gottesfeld, 2007). Previous work in our laboratory has established that the pathological GAA-repeat expansion that occurs in FRDA can induce gene silencing that resembles the classical epigenetic phenomenon of position effect variegation (PEV) (Saveliev et al., 2003). Such gene silencing leads to variegation of expression of the affected gene in a proportion of cells. Importantly, the extent of silencing can be modified by altering the dosage of enzymes that modify chromatin and/or the proteins that bind to these modifications (Festenstein et al., 1999). The basic subunit of chromatin, the nucleosome, consists of histone proteins whose tails protrude from the packaged structure and can be post-translationally modified to mark the gene as active or inactive (Jenuwein and Allis, 2001). We have recently shown using mouse models of PEV that the tail of histone H3 is hypoacetylated in cells in which the gene is silenced and hyper-acetylated when expressed. Moreover, the classical silencing modification, histone H3 (H3) lysine 9 (K9) trimethylation (me3) is associated with the transcriptionally silenced state whereas histone acetylation was associated with the active state (Hiragami-Hamada et al., 2009). Moreover H3K9me3 has been found associated with silencing at the FXN locus in participants and a mouse model for FRDA (Al-Mahdawi et al., 2008). This led us and others to investigate the possibility that inhibition of histone deacetylases may increase acetylation thereby preventing H3K9 methylation (as acetylation and methylation are mutually exclusive) leading to suppression of variegation and reduced silencing (Festenstein, 2006). It has previously been shown that a specific histone deacetylase (HDAC) inhibitor, 4b and/or its derivatives can upregulate the FXN gene in cells from participants and in a mouse model (Herman et al., 2006; Rai et al., 2008). We have tested the effect of a number of HDAC inhibitors on Epstein–Barr virus (EBV) transformed cells derived from participants, primary cells and a mouse model (Al-Mahdawi et al., 2008) for FRDA (N. Rothe PhD thesis). Strikingly, our results revealed that Vitamin B3 (nicotinamide, a classical class III HDAC inhibitor) treatment of primary lymphocytes from FRDA participants after incubation for 24-72 hours restored their FXN levels to normal (~3 fold upregulation – Figure 1 upper panel) the latter result was confirmed by the Pandolfo lab (Brussels -personal communication). We went on to show in an *in vivo* study that nicotinamide treatment upregulates FXN in cerebellum (a tissue affected in FRDA) derived from a mouse model for FRDA by ~1.5 fold (at the protein level) when injected intraperitoneally after 24hours and this effect is sustained after daily dosing for 5 days (Figure 1 lower panel). As high-dose nicotinamide has been administered safely to humans this opens up the possibility of a readily available therapy for FRDA. The aim of the present study is to investigate whether nicotinamide can upregulate the FXN gene in humans *in vivo* as this would potentially provide a radical treatment for this incurable and frequently devastating disease.

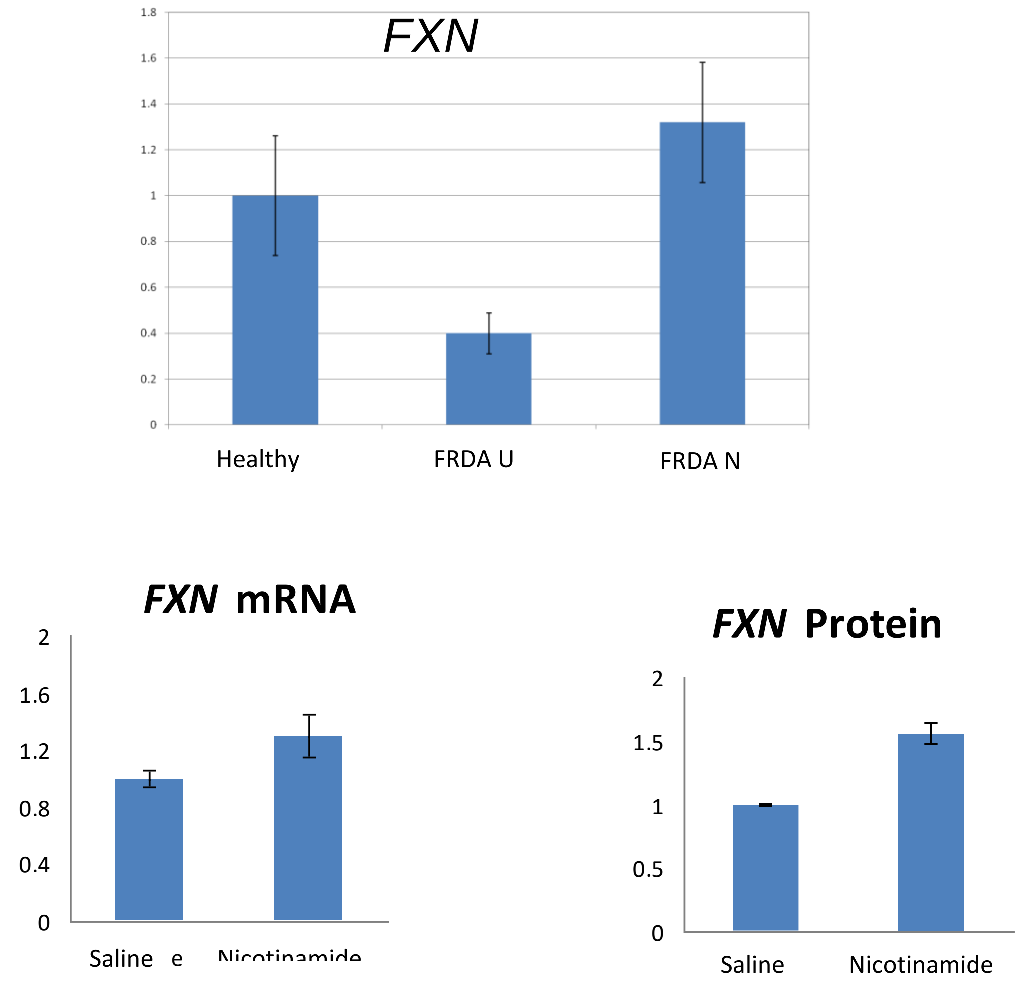

Figure 1:

Upper Panel: Nicotinamide treatment (10mM for 72 hrs) of primary lymphocytes from participants (FRDA N) upregulated Frataxin mRNA to normal levels (Healthy U). FRDA U= untreated participants; y axis: data are relative Frataxin level normalized to healthy (n=4 healthy and n=5 FRDA); Error bars= SD; P<0.05 students 't' test; FRDA N vs. FRDA U).

Lower Panel: Nicotinamide upregulated cerebellar expression of Frataxin in a mouse model of Friedreich's ataxia. Mice were given daily doses of either nicotinamide (~500mg/kg) or saline for 5 days. This dose was tolerated well by the mice in line with previous pharmacodynamic studies comparing humans with mice (Horsman, 1993). FXN Protein estimation from cerebellar extract was quantified using the mitosciences dipstick assay. (n=5; error bars= SD) P<0.05 students 't' test; Saline vs Nicotinamide.

2.2. Clinical Experience with Nicotinamide

Nicotinamide has a good safety record and has been given to those at risk of diabetes at high-dose (3g/day) for prolonged periods (5 years) and was tolerated well with no serious adverse effects (SAEs) in the European Nicotinamide Diabetes Intervention Trial (ENDIT). It has also been used at high-dose (6-10g/day) to pre-treat participants undergoing radiotherapy (Horsman et al., 1993).

2.3. Why is a study needed now?

There is currently no specific treatment for FRDA which is a disease with significant morbidity that shortens life considerably in many affected individuals. The recent discovery that HDAC inhibitors can upregulate the FXN gene in cells from participants has revealed the potential for a radical new therapy for this disease (Festenstein, 2006). Nicotinamide treatment of cells from FRDA participants is effective in upregulating FXN. The logical next step is to determine whether the upregulation in participant cells will occur *in vivo* in humans hence the necessity for this study.

It is well known that clinical trials for efficacy in FRDA are hampered by the difficulty in measuring the progression of ataxia based on existing clinical scales. Part IV will permit us to develop reliable methodology to measure clinical deficit and more subtle change in the neurological state of FRDA participants over a 9-12 month period and will allow us to correlate functional changes to levels of expression of FXN protein and the epigenetic structure of the FXN gene. This part of the study will be conducted without the use of nicotinamide.

2.4. Outcomes and impact

A proof-of-concept study is essential for a go/no-go decision on the use of nicotinamide in FRDA. This study will seek evidence for the ability of nicotinamide to upregulate FXN in participants with FRDA. If the data support a potentially useful therapeutic effect provide biological plausibility through its mechanism of action and suggest the drug is safe, the study will be used to power a study to address clinical efficacy. Nicotinamide is orally active and inexpensive. If effective, it would provide a much needed cost-effective radical therapy for FRDA.

2.5. How will the results of the study be taken forward?

These data will be used to design a large scale follow-on study in FRDA participants to define efficacy based on acceptable endpoints for this condition. Typically this would involve analysis of disability by the Scale for the Assessment and Rating of Ataxia (SARA, Burk et al., 2009), quality of life and time-to-clinical worsening. Such a study would be double-blind and require significant numbers of participants (perhaps 200). The study proposed in this project would enable realistic power calculations and the development of reliable clinical tools for a follow-on study. The recent establishment of an FP7 European Network (European Friedreich's Ataxia Consortium for Translational Studies, EFACTS) would provide the necessary framework for such a large scale trial.

3. STUDY OBJECTIVES

3.1. Primary Objective

The primary outcome is to assess whether, and at what dose, oral nicotinamide can upregulate the pathologically silenced FXN gene in FRDA in peripheral resting lymphocytes.

3.2. Secondary objectives

Secondary outcomes:

- Assess the mechanism of upregulation of FXN using chromatin immunoprecipitation studies.
- Determine the safety and tolerability of nicotinamide in FRDA participants.
- Assess the long-term clinical effects in FRDA participants of taking nicotinamide daily over a two month period.
- Assess additional FRDA biomarkers using gene expression profiling.

In Part IV:

- Investigate the use of novel, highly-sensitive technology such as functional Magnetic Resonance Imaging (fMRI), and motion-tracking equipment to better capture clinical deficit and measure subtle changes in activities of daily living (ADL).
- Improve the accuracy and specificity of FXN protein measurement using a novel targeted mass spectrometric assay (Liquid chromatography-tandem mass spectrometry, LC-MS/MS) compared with the dipstick assay (Mitosciences).
- Identify informative biomarkers that might track FXN levels using Ribonucleic acid sequencing (RNA-seq).
- Analyse the spatial organisation of the FXN locus by Chromosome-Conformation Capture coupled with high-throughput sequencing (3C-sequencing) to correlate with FXN expression to potentially identify participants that would be predicted to respond to epigenetic therapies such as nicotinamide.

4. STUDY DESIGN

4.1. Overall Study Design and Plan Description

A minimum of ten and up to twenty FRDA participants (for each part of the study) over the age of 18 years with a molecular genetic diagnosis of FRDA, who have GAA-repeat expansions on both alleles, attending the neurogenetics clinics at Imperial College Health Care Trust or the National Hospital for Neurology and Neurology (where the PI is a Consultant Physician in Neurogenetics) will be identified and clinically assessed as out-patients using the SARA scale for level of disability (Burk et al., 2009) and screened for FXN level as well as full blood count, urea and electrolytes, blood glucose (BM), liver function tests (LFTs) and electrocardiogram (ECG). Any serious intercurrent abnormalities or illness will exclude the participants from this study. These investigations will all be repeated at the conclusion of the treatment period. A participant information sheet (PIS) explaining the nature of the study will be given to the participant (via email or post) and to the General Practioner (GP; via fax or post). We will not actively inform GPs of participants participation in Part IV (i.e if participants are only participating in this part only). Informed consent will be obtained from the participant prior to taking part in the study. As nicotinamide has previously been given to humans with few adverse effects (AEs) we do not anticipate serious adverse effects (SAEs). It will be explained to the participants and GPs the possible side effects they may experience based on previous studies (Horsman et al., 1993). Moreover, several pharmacokinetic (PK) studies on healthy volunteers (HV) and participants compared with mice (Horsman et al., 1993) showed that an effective oral dose of 6g of nicotinamide daily (approximately 80-100mg/kg) which will achieve a plasma concentration of approximately 1mM results in minimal side effects (Stratford et al., 1992). Our interventional study will have three parts. Part I will be a single dose escalation study. Part II will be the first part of the multiple dose study and will investigate the clinical effects of daily dosing

for a period of five days. If this multiple dose dosing regimen is well tolerated and upregulation of FXN level are observed, participants will take nicotinamide daily and come in for weekly visits over a two month period (Part III). Both single and twice daily dosing may be explored. Those who meet the inclusion criteria will be admitted to the Imperial CRF at the Hammersmith Hospital under the care of resident staff and Professor Festenstein. Part IV will develop reliable methodology to measure clinical deficit and more subtle changes in the neurological state of FRDA participants over a 9-12 month period without nicotinamide. We will also correlate functional changes to levels of expression of FXN protein and the epigenetic structure of the FXN gene. Participants will come in for eight visits (or 4 overnight stays) over the course of 9-12 months. Up to 20 HV will also take part in Part IV and will only come for two visits.

1) Single dose escalation study, Part I

Firstly, we will treat the participants with a single dose of 2g of nicotinamide given orally in tablet form. We will measure the FXN levels before treatment and at 2 (± 15 mins), 4 (± 25mins), 8 (± 1hr) and 24 (± 2.5hrs) hours after treatment on separated peripheral blood lymphocytes. Plasma will be stored so that nicotinamide concentrations can be assessed by high-performance liquid chromatography (HPLC) as previously described (Stratford et al., 1992). In addition, chromatin will be extracted and stored at -80°C. In the absence of FXN upregulation we will escalate the dose following a 'flushing out' period of at least one week. We will escalate by 2g increments to 6g and then 1g increments to a maximum of 8g until FXN upregulation is achieved and in the absence of AEs that, in the opinion of the Chief Investigator (CI) or his delegated doctors, may put the safety of the participant at risk. At 8g of nicotinamide we expect that some participants might experience nausea. Participants will be assessed throughout for nausea using a visual analogue scale (VAS). Those with significant nausea will be offered anti-nausea treatment (typically metoclopramide or domperidone) to avoid vomiting. In the event of vomiting occurring, intravenous (IV) fluid will be given as required.

A minimum of ten participants will be recruited in this part of the study according to the schema shown in the table below. Two participants per visit will receive no nicotinamide. This will allow within-participant comparisons and control for the study procedures. The dose escalation will cease when we have reached the maximum tolerated dose (MTD), at least 5-fold upregulation in 50% of participants, or 8g nicotinamide, whatever comes first.

Table 1: Recruitment schema for Part I

	Visit 1	Visit 2	Visit 3	Visit 4	Visit 5
Participant 1	D1	D2	D3	D4	No
Participant 2	D1	D2	D3	D4	No
Participant 3	D1	D2	D3	No	D5
Participant 4	D1	D2	D3	No	D5
Participant 5	D1	D2	No	D4	D5
Participant 6	D1	D2	No	D4	D5
Participant 7	D1	No	D3	D4	D5
Participant 8	D1	No	D3	D4	D5
Participant 9	No	D2	D3	D4	D5
Participant 10	No	D2	D3	D4	D5

No = No treatment
D1 = 2g of Nicotinamide
D2 = 4g of Nicotinamide
D3 = 6g of Nicotinamide
D4 = 7g Nicotinamide
D5 = 8g of Nicotinamide , Maximum Tolerated Dose (MTD) or dose found in visits 1-4 capable of upregulating FXN by at least 5-fold.

2) Multiple dose study, (Parts II and III)
There will be two parts to the multiple dose study. Both parts will be pursued following the completion of the single-dose study. There will be at least a one week washout for participants having participated in Part I and going into Part II.

Evaluation of the effect of daily dosing for a period of five days, Part II
Part II of the study will enable an evaluation of the effect of daily dosing for a period of five days to determine whether FXN is upregulated in a sustained manner.

Participants will be admitted to the Imperial CRF for four nights. It may be possible for some participants to return home every evening. This would need to be agreed by the CI.

To enable within participant comparisons, where possible, this study will be performed on the same participants already recruited for the single dose escalation part. If required, additional participants will undergo screening and if eligible may enter directly into this part of the study. The ratio of participants that will be 'on no treatment' to those on 'on treatment' will be 1:4 (i.e one patient will be on 'no treatment' to 4 'on treatment'). Participant's allocation to no drug will be determined at the CI's discretion and discussed and agreed with the participant.

Participants will be treated with 3.5 grams of nicotinamide on the first day. On subsequent days the dose will be escalated to MTD but not higher than 4 grams on day two, 5 grams on day three and 6 grams on days four and five. The daily dosing regimen may be modified at the CI's discretion on clinical grounds. The daily dosing regimen may also be modified from once to twice a day for some participants at the CI's discretion on clinical grounds. Dose escalation will occur only in the absence of AEs that, in the opinion of the CI or his delegates, may put the safety of the participant at risk. If the MTD is reached in one or more participants at any time during the 5-day treatment period, the dose escalation schedule may be adjusted in all the remaining participants to prevent the occurrence of AEs (e.g. excessive nausea, vomit etc) that may put the safety of the participant at risk. In order to avoid nausea, participants will be administered anti-nausea treatment if required (domperidone, 20 mg oral or metoclopromide) prophylactically prior to dosing and post-dosing (domperidone, 20 mg oral up to four times daily; metoclopramide 10-20 mg IV/orally *Pro re nata* [(PRN) up to three times daily]. Participants and their carers will complete Part II of Friedreich's Ataxia Rating Scale (FARS) which is a subscale that measures ADL (Appendix 3) in participants with Friedreich's ataxia. In addition neurological assessments such as SARA and SpinoCerebellar Ataxia Functional Index (SCAFI) scales (Appendices 1 and 2) and a clinician- and/or software-based speech dysarthria assessment will also be performed on day 1 (to count as baseline measurement) and day 5.

Long term assessment of FRDA participants taking nicotinamide daily over a two month period, Part III
If the multiple dose dosing regimen of nicotinamide in Part II is well tolerated and changes in FXN level are observed, all participants (including those who were scheduled to receive no drug in part II) will be offered to enter Part III. Participants who took part in part I but not in part II of the study may also participate. These participants may require re-screening. This decision will be taken by the CI. If required, additional participants will undergo screening and if eligible may enter directly into this part of the study. Participants who took part in Part II will be given the weekly drug supply and diary on Part II Day 5. Participants who took part in Part I but not Part II or participants who will directly enter Part III will be given the weekly drug supply and diary at their screening visit (only once determined eligible) or by courier / recorded delivery post. Their next visit will be Part III, week 1.

Participants who were 'on drug 'in Part II will take their MTD already established over the course of two months. If any AEs occur that in the CI's opinion may put the safety of the participant at risk, the dose schedule will be adjusted. If a participant was on 'no drug' in Part II or due to enter Part III directly they will start at a low dose of nicotinamide (typically 2g) which will be gradually escalated in the absence of any AEs which in the CI's opinion may put the participant's safety at risk.

If the participant stops taking nicotinamide for several days (for safety reasons), only once the participants AEs are fully resolved, the CI may decide to add this drug interruption period onto the end of the two month period.

To ensure that sustained FXN upregulation is achieved, the nicotinamide PK/ pharmacodynamic (PD) relationship (i.e. time to nicotinamide steady state vs. upregulation of FXN) will be evaluated at each weekly visit and the daily dose regimen may be modified from once to twice a day [i.e. from 6 grams (or MTD) Once Daily (OD) to 3 grams (or ½ MTD) twice a day (BD)]. In some instances, manufacturing restrictions (i.e. nicotinamide tablets of 500mg) render the division of the dose into two equal doses, for BD dosing, impossible. In these cases patients will receive a slightly different dose in the morning to the evening.

Participants will attend the Imperial CRF prior to taking their dose of nicotinamide once a week (7 ± 2 days) over this time period. Participants will be provided with study drug supply for 7 ± 2 days and rescue medication (anti-nausea treatment, typically domperidone or metoclopromide). Domperidone or metoclopromide will be taken if required prophylactically [domperidone pre-dose (20 mg oral) and post-dose (20 mg orally up to four times daily)]. Participants will also be provided with their first weekly diary card, which will need to be completed each day for the entire duration of Part III until the interventional end-of-study visit. Participants will record whether or not they took nicotinamide, number of tablets, any AEs and any concomitant medications taken. Each week, the trial diary from the previous visit will be collected and participants will be provided with another diary to complete starting on the next day. Participants and their carers will complete Part II of the Friedreich's Ataxia Rating Scale (FARS): ADL. SARA and SCAFI scales will also be done and a clinician- and/or software-based speech dysarthria assessment will be performed.

3) Development of reliable methodologies to measure clinical deficit and subtle neurological changes and correlation of functional changes to levels of expression of FXN protein and the epigenetic structure of the FXN gene (Part IV).

A minimum of ten up to a maximum of 20 FRDA participants will be offered the opportunity to participate in Part IV. Up to 20 healthy volunteers (HV) will also participate in this part of the study.

Healthy volunteers will have an fMRI assessment during the execution of tasks, perform the SARA and SCAFI and scenarios wearing a body suit, perform eye movement tasks (optional) and perform a balance control task (optional). These assessments do not have to be performed on the same day. Including HV will provide us with a control comparison in individuals without a progressive deterioration.

Participants may be contacted by telephone and/or email or at their study visits (i.e FRDA participants participating in the interventional study) by the CI or delegates to inform them about the potential benefit and any risks associated with participation in this part of the study. fMRI contraindications will be detailed. Participants will be given the PIS and given as much time as they wish to read and ask questions. If the participant wishes to take part, informed consent will be given in writing prior to performing any study-related assessments. Participants will be assessed for fMRI contraindications during screening (including urine pregnancy test for women of child-bearing potential) prior to study start.

FRDA participants will undergo the assessments detailed below over the course of 2 days at the study start, 2 weeks (± 2 days), 3 months (± 2 weeks) and at 9-12 months (± 3 months). In some cases if travel is difficult, participants will be offered overnight accommodation (at the Imperial CRF or private hospital accommodation).

1. Urine pregnancy test only for women of child-bearing potential (prior to fMRI).
2. Blood samples will be taken for:
    - FXN protein estimation by LC-MS/MS compared to the current dipstick assay (Mitosciences);
    - RNA-seq to identify informative biomarkers that might track FXN levels and
    - Analysis of the spatial organisation of the FXN locus by Chromosome-Conformation Capture coupled with high-throughput sequencing (3C-sequencing) to correlate with FXN expression – such a technique may be capable of identifying participants that would be predicted to respond to epigenetic therapies such as nicotinamide.
3. The Edinburgh handedness score will be performed at the study start visit only.
4. Participants will perform standardised tasks derived from the SARA and SCAFI
5. fMRI assessment during the execution of a standardised finger-tapping task (Akhlaghi et al., 2012) and object manipulation. fMRIs will be performed at the Imperial College Centre Imaging Facility (CIF). Participant's hands will be filmed on an fMRI compatible device, which analyses movements in real-time and will be correlated with the fMRI analysis.
6. Participants will be fitted with a simple portable motion-capture device similar to an ECG to be worn overnight. This will be returned the following day.
7. Participants will perform standardised ADL tasks (Appendix 5) and the SARA and SCAFI (not the timed speech test) while wearing a full-body, motion-tracking equipment ('body suit') to capture detailed body kinematics.
8. Chromatin immunoprecipitation studies. Consistent with a recent study (De Biase et al., 2009) we have shown that both H3K27me3 and H3K9me3 spreads on either side of the GAA-repeat when the gene is repressed in participant cells. Samples taken at each visit in part IV will measure the stability of either of these modifications over time and correlate this with clinical phenotype.
9. Participants will perform standardised eye movement tasks to assess the performance of their occulo-motor system quantitatively assessed using a binocular eye-tracking system (optional).
10. Participants will stand on a force plate and their balance performance will be measured (optional).

To enable identification of pathological changes specific to FRDA, identical studies will be performed on healthy volunteers. Healthy volunteers will come over the course of several visits and will perform the following assessments:

1. Urine pregnancy test for women of child-bearing potential only (prior to fMRI).
2. The Edinburgh handedness score.
3. fMRI assessment during the execution of the standardised finger-tapping task (Akhlaghi et al., 2012) and object manipulation. Participant's hands will be filmed on an fMRI compatible device, which analyses movements in real-time and will be correlated with the fMRI analysis.
4. Participants will perform standardised ADL tasks (Appendix 5) and the SARA and SCAFI (not the timed speech test) while wearing a full-body, motion-tracking equipment ('body suit') to capture detailed body kinematics.
5. Participants will perform standardised eye movement tasks to assess the performance of their occulo-motor system quantitatively assessed using a binocular eye-tracking system (optional).
6. Participants will stand on a force plate and their balance performance will be measured (optional).

These assessments can be performed over the course of several days or at different time points.

4.2. Discussion of Study Design

This is an early phase study to ascertain whether upregulation of FXN in participants with FRDA can be achieved with nicotinamide given orally and if so to determine the effective dose. We will determine the safety and tolerability of oral nicotinamide in participants with FRDA when used in a dose range similar to that used in a long-term trial in the prevention of diabetes and in the treatment of participants with radiotherapy. We will also assess the long term clinical effects of FRDA participants taking nicotinamide

daily over a two month period. In addition, the protocol allows for assessment of the downstream effects of FXN on biological pathways using RNA-Seq and the effect on the epigenetic state of the FXN gene following HDAC treatment and a correlation with phenotype to be made.

Part IV will permit us to develop reliable methodology to measure clinical deficit and more subtle change in the neurological state of FRDA participants over a 9-12 month period and will allow us to correlate functional changes to levels of expression of FXN protein and the epigenetic structure of the FXN gene. This part of the study will be conducted without the use of nicotinamide.

Including HV in Part IV will be used to establish a ‘normal’ baseline. This baseline will provide a way to compare FRDA patients to HVs and enable new clinical scales based on the novel methodology to be established.

4.3. Selection of Study Population

Participants eligible for the study will be drawn from the FRDA patient population being seen at Hammersmith Hospital, or the National Hospital for Neurology and Neurosurgery (NHNN) or who have contacted us via information posted on ClinicalTrials.gov.

Healthy volunteers will be recruited from the research community or the Imperial CRF health volunteer database. Advertisements will be posted and placed online on the ICRF website.

4.4. Inclusion Criteria for the interventional study

1. Participants must have a molecular genetic diagnosis of FRDA, consisting of a GAA-repeat expansion on both alleles of the FXN gene.
2. Participants must be over the age of 18 years living in the UK and registered with a GP.
3. Participants must provide informed consent. If written consent is not possible due to physical incapacity, written consent on behalf of the participant will be sought from the participant’s relatives or carer.
4. A female participant is eligible to participate if she is of:

Non-childbearing potential defined as pre-menopausal females with a documented tubal ligation or hysterectomy; or postmenopausal defined as 12 months of spontaneous amenorrhea [in questionable cases a blood sample with simultaneous follicle stimulating hormone (FSH) > 40 MlU/ml and estradiol <40 pg/ml (<140 pmol/L) is confirmatory].

Child-bearing potential and agrees to use one of the following contraception methods:
True abstinence: When this is in line with the preferred and usual lifestyle of the participant. [Periodic abstinence (e.g.,calendar, ovulation, symptothermal, post-ovulation methods) and withdrawal are not acceptable methods of contraception].

Contraceptive Methods with a Failure Rate of < 1%:

- Oral contraceptive, either combined or progestogen alone;
- Injectable progestogen;
- Implants of levonorgestrel;
- Estrogenic vaginal ring;
- Percutaneous contraceptive patches; -
- Intrauterine device (IUD) or intrauterine system (IUS) that meets the <1% failure rate as stated in the product label;
- Male partner(s) sterilisation (vasectomy with documentation of azoospermia) prior to the female participant's entry into the study;

- Double barrier method: condom and occlusive cap (diaphragm or cervical/vault caps) plus vaginal spermicidal agent (foam/gel/film/cream/suppository).

4.5. Exclusion Criteria for the interventional study

1. Participants with significant clinical dysphagia.
2. Participants taking Sodium Valproate or any other known histone deacetylase inhibitor.
3. Participants taking part in another interventional clinical trial or who have done so within 30 days before screening.
4. Participants known to be positive for human immunodeficiency virus (HIV).
5. Participants with any additional medical condition or illness that, in the opinion of the CI would interfere with study compliance and/or impair the participant's ability to participate or complete the study. Concurrent diseases or conditions that may interfere with study participation or safety include liver disease, bleeding disorders, arrhythmias, organ transplant, organ failure, current neoplasm, poorly controlled diabetes mellitus, poorly controlled hypertension, clinically significant haematological or biochemical abnormality.
6. Patients with a history of substance abuse (e.g. alcohol or drug abuse) within the previous 6 months before enrolment.
7. Participants with a history of severe allergies.
8. Female participants who are lactating or pregnant (positive pre-randomisation serum pregnancy test) or plan to become pregnant during the study.
9. Hypersensitivity to Nicobion (nicotinamide) or any of the excipients in this preparation
10. Liver function tests outside the normal range: aspartate aminotransferase (AST), alanine aminotransferase (ALT), bilirubin which in the opinion of the CI would put the participant's safety at risk.

4.6. Inclusion Criteria for the non-interventional study (Part IV)

1. Up to 20 participants must have a molecular genetic diagnosis of FRDA, consisting of a GAA-repeat expansion on both alleles of the FXN gene.
2. Up to 20 HV participants
3. Participants are over the age of 18 years, living in the UK and registered with a GP.
4. Participants must provide informed consent. If written consent is not possible due to physical incapacity, written consent on behalf of the participant will be sought from the participant’s relatives or carer.
5. Women of child-bearing potential must have a negative urine pregnancy test.

4.7. Exclusion Criteria for the non-interventional study (Part IV)

1. Contraindications to MRI including, but not limited to: intracranial aneurism clips (except Sugita), history of metal lathe work or possibility of intra-orbital metal fragments, pacemakers and non-MR compatible heart valves or other non-MR compatible implants, history of claustrophobia or participant feels unable to lie still on their back for a period of 60-90mins in the fMRI scanner.

4.8. Participant Completion

Participants will be considered complete for the purpose of the interventional study once they have completed all procedures at the end of interventional study visit. The end of interventional study is defined as the last visit of the last participant (LVLP) undergoing the interventional trial.

FRDA participants who will take part in part IV of the study will have their last visit at 9-12 months.

We do not recommend that participants continue to take nicotinamide post-study as further clinical trials are required to determine whether or not nicotinamide is safe and is of clinical benefit to FRDA patients. If

participants still decide to take nicotinamide against our advice, they will be referred to their GP with recommendation of regular safety follow up to evaluate any nicotinamide-induced toxicity. We may request the FRDA participants consent to come in for visits at the Imperial CRF at the CI's discretion. Blood tests (FXN/chromatin, safety bloods etc) and neurological and medical examinations (SARA, SCAFI, ADL, ECG, vital signs etc) may be performed at these visits.

All FRDA participants will be offered the possibility to enter a large scale follow-on study which is being planned to define efficacy based on acceptable endpoints for this condition.

4.9. Withdrawal of Participants from Study

At any time, participants may withdraw from the study (i.e. withdraw consent to participate) at their own request and without giving reasons. Participation in the study may be discontinued at any time at the discretion of the CI and in accordance with their clinical judgment. No disadvantage will arise for any participant who withdraws consent for participation at any time or who is withdrawn from the study by the CI.

Reasons for discontinuation of study treatment will be recorded on the electronic Case Report Form (eCRF) in any case and may include the following:

- Participant's request for withdrawal
- CI's decision that discontinuation is in the best interest of the participant
- Non-compliance with the regimen and timing
- Development of an intolerable AE due to study participation as determined by the CI, participant or both
- Development of an intercurrent illness, condition, or procedural complication, which would interfere with the participant's continued participation
- Discovery that the participant entered the study in violation of the protocol or occurrence of a significant protocol violation during the study
- Data that becomes available that raises concern about the safety of the study drug so that continuation would pose potential risks to the participant
- Participant becomes pregnant
- Participant is lost to follow-up
- Any participant who discontinues treatment for medical reasons, e.g. because of AEs or clinical laboratory abnormalities, should be followed up at medically appropriate intervals in order to evaluate the course of the event and to ensure reversibility or stabilisation of the abnormality or event. The subsequent outcomes of these events will be recorded on the eCRF. However, if these events are attributable to deterioration of the disease, no such measures will be suggested.
- If a participant fails to return for a scheduled visit, a documented effort must be made to determine the reason. All attempted contact (telephone, emails or letters) must be filed in the participant's medical notes.

An Interventional End of Study (EOS) Visit will be done for each participant who completes the interventional study or who receives any study drug and withdraws prematurely from the study.

It may be possible in certain exceptional circumstances for a participant to withdraw from one part and enter another part of the study (for example: withdraw from Part II however enter Part III) however this arrangement would be agreed by the CI and participant.

In case of premature termination or suspension of the study, the CI will promptly inform the Research Ethics Committee (REC) and Medicines HealthCare Regulatory Agency (MHRA) of the termination or suspension and the reason for that action.

4.10. Prior and Concomitant Therapy

Participants may enter this study receiving any concomitant therapy except for those specifically prohibited in the exclusion criteria. The dose of concomitant treatments should remain stable for 8 weeks prior to entry in the study. The CI or delegates may prescribe additional medications (see 6.7) during the study, provided the prescribed medication is not prohibited by the protocol. Any additional medication or adjustment to medication must be recorded in the eCRF.

4.11. Dose Reduction Guidelines for Potential Side Effects

Based on experience to date with nicotinamide the most likely side effect that might interfere with participant compliance is nausea/vomiting. Use of a VAS (shown below) to monitor this subjective effect combined with the dose-escalation study will establish if particular participants are susceptible and then the dose escalation adjusted to prevent excessive nausea.

Visual Analogue Scale:

How do you rate your nausea on a scale of 1 to 10, 1 being the least severe to 10 being the most severe?

1	2	3	4	5	6	7	8	9	10

In addition if clinically appropriate, anti-nausea agents (for example: domperidone or metoclopramide etc.) will be given as required. In order to avoid nausea in Part II, participants will be administered anti-nausea treatment prophylactically prior to dosing if required. In Part III, participants will be provided with rescue medication to take prophylactically prior to dosing (for example: domperidone 20 mg oral or metoclopromide) and post-dose if required (for example: domperidone 20 mg oral up to four times daily or metoclopromide). Participants will be asked to keep an accurate record of the use of rescue medication in the weekly trial diary.

Although nausea is the most likely side effect in the recently published ENDIT study which administered high-dose nicotinamide (3g/day) on a long term basis this side effect was rarely reported (<0.5%). It was also rarely reported in a study of higher doses of nicotinamide (6-9g) given to normal volunteers taking nicotinamide in tablet form (Horsman et al., 1993).

5. TREATMENTS

5.1. Study Product, Dose, and Route of Administration

The study drug product is Nicotinamide. Nicotinamide is a commercially available as Vitamin B3. 0.5g tablets are readily available under the trade name Nicobion from Teofarma.

5.2. Metabolism

Nicotinamide is readily metabolised in the liver and its metabolites are rapidly excreted via the kidney.

5.3. Pharmacokinetics

Nicotinamide has high bioavailability and is readily absorbed via the Gastrointestinal (GI) tract, it passes the blood brain barrier and when given orally reaches peak plasma concentrations within 30 mins to 1 hour. It is rapidly cleared from the circulation and has a wide tissue distribution. Pharmacodynamic studies in humans reveal a half-life of between 5 and 7 hours.

5.4. Rationale for Nicotinamide Dose Selection

The dose range chosen in this study is based upon those previously used in humans in long-term studies (ENDIT, Gale et al., 2004) and short-term studies (Horsman et al., 1993) as well as the levels required to achieve upregulation of FXN in cells obtained from FRDA participants and in a mouse model of FRDA (see above). Mouse studies suggest that a single dose can lead to upregulation of FXN 24hrs post-dose therefore daily dosing is likely to be sufficient. It is difficult to extrapolate directly from the mouse to the human due to species differences. However, the mouse studies are useful in that they showed upregulation of FXN in the affected tissues. At present it is not known what dose would be effective in humans which is the purpose of this study.

The dose range chosen here was based on 2 criteria: 1) doses shown to be well tolerated in previous studies in humans (Horsman et al., 1993; Gale et al., 2004) and 2) our studies on primary lymphocytes which showed that exposure to nicotinamide between 1-10mM will upregulate FXN by up to 3 fold *in vitro* (see background). Oral dosing of nicotinamide at 6g has previously been shown to achieve plasma nicotinamide concentrations within this range in human volunteers (Stratford et al., 1996). FXN levels in asymptomatic carriers are 40-50% of normal (Pianese et al., 2004) and FRDA patients develop the disease at levels less than 30%. Therefore, a 1.5 to 5 fold upregulation would potentially restore FXN levels to those found in asymptomatic individuals. However, even more modest upregulation may be of therapeutic benefit. Therefore, for this study which will also look at tolerability we decided on a dose range between 2g and 8g daily. If effective in upregulating FXN towards carrier levels and/or well tolerated further studies will be designed to assess nicotinamide efficacy in FRDA patients.

5.5. Study Product

A highly purified form of nicotinamide which has been shown to be well tolerated orally and is readily available for therapeutic use will be used for this study. The preparation of nicotinamide has been sourced by the Hammersmith Hospital Pharmacy who will dispense the drug. The IMP may be dispensed up to 2 days prior to the participant's visit. In Part I, the IMP will be dispensed at each visit. In part II, the IMP will be dispensed for the whole week. In Part III, the IMP will be dispensed at each weekly visit.

Participants will be given the drug supply required either on PIID5 (for participants participating in Part II) or at the screening visit / recorded delivery post (for participants who participated in part I but not in part II / participants who will directly enter into Part III) until their next visit (Part III, week 1).

5.6. Storage

The study product will be packaged and dispensed either into sterile, light-proofed bottles or in blister packaging.

5.7. IMP Labelling

The contents of the label will be in accordance with all applicable regulatory requirements. The Hammersmith Hospital pharmacy will be responsible for drug preparation.

6. EFFICACY AND SAFETY VARIABLES

6.1. Safety Variables

The criteria for evaluation of safety will be the following: physical examination, routine clinical laboratory tests (LFTs, hematology, chemistry, BM - assessed using a stick and urinalysis; before and after treatment), ECG, clinical examination including blood pressure (BP), pulse and respiratory rate etc.

6.2. Efficacy variables

The primary efficacy measure will be upregulation of FXN levels as measured by Q-RT-PCR and the Mitosciences dipstick assay, western blotting and mass spectrometry may employed for further verification of the FXN protein levels and RNA-seq for the FXN mRNA and biomarker levels.

In part IV, we will evaluate FXN protein estimation by LC-MS/MS as opposed to dipstick assay and analysis of the spatial organisation of the FXN locus by 3C-sequencing in FRDA participants only.

7. OBSERVATION AND METHODS

7.1. Physical Examination and Medical History

Physical examinations will be performed to ensure suitability according to the inclusion and exclusion criteria at screening and baseline and to document health status. The physical examination comprises measurement of height and body weight (at screening only) and a routine medical examination including an abbreviated measure of neurological function by use of the SARA scale to assess degree of disability (Burk et al., 2009).

A medical history will be recorded at screening only. The medical history will elicit information concerning existing medical conditions, major illnesses, and related surgical procedures. Any prescribed or over-the-counter medications that the participant received within the past 30 days will be recorded on the eCRF. Medication prescribed for the treatment of FRDA for the 8 weeks prior to enrollment will also be recorded on the eCRF. Participants will be instructed to notify the study doctor before beginning newly prescribed or over-the-counter medications.

Participants and their carers will complete Part II of FARS which is a subscale that measures ADL in participants with FRDA on day 1 (to act as baseline) and day 5 of Part II and bi-monthly in Part III. In addition neurological assessment (SARA and SCAFI scales) and a clinician- and/or software-based speech dysarthria assessment will also be performed on day 1 (to act as baseline) and day 5 of Part II and bi-monthly in Part III.

In part IV, participant's key biomechanical parameters (arm length, shoulder width, upper body length, leg lengths etc) will be measured.

7.2. Body sensor devices (Motion capture device / Body suit; Part IV only)

The study involves 3 body sensor devices (shown in Figure 2) which are non-invasive low-bare electronic devices which will be worn by the participant:

1. The "body suit" is a black stretchable motion capture suit (manufactured by Animazoo, Brighton). The body suit contains sensors embedded in the suit which are used to detect and record movement of the entire arm and upper body whilst the participant performs scenarios.

Once wearing the suit, participants are calibrated using two defined postures (T-shape and standing to attention) via photographic record to the suit software. Participants key biomechanical parameters are also measured (arm length, shoulder width, upper body length, leg lengths etc).

A list of the possible scenarios participants will perform are detailed in Appendix 5. Scenarios will be tailored to each participant.

2. A standard commercial data glove (CyberGlove, CyberGlove Systems LLC, San Jose, CA, USA) in order to measure the position of hand joints for both the left and right hand. Participants with small hands will not be able to wear these gloves.

3. “The ETHOs” are Velcro-strap equipped body sensors that are similar to the suit in function. They can be worn over or under clothing, unlike the suit they are not connected by cables and everything operates wirelessly. This kit will be worn by the participant overnight.

Analysis will use standard techniques of Machine Based learning algorithms. Computer classifiers capable of distinguishing certain types of FRDA participants will be developed. This could aid in the early diagnosis of FRDA participants and provide information about the progress of the disease.

Guidance for participants and their carers (if applicable) will be provided.

7.3. Eye movement tasks (eye tracking; Part IV only - optional)

Eye tracking is a readily available, non-invasive technique of the ocular motor biomarkers of disease progression in FRDA. Binocular eye tracking is undertaken using a purpose developed infrared binoculary pupil tracking system (Abbott & Faisal, 2013). Participants are seated 0.7-1m from a computer controlled visual display unit and asked to observe computer-generated instructions and graphics while their eye movements are being recorded. After fitting the head-mounted device subjects are calibrated and assessed in 5 different experimental conditions. Note, condition 4 will use the same motion tracking device (ETHO) as used for overnight body motion capture to measure head movements attached (as with the body capture suit) with a headband to the patient. Participants should not wear glasses to correct for visual impairment. Contact lenses are allowed. These experiments are expected to take about 25-35 mins.

1. Saccades
Saccades are rapid re-fixation eye movements. Participants are asked to follow a target with their eyes while their head was fixed.

2. Fixation
Fixation target is presented for 60s, followed by a target-off period of 60s. A refixation flash of 100ms duration is shown at 10s intervals. Participants are instructed to look directly at the target, or at the remembered target. This sequence is repeated for a set of different target positions.

3. Smooth pursuit
The participants are asked to follow a moving target with their eyes while keeping their head as steady as possible.

4. Vestibulo-ocular reflex
The vestibulo-ocular reflex maintains steady vision during head movement. To assess VOR, the participants were instructed to stare at a target, while their head is gently, but rapidly rotated by the operator in horizontal and vertical directions.

5. Visual scene exploration
Subjects are asked to explore either a picture or a real-life scene and find a fixed number of markers within these scenes.

7.4. Balance control tasks (using a force plate; Part IV only - optional)

Participants are asked to stand (if needed supported) on a 4-sensor force-plate (Nintendo Wii Balance Board) and a visual display unit. The shifts in a subject’s centre of weight are translated to the 2D motion

of a cursor on the computer screen. Participants have to perform two tasks: 1) control from a start position the cursor so as to touch/hit a target on the unit through gentle shifts of their center of gravity. 2) hold a specified position as accurately and precisely as possible for a limited amount of time (10-60s). These experiments are only suitable for non-wheelchair bound participants and last about 5-10 mins.

Figure 2: Body sensor devices

The 'body suit'

Red and blue dots mark the approximate position of the motion capture sensors on the Animazoo IGS 150/180 textiles (top) and with respect to the body (bottom).

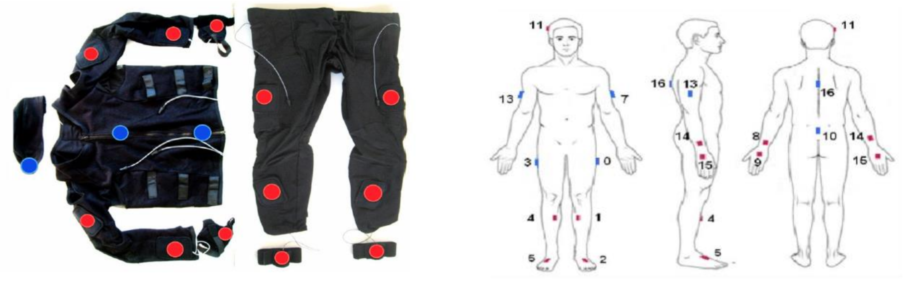

The 'data gloves':

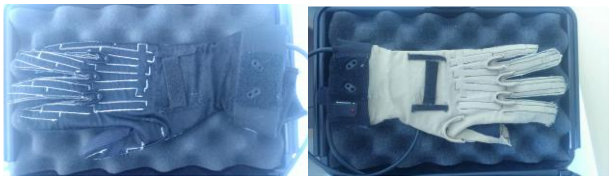

The 'ETHOs', velcro-strap equipped body sensors

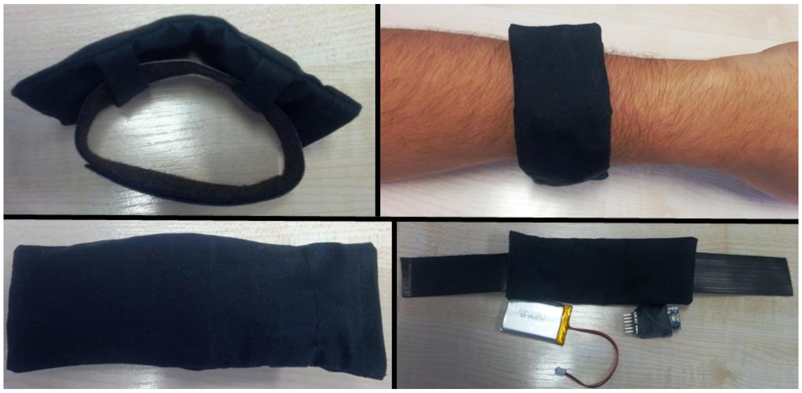

7.5. Laboratory Assessments

The below laboratory assessments will be performed:

**Haematology**	white blood cell count with differential, red blood cell count, platelet count, haemoglobin, and haematocrit
**Standard chemistry:**	
**Substrates**	albumin, total bilirubin, total cholesterol, creatinine, low-density and high-density lipoproteins, total protein, triglycerides and uric acid
**Electrolytes**	calcium, chloride, sodium, phosphorous, potassium
**Enzymes**	aspartate aminotransferase/glutamate oxalo-acetate transaminase (AST/GOT); alanine aminotransferase/glutamate pyruvate transaminase (ALT/GPT); γ-glutamyl transpeptidase (Gamma-GT); alkaline phosphatase (AP); lactate dehydrogenase (LDH); creatine kinase (CK)
**FXN level**	According to Mitosciences dipstick test and Q-RT-PCR (Parts I-IV) and LC-MS/MS (Part IV only)
**Chromatin study**	Chromatin will be extracted and stored at -80 C.<br>Spatial organisation of the FXN locus will be analysed by 3C-sequencing to correlate with FXN expression (Part IV).

Routine clinical laboratory parameters [haematology, chemistry, liver function tests (LFTs) etc] will be analysed by the local hospital laboratory (Hammersmith Hospital).

Blood glucose will be measured using a BM stick.

Pregnancy will be tested by serum for the interventional study and by urine for the non-interventional study.

7.6. Volume of Blood Collection

The approximate blood volume collected from each participant after the single dose escalation (up to 5 visits in total) and multiple dose study (over 5 days) will be as follows:

**INTERVENTIONAL STUDY**			
**SINGLE DOSE ESCALATION, PART I**			
**Screening and visits 1-4**	**Number of Collections**	**mL/ Collection**	**Total (mL)**
Clinical chemistry, including LFTs and blood glucose measured using a BM stick – at screening; at pre-dose and 24 hours post-dosing, on dosing visits	1x1 screening<br>2 x 4 visits	2.5<br>2.5	2.5<br>20
Haematology at screening; at pre-dose and 24 hours post-dosing, on dosing visits	1X1 screening<br>2 x 4 visits	2.5<br>2.5	2.5<br>20
FXN level at pre-dose and 2, 4, 8 and 24 hours post-dose	1X1 screening<br>5 x 4 visits	3*<br>3	3<br>60
Chromatin at screening	1x1 screening	40	40
**Sub-total blood volume:**			**Up to 148 ml****

*6mls will be taken for participants who will directly enter into part III

Visit 5 of the single dose escalation study will enable the effect of nicotinamide on chromatin structure and will therefore give important information regarding the mechanism of action in upregulating FXN. Participants will be given either the MTD, 8g of nicotinamide or the dose determined by escalation to be effective in upregulating FXN.

**Assessment by visit: Visit 5**	**Number of Collections**	**mL/ Collection**	**Total (mL)**
Clinical chemistry, including LFTs and blood glucose measured by BM stick at pre-dose and 24 hours post-dosing	2	2.5	5
Haematology at pre-dose and 24 hours post-dosing	2	2.5	5
FXN level at pre-dose and 2, 4, 8 and 24 hours post-dose	5	3	15
Chromatin immunoprecipitation at pre-dose and 8 hours post-dosing	2	40	80
**Sub-total blood volume:**			**≈ 105 ml**
**MULTIPLE DOSE, Part II**<br>**5 consecutive days**			
**Assessment by day**	**Number of Collections**	**mL/ Collection**	**Total (mL)**
Clinical chemistry, including LFTs and BM measured by stick –daily	5	2.5	12.5
Haematology – daily	5	2.5	12.5
FXN level at pre-dose and 2, 4, 8 post-dose– daily	5x4=20	3	60
Chromatin immunoprecipitation at pre-dose on day 1 and on day 5.	2	40	80
**Sub-total blood volume:**			**≈ 165 ml**
**MULTIPLE DOSE, Part III**<br>**Weekly visits over two months**			
**Assessment by visit**	**Number of Collections**	**mL/ Collection**	**Total (mL)**
Clinical chemistry, including LFTs and BM measured by stick –weekly	8	2.5	20
Haematology - weekly	8	2.5	20
FXN level – pre-dose, weekly. (If necessary also at 8hrs post-dose)	16	3	48
Chromatin immunoprecipitation end of every month	2	40	80
**Sub-total blood volume:**			**168 ml**
**END OF INTERVENTIONAL STUDY**			
	**Number of Collections**	**mL/ Collection**	**Total (mL)**
Clinical chemistry, including LFTs and BM measured by stick	1	2.5	2.5

Haematology	1	2.5	2.5
FXN level	1	3	3
Chromatin immunoprecipitation	1	40	40
**Sub-total blood volume:**			**≈ 48 ml**
**Total blood volume for the interventional study (Screening, Parts I, II and III and End of Interventional Study Visit):**			**Up to 634ml***

*Up to 267mls will be taken for participants who will directly enter into part III
Up to 429mls will be taken for participants who participate in Parts II and III but not in Part I.

**NON- INTERVENTIONAL STUDY**			
**Part IV**			
**Assessment by visit**	**Number of Collections**	**mL/ Collection**	**Total (mL)**
FXN level (Mitosciences dipstick test and LC-MS/MS)	4	12	48
Biomarkers	4	5	20
Spatial organisation of the FXN locus	4	35	140
**Sub-total blood volume:**			**208 ml**
**Total blood volume for both the interventional and non-interventional study (Screening, Parts I, II, III, IV and End of Interventional Study Visit):**			**842 ml***

*Up to 475mls will be taken for participants who will directly enter into part III and will also participate in part IV of the study.
Up to 637mls will be taken for participants who participate in Parts II, III and IV but not in Part I.

7.7. Prior and Concomitant Medications

Medications (prescription, over-the-counter and herbal) and nutritional supplements taken during the 30 days prior to dosing and medication taken for the treatment of FRDA during the 8 weeks prior to dosing will be reviewed and recorded at screening. At the first baseline visit, current medications will be recorded. At each subsequent visit, or early discontinuation visit (if applicable), the change in medications since the previous visit will be recorded.

In part III, participants will be instructed to complete a weekly trial diary until the end of interventional study visit. Participants will record any concomitant medications taken during this period.

7.8. Screening and Baseline

Screening procedures (unless otherwise specified below) must be performed no more than 28 days prior to the first dose of study drug. Prior to performing any study-related assessments, the CI or delegates will inform the participant about the potential benefit and any risks associated with the participation in this trial. Participants and their GPs will be given an information sheet and given as much time as they wish to read and ask questions prior to giving informed consent.

After having given their informed consent, participants will undergo the following screening evaluations:

- General medical and drug history, including recording of demographic data; concomitant diseases; review of medication(s) or investigational products taken or investigational devices

used in the past 30 days and medications taken for the treatment of FRDA in the 8 weeks prior to enrollment; and complaints/symptoms for baseline safety evaluation.
- Physical examination (including body weight and height) and evaluation of disabilities.
- Participants who will enter Part III and not Part II of the study will also be assessed using the FARS, SCAFI and speech dysarthria assessment.
- Blood samples will be taken for standard clinical laboratory tests (haematology, chemistry and LFTs), BM (measured by stick) and FXN level and baseline chromatin studies. Women of childbearing potential will also be tested for pregnancy.
- Urinalysis.
- Vital signs (BP, temperature and heart rate).
- ECG.

Part IV:
Participants may be contacted by telephone and / or email by the CI or delegates to inform them about the potential benefit and any risks associated with participation in Part IV. fMRI contraindications will be detailed. Participants will be given the PIS and given as much time as they wish to read and ask questions. If the participant wishes to take part, informed consent will be given prior to any study-related assessments being performed. Participants will be assessed for fMRI contraindications (including urine pregnancy test for women of childbearing potential).

7.9. Single Dose Escalation Visits

At each visit, participants will return to the Imperial CRF to receive treatment with nicotinamide. They will be admitted to the Imperial CRF and the following assessments will be performed:
- Vital signs assessment (pulse rate, blood pressure, respiration rate, pulse oximetry and temperature), these will be repeated prior to each blood sample being taken (see below).
- Assessment and recording of AEs since baseline throughout the visit
- Review of concomitant medications
- If participants experience nausea they will be given a VAS to rate the severity.
- Each participant will be given an oral dose of nicotinamide (as detailed in schema).
- Blood samples taken for standard clinical laboratory tests (haematology, chemistry, and LFTs), blood glucose will be measured using a BM stick at predose and 24 hours following nicotinamide administration.
- Blood samples to assess FXN level will be taken predose and at 2 (± 15 mins), 4 (± 25mins), 8 (± 1) and 24 (± 2.5) hours following nicotinamide administration.
- On the last dosing visit blood samples for the separate Chromatin immunoprecipitation studies will be taken at pre-dose and at 8 (± 1) hours post-dosing.
- Participants will spend the day within the Imperial CRF and return home after the last blood test. They will return the following day for the 24 hour blood test. In some cases, if travel is difficult, participants may be offered overnight accommodation either at the Imperial CRF or private accommodation.
- All participants will be reviewed by the CI or delegates prior to discharge.

7.10. Multiple dose study

Part II
The multiple dose study is similar to the single dose study and will commence after a washout interval of at least one week.
- Participants will be admitted for four nights within the Imperial CRF and return home after the last blood test on day 5. It may be possible in certain exceptional circumstances for some participants to return home every evening. This would need to be agreed by the CI.

- The ratio of participants that will be 'on no nicotinamide' to those on 'on nicotinamide' will be 1:4 (i.e one patient will be on 'no treatment' to 4 'on treatment'). Participant's allocation to no drug will be determined at the CI's discretion and discussed with the participant. The daily dosing regimen may be modified at the CI's discretion. The daily dosing regimen may be modified from once to twice a day for some participants at the CI's discretion.
- Vital signs assessment (pulse rate, BP, respiration rate, pulse oximetry and temperature) will be repeated prior to each blood sample being taken.
- ECG will be taken predose on day 1 and prior to discharge on day 5.
- Assessment and recording of AEs from baseline throughout the visit
- Review of concomitant medications
- In order to avoid nausea, participants will be administered anti-nausea treatment if required (domperidone, 20 mg oral, metoclopramide etc) prophylactically pre-dose and post-dose (domperidone, 20 mg oral up to four times daily; metoclopramide 10-20 mg IV PRN up to three times daily etc).
- If participants experience nausea they will be given a VAS to rate the severity.
- Blood samples for clinical chemistry, haematology will be taken daily immediately prior to dosing.
- Blood samples to assess FXN level will be taken daily pre-dose and at 2 (± 15 mins), 4 (± 25mins), 8 (± 1) hrs following nicotinamide administration. The pre-dose sample will also represent the 24 hour sample of the previous day. The last 24 hour sample will be taken on day 4. On the last day, the last 8hr PK sample may be taken (±2)hrs as the Imperial CRF closes at 6pm on Fridays.
- Further blood samples for the chromatin immunoprecipitation will be taken at pre-dose on day 1 and day 5.
- Participants and their carers will complete Part II of FARS. Neurological assessment (SARA and SCAFI scales) and a clinician- and/or software-based speech dysarthria assessment will also be performed on day 1 and day 5.
- All participants will be reviewed by the CI or delegates prior to discharge.
- Prior to discharge participants will be provided with the study drug supply and rescue medication (typically domperidone or metoclopromide) to take if required prophylactically pre-dose (domperidone, 20 mg oral) and post-dose (domperidone, 20 mg oral) up to four times daily when necessary until the day of the first visit of Part III is scheduled (if applicable).

Part III

If the multiple dose dosing regimen of nicotinamide in Part II is well tolerated and changes in FXN level are observed, all participants (including those who were scheduled to receive no drug in part II) will be offered to enter Part III. Participants who participated in part I but not in part II of the study will be allowed to participate. These participants may require re-screening. This decision will be taken by the CI. If required, additional participants will undergo screening and if eligible may enter directly into this part of the study.

Participants who were 'on drug 'in Part II will take their MTD already established over the course of two months. If any AEs occur that in the CI's opinion may put the safety of the participant at risk, the dose schedule will be adjusted. If a participant was on 'no drug' in Part II or due to enter Part III directly they will start at a low dose of nicotinamide (typically 2g) which will be gradually escalated in the absence of any AEs which in the CI's opinion may put the participant's safety at risk.

Participants who took part in Part II will be given the weekly drug supply and diary on Part II Day 5. Participants who took part in Part I but not Part II or participants who will directly enter Part III will be given the weekly drug supply and diary at their screening visit (only once determined eligible) or by courier / recorded delivery post. Their next visit will be Part III, week 1.

If the participant stops taking nicotinamide for several days (for safety reasons), only once the participants AEs are fully resolved. The CI may decide to add this drug interruption period onto the end of the two month period.

To ensure that sustained FXN upregulation is achieved, the nicotinamide PK/PD relationship [i.e. time to nicotinamide steady state vs. upregulation of FXN) will be evaluated at each weekly visit and the daily dose regimen may be modified from once to twice a day [i.e. from 6 grams (or MTD) OD to 3 grams (or ½ MTD) BD]. Participants will come to the Imperial CRF prior to taking their dose of nicotinamide once a week (± 2 days) over this time period.

In order to ensure compliance, participants will be instructed to complete a weekly trial diary for the entire duration of Part III until the end-of-study visit. The first weekly trial diary will be given to the participant prior to discharge on day 5 of Part II or at screening or recorded delivery post or courier if participants are participating in Part III only. Participants will record any AEs and concomitant medications from the next day until their next visit.
Each week, the trial diary from the previous visit will be collected and participants will be provided with another diary to complete starting on the next day. Participants will be provided with anti-nausea treatment as rescue medication; they will be asked to keep an accurate record of use in the trial diary.

At each visit:

- Participants will come to the Imperial CRF and return home after dosing.
- Vital signs assessment (pulse rate, BP, respiration rate, pulse oximetry and temperature), these will be taken predose.
- ECG will be taken predose and if necessary before discharge (in cases where the participant will be staying for 8hrs post-dose).
- Assessment and recording of AEs since baseline throughout the visit
- Review of concomitant medications
- Blood samples taken for standard clinical laboratory tests (haematology, chemistry, and LFTs), blood glucose will be measured using a BM stick at predose.
- Blood samples to assess FXN level will be taken predose and if necessary at 8hrs post-dose.
- Participants will be given a trial diary.
- All participants will be reviewed by the CI or delegate prior to discharge. Participants will be provided with the study drug supply and rescue medication (if required) until the day of their next scheduled visit. Rescue medication (anti-nausea treatment, typically domperidone or metoclopromide) should be taken if required prophylactically pre-dose (domperidone, 20 mg oral) and post-dose (domperidone, 20 mg oral up to four times daily when necessary).

The following assessment will be performed on bi-monthly visits (visits 2, 4, 6 and 8):

- Participants and their carers will complete Part II of FARS bi-monthly. Neurological assessment (SARA and SCAFI scales) and a clinician- and/or software-based speech dysarthria assessment will also be performed.

The following assessment will be performed on monthly visits (visits 4 and 8):

- Blood samples for the separate chromatin immunoprecipitation studies will be taken predose.

7.11. End of Interventional Study Follow-up Visit

After at least a 1 week and at most 8 weeks washout interval for the interventional study participants will return to the Imperial CRF for routine medical assessment and the following safety assessments will be performed:

- Review concomitant medications
- AE assessment

- Physical examination including vital signs (pulse rate, BP, respiration rate, pulse oximetry and temperature) and assessment of level of disability, using FARS, SARA, SCAFI and speech dysarthria assessment.
- 12-lead ECG in the supine position
- Blood and urine samples will be taken for clinical laboratory assessment (haematology, clinical chemistry, electrolytes, coagulation, fasting lipid profile, enzymes and urinalysis). Blood glucose will be measured using a BM stick. A blood sample for FXN level will also be taken.
- A blood sample for the separate chromatin immunoprecipitation study will be taken.

Participants will be discharged from the interventional study when considered appropriate by the CI.

We do not recommend that participants continue to take nicotinamide post-study as further clinical trials are required to determine whether or not nicotinamide is safe and is of clinical benefit to patients with FRDA. If participants still decide to take nicotinamide against our advice, they will referred to their GP with recommendation of regular safety follow up to evaluate any nicotinamide-induced toxicity. We may request the FRDA participants consent to come in for visits at the Imperial CRF at the CI's discretion. Blood tests (FXN/chromatin, safety bloods etc) and neurological and medical examinations (SARA, SCAFI, ADL, ECG, vital signs etc) may be performed at these visits.

If the interventional project is successful, FRDA participants may be approached for future research in the same area. It will be entirely up to the participant to decide whether to consent to participation in any future interventional research study.

7.12. Part IV

A minimum of ten up to a maximum of 20 FRDA participants will be offered the opportunity to participate in Part IV. Up to 20 HVs will also be recruited to participate in this part of the study.

Participants may be contacted by telephone and/or email by the CI or delegates or at their study visit (i.e FRDA participants participating in the interventional study) to inform them about the potential benefit and any risks associated with participation in this part of the study. fMRI contraindications will be detailed. Participants will be given the PIS and given as much time as they wish to read and ask questions. If the participant wishes to take part, informed consent will be given in writing prior to any study-related assessments being performed. Participants will be assessed for fMRI contraindications during screening (including urine pregnancy test for women of child-bearing potential prior to fMRI) prior to study start.

FRDA participants will undergo the below following assessments over the course of 2 days at the study start, 2 weeks (± 2 days), 3 months (± 2 weeks) and at 9-12 months (± 3 months). In some cases if travel is difficult, participants will be offered overnight accommodation (at the Imperial CRF or private hospital accommodation).

1 Urine pregnancy test only for women of child-bearing potential (prior to fMRI).
2. Blood samples will be taken for:
3. FXN protein estimation by LC-MS/MS compared to the current dipstick assay (Mitosciences);
4. RNA-seq to identify informative biomarkers that might track FXN levels and
5. Analysis of the spatial organisation of the FXN locus by Chromosome-Conformation Capture coupled with high-throughput sequencing (3C-sequencing) to correlate with FXN expression – such a technique may be capable of identifying participants that would be predicted to respond to epigenetic therapies such as nicotinamide.
6. The Edinburgh handedness score will be performed at the study start visit only.
7. Participants will perform standardised tasks derived from the SARA and SCAFI

8. fMRI assessment during the execution of a standardised finger-tapping task (Akhlaghi et al., 2012) and object manipulation. fMRIs will be performed at the Imperial College Centre Imaging Facility (CIF). Participant's hands will be filmed on an fMRI compatible device, which analyses movements in real-time and will be correlated with the fMRI analysis.
9. Participants will be fitted with a simple portable motion-capture device similar to an ECG to be worn overnight. This will be returned the following day.
10. Participants will perform standardised ADL tasks (Appendix 5) and the SARA and SCAFI (not the timed speech test) while wearing a full-body, motion-tracking equipment ('body suit') to capture detailed body kinematics.
11. Participants will perform standardised eye movement tasks to assess the performance of their occulo-motor system quantitatively assessed using a binocular eye-tracking system (optional).
12. Participants will stand on a force plate and their balance performance will be measured (optional).
13. Chromatin immunoprecipitation studies as for Parts II and III to determine longitudinal changes and correlate with FXN expression and clinical phenotype.

Healthy volunteers will come over the course of several visits (not constrained to occur at any point in time, since change in motor behavior is not expected to change significantly for HV during the course of the study) and will perform the following assessments:

1. Urine pregnancy test for women of child-bearing potential only (prior to fMRI).
2. The Edinburgh handedness score.
3. fMRI assessment during the execution of the standardised finger-tapping task (Akhlaghi et al., 2012) and object manipulation. Participant's hands will be filmed on an fMRI compatible device, which analyses movements in real-time and will be correlated with the fMRI analysis.
4. Naturalistic tasks while wearing a motion capture suit and gloves. These include, but are not limited to, naturalistic scenarios (such as "Breakfast" or "Office" scenarios), as well as Repeated Identical Daily Standard Tasks and Clinical Assessments with both the SARA and SCAFI scales.
5. Standardised eye movement tasks to assess the performance of their occulo-motor system quantitatively assessed using a binocular eye-tracking system (optional).
6. Balance performance measurements, by standing on a force plate and performing simple balance control tasks (optional).

8. SAFETY

This will be the first use of nicotinamide in participants with FRDA. However, the study is designed with safety and tolerability as outcome measures and draws upon expertise from academia and industry in early phase studies. Nicotinamide will be obtained from an accredited manufacturing source (Teofarma). The safety issues are nausea and isolated reports of reversible abnormal liver function (Knip et al., 2000). Nausea will be monitored throughout by use of a VAS and LFTs measured throughout. A large number of studies have administered nicotinamide at high dose for prolonged periods (Knip et al., 2000). There have been several PK studies where doses of between 6g/day and 9g/day were given to normal volunteers with minimal side effects when given in tablet form (Stratford et al., 1996; Stratford et al., 1992).

Out of a very large number of participants given high-dose nicotinamide only one case of hepatotoxicity has been reported as potentially attributable to prolonged high-dose nicotinamide administration (9g/day) – this was reversible on withdrawal of the drug. No other SAEs have been reported despite high-dose administration for prolonged periods.

8.1. Reference Safety Information for nicotinamide

Maximum tolerated dose is the dose above which AEs, likely to be nausea (leading to uncontrolled vomiting), become intolerable and there are biochemical indications of hepatotoxicity (AST, ALT or Bilirubin >25% above the normal range). Nausea will be assessed by VAS (from 0-10). Clearly, if it is severe

enough to cause vomiting which is not relieved by anti-nauseants in more than 50% of participants this will lead to cessation of dose escalation. Overall, the MTD would be exceeded when any of the volunteers had experienced the occurrence of an unexpected, significant or unacceptable AE such that the continuation of the study would put their or other trial participants' safety at risk.

Side effects of nicotinamide and available data on their frequency when using high doses has been reviewed recently by Knip et al (Knip et al., 2000) and are shown in the table below.

Table 2 Nicotinamide side effects

**Side-effects**	**Reported frequency**
Flushing	≤ 1.5%
Facial erythema	≤ 0.5%
Hives	≤ 0.4%
Sore mouth	≤ 0.4%
Dull headache	≤ 0.5%
Heartburn	≤ 1.6%
Nausea (with radiotherapy)	17-65 %
Nausea (without radiotherapy)	≤ 1.5%
Other gastrointestinal symptoms	≤ 0.8%
Inability to focus the eyes	≤ 0.4%
Dry hair	≤ 0.4%
Fatigue	≤ 0.4%

In addition, further potential effects are hypersensitivity to the nicotinamide preparation used in this study i.e. Nicobion.

8.2. Adverse Event Terminology / Definition

An AE is "any untoward medical occurrence in a participant to whom a medicinal product has been administered, including occurrences which are not necessarily caused by or related to that product". An AE can therefore be any unfavorable and unintended sign (including an abnormal laboratory finding), symptom, or disease temporally associated with the use of an investigational medicinal product (IMP), whether or not considered related to the IMP.

AEs include any of the following:

- Worsening (change in nature, severity or frequency) of conditions present at the onset of the trial
- Participant deterioration due to the primary illness
- Inter-current illnesses
- Drug interactions
- Events related or possibly related to concomitant medications
- Abnormal laboratory values or changes of vital signs, as well as significant shifts from baseline within the range of normal, which the CI or delegates considers to be clinically significant (CS)

8.3. Adverse Drug Reaction

In the pre-approval clinical experience with a new medicinal product or its new usage, particularly as the therapeutic dose(s) may not be established, an adverse drug reaction (ADR) is defined as: 'Any untoward and unintended response in a participant to an IMP which is related to any dose administered to that participant.

8.3.1. Unexpected Adverse Drug Reaction

An unexpected ADR is an ADR, the nature or severity of which is not consistent with the applicable product information, also known as reference safety information (RSI). The RSI for nicotinamide is found in section 8.1.

8.3.2. Suspected Unexpected Serious Adverse Reactions

(SUSARs) are defined as any serious adverse reactions that are unexpected i.e. it is not consistent with the information about the study drug provided in the Summary of Product Characteristics (SmPC) /RSI, and for which there is a reasonable possibility that the event may be caused by the IMP(s).

8.4. Serious Adverse Event/Serious Adverse Drug Reaction

During clinical investigations, serious AEs (SAE) may occur. If the event is suspected to be drug-related, the event may be significant enough to lead to important changes in the way the medicinal product is developed (e.g. change in dose, population, needed monitoring, consent forms). This is particularly true for reactions, which, in their most severe forms, threaten life or function.

An SAE or serious adverse drug reaction (serious ADR) is any untoward medical occurrence that:

- Results in death.
- Is life-threatening. 'Life-threatening" refers to an event in which the participant is at risk of death at the time of the event; it does not refer to an event which hypothetically might have caused death if it were more severe (ICH E6).
- Requires in-participant hospitalisation or prolongation of existing hospitalisation.
- Results in persistent or significant disability/ incapacity (as per reporter's opinion).
- Is a congenital anomaly/birth defect.
- Is another medically important condition. Important medical conditions that may not result in death, be life-threatening or require hospitalisation may be considered as SAEs or SADRs when, based upon appropriate medical judgment, they may jeopardize the participant or may require intervention to prevent one of the outcomes listed in the definition above. Examples of such events are intensive treatment in an emergency room or at home for allergic bronchospasm; blood dyscrasias or convulsions that do not result in hospitalisation; or development of drug dependency or drug abuse.
- Please note: Serious is not synonymous with severe. An event may be severe (e.g., severe headache) but still be of minor medical significance. Serious refers to an event that poses a threat to the participant's life or functioning.

8.5. Assigning Severity to an Adverse Event

- Mild: Causing no limitation of usual activities; the participant may experience slight discomfort.
- Moderate: Causing some limitation of usual activities; the participant may experience annoying discomfort.
- Severe: Causing inability to carry out usual activities; the participant may experience intolerable discomfort or pain.

- Life threatening or disabling: Immediate risk of death from the reaction as it occurred.
- Death: The event resulted in death.

8.6. Assigning Causality of the Adverse Event to the IMP

The CI or delegates will determine the relationship of each AE to study drug (i.e., causality) by using the classification criteria: 'related', 'probably related', 'possibly related' 'unlikely to be related' or 'not related'. If an AE is classified as either 'possibly related' or 'probably related', it should be considered to be an ADR. Descriptions of the six classification categories are as follows:

8.6.1. Not Related

Exposure to the IMP has not occurred; administration of IMP and the AE are not reasonably related in time; or the AE is considered by the CI or delegates to be due to a pre-existing condition, a known manifestation of the target disease, a recurrent condition, or is likely explained by environmental or diagnostic therapeutic factors or was pre-existing and did not deteriorate.

8.6.2. Possibly Related

The AE occurred during or within a reasonable period of time after administration of the IMP, or a pre-existing event worsened within an appropriate period of time after administration of the IMP, but the AE could be explained equally well by factors or causes other than exposure to the IMP.

This category will also be used if there is a lack of information, or insufficient or conflicting evidence exists for classifying the causality of the AE.

8.6.3. Probably Related

The AE occurred during or within a reasonable period of time after administration of the IMP or a pre-existing event worsened within an appropriate period of time after administration of the IMP, and at least one of the following criteria is applicable:

- the event could not be explained by the clinical condition or history of the participant, environmental or toxic factors, or other diagnostic or therapeutic measures;
- the event was an expected ADR associated with study treatment or a class-labeled drug effect;
- the AE subsided or disappeared after withdrawal or dose reduction of study treatment; or
- the AE recurred after re-exposure to study treatment.

8.6.4. Related

There is clear evidence to suggest a causal relationship of the AE with the study drug and other possible contributing factors can be ruled out.

8.6.5. Unlikely to be related

There is little evidence to suggest there is a causal relationship of the AE with the study drug (e.g. the event did not occur within a reasonable time after administration of the study drug). There is another reasonable explanation for the event (e.g. the participant's clinical condition, other concomitant treatment).

8.6.6. Not assessable

There is insufficient or incomplete evidence to make a clinical judgment of the causal relationship.

8.7. Adverse Event Recording and Reporting

8.7.1. Adverse Event Recording

All AEs will be recorded throughout the study from 1st IMP administration until the end of follow-up.

Each AE occurring to a participant, either spontaneously revealed by the participant or observed by the CI or delegates, whether believed by the CI or delegates to be related or unrelated to the study drug, must be recorded on the eCRF. Type and severity of AEs will be reported by the participants without being given a list of fixed symptoms beforehand.

The CI or delegates will also determine causality of any AE to the IMP and record it on the eCRF as well as their severity, time of onset, duration, and the precautions carried out, and whether or not the event meets one or more of the definitions of an SAE.

Laboratory results will be recorded on the eCRF and the CI or delegates will indicate whether abnormal results (high or low) are clinically or not clinically significant (CS or NCS). Clinically significant laboratory abnormalities, CS changes in vital signs (e.g., tachycardia) or other CS changes observed by the CI or delegates will be entered onto the eCRF.

8.7.2. Serious Adverse Event Reporting

All SAEs must be reported promptly to the CI.

All SAEs will be reviewed by the CI or delegates to confirm relatedness and expectedness. All SAEs have to be reported, whether or not considered causally related to the IMP, or study procedure(s).

It is the CIs responsibility to report all SAEs to the sponsor, regulatory authority and REC (if applicable).

At the time of the initial report, the CI or delegates will fill in an SAE Reporting Form comprising but not limited to the following information:

- Participant demographics
- Protocol number
- IMP (drug name, date of dose, dose etc).
- Nature of the SAE including date and time of onset and resolution, severity and treatment (including hospitalisation)
- Action taken with respect to the IMP
- Relationship of the AE to the IMP, in the opinion of the CI or delegates.
- Concomitant drug therapy at the time of the SAE
- Outcome (if available)
- Recovery date (if available)
- In the case of death, the cause and post-mortem findings (if available).

This form together with any other anonymised copies of all relevant investigations will be sent to the sponsor.

Follow up information about a previously reported SAE must be reported within 24 hours of receiving it. These adverse events will be followed up until resolved or stabilised at a level acceptable to the CI even if this is after the study reporting period.

All SAEs will be recorded in the eCRF as soon as is possible.

8.7.3. Reporting to the Chief Investigator

The Study Team must report any SAE to the CI (in person, by telephone, or by fax) within 24 hours after becoming aware of the SAE.

Follow-up reports relating to the participant's subsequent course must be submitted as necessary by the study team to the CI until the event has subsided or the condition has stabilised or resolved at a level acceptable to the CI even if this is after the study reporting period..

For notification of SAEs, please contact:
Professor Richard Festenstein, MB BS PhD FRCP
Email: r.festenstein@imperial.ac.uk

Inside UK	Outside UK
Phone: 0208-383-8310	Phone:+44-208-383-8310
Fax: 0208-383-8306	Fax: +44-208-383-8306

8.7.4. Reporting to Sponsor

All SAEs and SUSARs must be forwarded to the Sponsor (Imperial College Joint Research Compliance, JRCO Office) within 24 hours of the CI learning of its occurrence.

Imperial College Joint Research Compliance Office
Email address: jrco.ctimp.team@imperial.ac.uk
Fax number: 0203-311-0203

8.7.5. Reporting to the Regulatory Authority and Research Ethics Committee

The Sponsor has delegated the responsibility for notifying the MHRA and REC of all SUSARs occurring during the study to the CI. All SAEs and SUSARs must be reported in accordance with local regulatory guidelines:

Life threatening SUSARs should be reported to the MHRA and REC no later than **7 days** after the CI has first knowledge of the minimum criteria for expedited reporting. Further relevant information should be given within a further 8 days.

Non-fatal and non-life threatening SUSARs should be reported to the MHRA and REC no later than **15 days** after the CI has first knowledge of the minimum criteria for expedited reporting. Further relevant information should be given as soon as possible.

An annual Developmental Safety Update Report (DSUR) will be submitted on the anniversary of the Clinical Trial Application to the MHRA and REC.

9. DOCUMENTATION OF DATA

The data collection tool for this study will be electronic Case Report Forms (eCRFs). Electronic Case report Forms will be completed for each participant that received the IMP and will contain study data which are verifiable to the source data (i.e., participant's source worksheets, medical records, original recordings, laboratory reports etc). All source data will be attributable (initialed) and quality checked (signed and dated). Only the CI and delegates are authorised to make entries on the eCRF.

Concomitant medications may be entered as they appear in the participant's record or as per local standards (Generic or Trade names).

It is the responsibility of the CI and delegates to ensure that the eCRF is kept up-to-date.

Other data than those requested by this protocol may be recorded as "additional data" in the comments section of the eCRF; the clinical significance of any additional data should be described.

10. EVALUATION AND STATISTICS

Taking into account the variability in FXN levels obtained in the studies on primary cells and mice this study is designed to enable within-participant comparisons and will allow the calculation of fold-change in FXN level at the level of RNA and protein compared to baseline using standard statistical tests and correlation coefficients applied to dose-response curves. This will determine if further participants should be enrolled in dose escalation studies and will allow power calculations for a further double-blind cross over study if the present study demonstrates upregulation of FXN.

11. ADMINISTRATIVE AND LEGAL CONSIDERATIONS

To ensure compliance of this trial with GCP principles, the following procedures will be followed by the CI and delegates.

11.1. Pre-Trial Requirements

The CI and study team must be well informed about the properties of the IMP, especially by the current version of the SmPC.

Treatment within this study will only start if the following prerequisites have been fulfilled and documentation is at hand:

- Signed copy of the approved protocol
- Sponsor, REC, MHRA and Trust approval of the protocol, PIS and consent form

The CI or delegates will initiate and maintain an Investigator's Trial Master File (iTMF) containing all relevant study documentation and approvals.

11.2. General Legal Requirements

The study will be conducted in agreement with the following directives and guidelines:

- the Declaration of Helsinki (version of Edinburgh, Scotland, October 2000)
- the respective Guidelines of the European Community: Guideline for Good Clinical Practice (Note for Guidance on Good Clinical Practice / ICH E6) and
- Statutory Instrument 2004 No. 1031 - The Medicines for Human Use (Clinical Trials) Regulations 2004 as amended.

All clinical work conducted under this protocol will be subject to GCP rules.

The CI agrees to the inspection of study-related records by health authority representatives and/or the sponsor at any time.

11.3. Protection of Participants

11.3.1. Research Ethics Committee / Medicines Healthcare Regulatory Authority

Written approval of the protocol, PIS and consent form by the MHRA and REC will be obtained prior to the start of the trial.

11.3.2. Informed Consent

It is the CI's or delegates responsibility to explain to each participant the study procedure, potential benefits and hazards of trial participation, the right to withdraw from the study at any time, and to obtain informed consent prior to any study-specific procedures. Participants must provide informed consent. The original copy of the signed and dated Informed Consent Form will be filed in the iTMF. If written consent is not possible due to physical incapacity, written consent on behalf of the participant will be sought from the participant's relatives or carer. A copy of the informed consent form will be placed in the participant's medical notes and a copy given to the participant.

11.3.3. Regulations

The CI and study team will conduct the trial in full conformity with the Declaration of Helsinki, and/or with the national or local laws and regulations, whichever affords the higher protection of the participant.

11.3.4. Privacy protection

The CI and study team affirm and uphold the principle of the participant's right to protection against invasion of privacy. Personal health data will be kept confidential.

On the study specific source worksheets and eCRFs participants will be identified by their initials, screening number and a participant number only. A Subject Identification List will be held in the iTMF.

11.4. Amendments

Amendments will be approved by the CI and Sponsor.

Substantial amendments will be submitted to the REC and the MHRA (if applicable) and Joint Research Compliance Office for approval prior to implementation.

Non-substantial amendments which have no significant impact on the medical or scientific validity of the study will be documented. The REC may be notified, if required.

11.5. Premature Termination of the Trial

The CI reserves the right to terminate the trial for well-documented reasons. Instructions will be provided in a separate document should it be determined that assessments beyond those defined by the protocol are required.

Further recruitment of participants will not take place under the following conditions:

- Premature termination of the trial.
- Drug-related events, i.e. SUSARs, emerging adverse effects that are serious and the risk/benefit ratio is unacceptable.
- Procedure-related events, i.e., the recruitment rate is too low or the number of dropouts for administrative reasons is too high.

An End of Interventional Study Visit will be completed for each participant who completes the interventional study or who withdraws prematurely from this study.

11.6. Sponsor's / Chief Investigator Responsibilities

Responsibilities of the sponsor delegated to the CI include but are not limited to:

- Be thoroughly familiar with the properties of the IMP
- Submit an up-to-date curriculum vitae and other credentials where required to relevant authorities
- Agree to and sign the protocol and confirm that they will personally conduct or supervise the study according to the protocol and GCP
- Nominate (if appropriate) a Clinical Project Manager, Lead Research Nurse and/or Co-Investigator(s) to assist in the administration of the trial
- Submit notification/application to the relevant authorities as appropriate
- Submit notification/application to relevant bodies including local hospital management and to REC as appropriate
- Provide information to all study team members
- Fully inform trial participants about the clinical trial and obtain their informed consent
- Certify that the IMP has been correctly delivered, stored and safely handled, and that stock can be reconciled.
- Manage IMP delivery and documentation with care
- Collect, record, and report data properly
- Notify MHRA and REC in the case of serious unexpected SUSARs as specified in section 8.6.2.3. and at all times to take appropriate measures to safeguard participants
- Ensure that the confidentiality of all participant information is respected by all persons involved.
- Make all data available to relevant Authorities for validation/ audit/ inspection purposes
- Ensure that medical records are clearly marked to indicate that the participant is participating in a clinical trial

11.7. Record Retention

The CI must retain all study records by the applicable regulations in a secure and safe facility. The institution must consult with the CI before disposal of any study records, and must notify the CI of any change in the location, disposition or custody of the study files. The CI/Sponsor must take measures to prevent accidental or premature destruction of essential documents, that is, documents that individually and collectively permit evaluation of the conduct of a study and the quality of the data produced, including paper copies of study records (e.g., source worksheets) as well as any original source documents that are electronic as required by applicable regulatory requirements.

The CI/Sponsor should retain participant identifiers for at least 5 years after the completion or discontinuation of the study. Participant files and other source data must be kept for the maximum period of time permitted by the hospital, institution or private practice, but not less than 5 years. These documents should be retained for a longer period, however, if required by the applicable regulatory requirements. The CI must be notified and will assist with retention should institution be unable to continue maintenance of participant files for the full 5 years. It is the responsibility of PI to inform the institution as to when these documents no longer need to be retained.

If an Investigator moves, withdraws from an investigation, retires, requests to move records to another location or to assign these records to another party or (e.g. other Investigator) who will accept the responsibility, written notice of this transfer must be made to and agreed upon by each party.

The originals of the protocol and the Drug Accountability List will be archived by the CI.

11.8. Confidentiality

All information concerning this study and which was not previously published is considered confidential information and shall not be used except in the performance of this study.

11.9. Study Monitoring

The Sponsor is responsible for ensuring that the study is monitored appropriately in order to ensure compliance with GCP and local regulatory guidelines. The monitor will check the completeness of medical records, verify the accuracy of entries in the eCRF, and ensure adherence to the protocol and compliance with local regulatory requirements.

11.10. End of Clinical Trial Report

An end of clinical trial form will be prepared under the responsibility of the CI and submitted to the Sponsor, REC and MHRA.

11.11. Publications

Publications are the responsibility of CI.

11.12. Audits and Inspections

To ensure compliance with GCP and all applicable regulatory requirements, the sponsor may conduct a quality assurance audit in accordance with their local internal procedures.

Audits and/or inspections may also be carried out by local authorities, or authorities to which information on this trial has been submitted. All documents pertinent to the trial must be made available for such inspection after an adequate announcement.

REFERENCES

Al-Mahdawi, S., Pinto, R.M., Ismail, O., Varshney, D., Lymperi, S., Sandi, C., Trabzuni, D., and Pook, M. (2008). The Friedreich ataxia GAA repeat expansion mutation induces comparable epigenetic changes in human and transgenic mouse brain and heart tissues. Hum Mol Genet 17, 735-746.

Abbott, W.M. and Faisal, A.A. "Ultra low-cost 3D gaze estimation: an intuitive high information throughput compliment to direct brain-machine-interfaces", J.Neural Eng. 9(4):046016

Burk, K., Malzig, U., Wolf, S., Heck, S., Dimitriadis, K., Schmitz-Hubsch, T., Hering, S., Lindig, T.M., Haug, V., Timmann, D., et al. (2009). Comparison of three clinical rating scales in Friedreich ataxia (FRDA). Mov Disord 24, 1779-1784.

Campuzano, V., Montermini, L., Molto, M.D., Pianese, L., Cossee, M., Cavalcanti, F., Monros, E., Rodius, F., Duclos, F., Monticelli, A., et al. (1996). Friedreich's ataxia: autosomal recessive disease caused by an intronic GAA triplet repeat expansion. Science 271, 1423-1427.

De Biase, I., Chutake, Y.K., Rindler, P.M., and Bidichandani, S.I. (2009). Epigenetic silencing in Friedreich ataxia is associated with depletion of CTCF (CCCTC-binding factor) and antisense transcription. PloS one 4, e7914.

Festenstein, R. (2006). Breaking the silence in Friedreich's ataxia. Nat Chem Biol 2, 512-513.

Festenstein, R., Sharghi-Namini, S., Fox, M., Roderick, K., Tolaini, M., Norton, T., Saveliev, A., Kioussis, D., and Singh, P. (1999). Heterochromatin protein 1 modifies mammalian PEV in a dose- and chromosomal-context-dependent manner. Nat Genet 23, 457-461.

Gale, E.A., Bingley, P.J., Emmett, C.L., and Collier, T. (2004). European Nicotinamide Diabetes Intervention Trial (ENDIT): a randomised controlled trial of intervention before the onset of type 1 diabetes. Lancet 363, 925-931.

Gottesfeld, J.M. (2007). Small molecules affecting transcription in Friedreich ataxia. Pharmacol Ther 116, 236-248.

Herman, D., Jenssen, K., Burnett, R., Soragni, E., Perlman, S.L., and Gottesfeld, J.M. (2006). Histone deacetylase inhibitors reverse gene silencing in Friedreich's ataxia. Nat Chem Biol 2, 551-558.

Hiragami-Hamada, K., Xie, S.Q., Saveliev, A., Uribe-Lewis, S., Pombo, A., and Festenstein, R. (2009). The molecular basis for stability of heterochromatin-mediated silencing in mammals. Epigenetics & chromatin 2, 14.

Horsman, M.R., Hoyer, M., Honess, D.J., Dennis, I.F., and Overgaard, J. (1993). Nicotinamide pharmacokinetics in humans and mice: a comparative assessment and the implications for radiotherapy. Radiother Oncol 27, 131-139.

Knip, M., Douek, I.F., Moore, W.P., Gillmor, H.A., McLean, A.E., Bingley, P.J., and Gale, E.A. (2000). Safety of high-dose nicotinamide: a review. Diabetologia 43, 1337-1345.

Koutnikova, H., Campuzano, V., Foury, F., Dolle, P., Cazzalini, O., and Koenig, M. (1997). Studies of human, mouse and yeast homologues indicate a mitochondrial function for FXN. Nat Genet 16, 345-351.

Puccio, H., and Koenig, M. (2002). Friedreich ataxia: a paradigm for mitochondrial diseases. Curr Opin Genet Dev 12, 272-277.

Rai, M., Soragni, E., Jenssen, K., Burnett, R., Herman, D., Coppola, G., Geschwind, D.H., Gottesfeld, J.M., and Pandolfo, M. (2008). HDAC inhibitors correct FXN deficiency in a Friedreich ataxia mouse model. PloS one 3, e1958.

Saveliev, A., Everett, C., Sharpe, T., Webster, Z., and Festenstein, R. (2003). DNA triplet repeats mediate heterochromatin-protein-1-sensitive variegated gene silencing. Nature 422, 909-913.

Stratford, M.R., Dennis, M.F., Hoskin, P.J., Saunders, M.I., Hodgkiss, R.J., and Rojas, A. (1996). Nicotinamide pharmacokinetics in normal volunteers and participants undergoing palliative radiotherapy. Acta oncologica (Stockholm, Sweden) 35, 213-219.

Stratford, M.R., Rojas, A., Hall, D.W., Dennis, M.F., Dische, S., Joiner, M.C., and Hodgkiss, R.J. (1992). Pharmacokinetics of nicotinamide and its effect on blood pressure, pulse and body temperature in normal human volunteers. Radiother Oncol 25, 37-42.

H. Akhlaghi, L.Corbenc, N.Georgiou-Karistianis, J.Bradshaw, M.B., Delatyckic, E.Storey, G.F.Egan, (2012) A functional MRI study of motor dysfunction in Friedreich's ataxia. Brain Research 1471 138-154

12. SUMMARY TIMELINES

12.1. Interventional Study (single and multiple dose, Parts I, II & III)

**Study Period:**	**Screening**	**Single Dose Escalation Part I**				
**Visit**	**≤ 28 days prior to visit 1**	**Visit 1 ≥ Day 1**	**Visit 2 ≥ Day 7**	**Visit 3 ≥ Day 14**	**Visit 4 ≥ Day 21**	**Visit 5 ≥ Day 28**
Telephone Pre-Screening	X					
Informed Consent	X					
Medical History and Physical Exam[6]	X					
Eligibility Assessment	X					
History of drug and alcohol use	X					
Body Mass Index (BMI)	X					
Height	X					
Weight	X					
Urinalysis [1]	X					
Haematology [2], Chemistry, Electrolytes and LFTs incl blood glucose measured by stick	X	X*	X*	X*	X*	X*
Pregnancy test [3]	X					
AE/ Con-meds	X	X	X	X	X	X
Vital Signs,[4]	X	X	X	X	X	X
12-lead ECG [5]	X					
Chromatin studies	X					X**
FXN measurements	X	X***	X***	X***	X***	X***
fMRI contraindications[7]	X					

* At pre-dose and 24 hours post-dosing
** At pre-dose and 8 hours post-dose.
*** At pre-dose, 2 (± 15 mins), 4 (± 25mins), 8 (± 1hr) and 24 (± 2.5hrs) hrs post-dose

Study Period:	Multiple Dose<br>Part II				
**Visit**	**Day1**<br>**≥ Day 35**	**Day2**<br>**≥Day 36**	**Day 3**<br>**≥Day 37**	**Day 4**<br>**≥Day 38**	**Day 5**<br>**≥Day 39**
Telephone Pre-Screening					
Informed Consent					
Medical History and Physical Exam					
Eligibility Assessment					
History of drug and alcohol use					
BMI					
Height					
Weight					
Urinalysis [1]					
Haematology [2], Chemistry, Electrolytes and LFTs incl BM measured by stick	X	X	X	X	X
Pregnancy test [3]					
AE/ Con-meds	X	X	X	X	X
Vital Signs,[4]	X	X	X	X	X
12-lead ECG [5]	X				X
FARS, SARA, SCAFI and speech dysarthria assessment .	X				X
Chromatin studies – pre-dose	X				X
FXN measurements*	X	X	X	X	X

* At pre-dose, 2 (± 15 mins), 4 (± 25mins), 8 (± 1hr) hrs post-dose. The pre-dose sample will also represent the 24 hour sample of the previous day. The last 24 hour sample will be taken on day 4. On day 5, the last 8hr PK sample may be taken (±2)hrs.

1. Urinalysis include: appearance, colour, pH, specific gravity, ketones, protein, glucose, bilirubin, nitrite, urobilinogen
2. Haematology include: white blood cell count with differential, red blood cell count, platelet count, haemoglobin, and haematocrit
3. Chemistry include: albumin, total bilirubin, urate, total cholesterol, creatinine, low-density and high-density lipoproteins, total protein, triglycerides, uric acid, Aspartate aminotransferase/glutamate oxalo-acetate transaminase (AST/GOT); alanine aminotransferase/glutamate pyruvate transaminase (ALT/GPT); γ-glutamyl transpeptidase (Gamma-GT); alkaline phosphatase (AP); lactate dehydrogenase (LDH); creatine kinase (CK)
4. Electrolytes include calcium, chloride, sodium, phosphorous, and potassium
   Blood glucose will be measured using a BM stick
5. Serum pregnancy test for women of childbearing potential only
6. Vital signs include pulse rate, blood pressure, respiration rate, pulse oximetry and temperature
7. ECG will be collected pre-dose on day 1 and prior to discharge on day 5
8. Participants who will enter Part III and not Part II of the study will also be assessed using the FARS, SCAFI and speech dysarthria assessment.
9. fMRI contraindications will be assessed as part of screening for participants who are due to take part in part IV of the study.

Study Period:	Multiple Dose<br>Part III		
**Weeks**	**Weeks 1, 3, 5 & 7 (7 ± 2 days)**	**Weeks 2, 4, 6, 8 (7 ± 2 days)**	**Interventional Study End of Study visit ****
Telephone Pre-Screening			
Informed Consent			
Medical History and Physical Exam			X
Eligibility Assessment			
History of drug and alcohol use			
BMI			X
Height			
Weight			X
Urinalysis [1]			X
Haematology [2], Chemistry, Electrolytes and LFTs incl BM measured by stick	X	X	X
Pregnancy test [3]			
AE/ Con-meds	X	X	X
Vital Signs[,4]	X	X	X
12-lead ECG [5]	X	X	X
FARS, SARA, SCAFI and speech dysarthria assessment		X	X
Chromatin studies		X*	X
FXN measurement pre-dose and 8hrs post-dose if necessary	X	X	X
Nicotinamide and anti-nausea tablets[6]	X	X	
Trial diary	X	X	

* Pre-dose chromatin samples to be taken monthly (at weeks 4 and 8).
** NB Participants who elect to only participate in either the single dose study (part I) or part II of the multiple dose but not both will have the final visit between 1 and 8 weeks after visit 5 of either the single dose or day 5 of the multiple dose part of the study.
If the participant elects to take part in all parts of the interventional study (Parts I, II and III) or only in Part II and Part III, participants will have the final visit between 1 and 8 weeks after day 5 of the multiple dose Part II of the study or visit 8 of the multiple dose Part III.

1. Urinalysis include: appearance, color, pH, specific gravity, ketones, protein, glucose, bilirubin, nitrite, urobilinogen
2. Haematology include: white blood cell count with differential, red blood cell count, platelet count, hemoglobin, and hematocrit
3. Chemistry include: albumin, total bilirubin, urate, total cholesterol, creatinine, low-density and high-density lipoproteins, total protein, triglycerides, uric acid, Aspartate aminotransferase/glutamate oxalo-acetate transaminase (AST/GOT); alanine aminotransferase/glutamate pyruvate transaminase (ALT/GPT); γ-glutamyl transpeptidase (Gamma-GT); alkaline phosphatase (AP); lactate dehydrogenase (LDH); creatine kinase (CK)

4. Electrolytes include calcium, chloride, sodium, phosphorous, and potassium. Blood glucose will be measured using a BM stick
5. Serum pregnancy test for women of childbearing potential only
6. Vital signs include pulse rate, BP, respiration rate, pulse oximetry and temperature
7. ECG will be performed predose weekly and at the EOS interventional visit. Prior to discharge on day 5 of Part II of the study, participants will be provided with nicotinamide supply for 7 ± 2 days and rescue medication (anti-nausea treatment, typically domperidone or metoclopromide). Domperidone will be taken prophylactically pre-dose (20 mg oral) and post-dose if required (20 mg orally up to four times daily.
8. Participants will also be given their first weekly diary card which will need to be completed each day for the entire duration of Part III. The diary will ensure participants will record whether or not they took nicotinamide, amount of tablets, any AEs and concomitant medications taken. Each week, the trial diary from the previous visit will be collected and participants will be provided with another diary to complete starting on the next day.

12.2. Non-Interventional Study (Part IV) for FRDA participants

Study Period:	Non-interventional study Part IV	
	Prior to study start	Study start, 2 weeks, 3 months and 9-12 months
Telephone call / email	X	
Informed Consent	X	
Eligibility Assessment i.e. fMRI contraindications	X	
Pregnancy test [1]		X
Edinburgh handedness questionnaire[2]		X
FXN protein estimation by LC-MS/MS and current dipstick assay (Mitosciences)		X
Analysis of the spatial organisation of the FXN locus by Chromosome-Conformation Capture coupled with high-throughput sequencing (3C-sequencing)		X
Biomarkers		X
SARA and SCAFI assessment		X
fMRI brain activation profile after a standardised finger-tapping task and object manipulation		X
Portable motion-capture device to be worn overnight		X
Standardised ADL tasks including SARA and SCAFI using full-body, motion-tracking ‘suit’		X
Eye-tracking (optional)		X
Balance control tasks (optional)		X

1. Urine pregnancy test for women of childbearing potential only
2. To be performed at study start only

12.3. Non-Interventional Study (Part IV) for healthy volunteers

Study Period:	Non-interventional study Part IV (may be collected over several visits)
Telephone call / email	X
Informed Consent	X
Eligibility Assessment i.e. fMRI contraindications	X
Pregnancy test [1]	X
Edinburgh handedness questionnaire	X
fMRI brain activation profile after a standardised finger-tapping task and object manipulation	X
Standardised ADL tasks including SARA and SCAFI using full-body, motion-tracking ‘suit’	X
Eye-tracking (optional)	X
Balance control tasks (optional)	X

[1] Urine pregnancy test for women of childbearing potential only

13. APPENDICES

13.1. Appendix 1: SARA scale

1) Gait		2) Stance	
Proband is asked (1) to walk at a safe distance parallel to a wall including a half-turn (turn around to face the opposite direction of gait) and (2) to walk in tandem (heels to toes) without support.<br><br>**0** **Normal, no difficulties in walking, turning and walking tandem (up to one misstep allowed)**<br>**1** **Slight difficulties, only visible when walking 10 consecutive steps in tandem**<br>**2** **Clearly abnormal, tandem walking >10 steps not possible**<br>**3** **Considerable staggering, difficulties in half-turn, but without support**<br>**4** **Marked staggering, intermittent support of the wall required**<br>**5** **Severe staggering, permanent support of one stick or light support by one arm required**<br>**6** **Walking > 10 m only with strong support (two special sticks or stroller or accompanying person)**<br>**7** **Walking < 10 m only with strong support (two special sticks or stroller or accompanying person)**<br>**8** **Unable to walk, even supported**		Proband is asked to stand (1) in natural position, (2) with feet together in parallel (big toes touching each other) and (3) in tandem (both feet on one line, no space between heel and toe). Proband does not wear shoes, eyes are open. For each condition, three trials are allowed. Best trial is rated.<br><br>**0** **Normal, able to stand in tandem for > 10 s**<br>**1** **Able to stand with feet together without sway, but not in tandem for > 10s**<br>**2** **Able to stand with feet together for > 10 s, but only with sway**<br>**3** **Able to stand for > 10 s without support in natural position, but not with feet together**<br>**4** **Able to stand for >10 s in natural position only with intermittent support**<br>**5** **Able to stand >10 s in natural position only with constant support of one arm**<br>**6** **Unable to stand for >10 s even with constant support of one arm**	
**Score**		**Score**	
**3) Sitting**		**4) Speech disturbance**	
Proband is asked to sit on an examination bed without support of feet, eyes open and arms outstretched to the front.<br><br>**0** **Normal, no difficulties sitting >10 sec**<br>**1** **Slight difficulties, intermittent sway**<br>**2** **Constant sway, but able to sit > 10 s without support**<br>**3** **Able to sit for > 10 s only with intermittent support**<br>**4** **Unable to sit for >10 s without continuous support**		Speech is assessed during normal conversation.<br><br>**0** **Normal**<br>**1** **Suggestion of speech disturbance**<br>**2** **Impaired speech, but easy to understand**<br>**3** **Occasional words difficult to understand**<br>**4** **Many words difficult to understand**<br>**5** **Only single words understandable**<br>**6** **Speech unintelligible / anarthria**	
**Score**		**Score**	

5) Finger chase

Rated separately for each side
Proband sits comfortably. If necessary, support of feet and trunk is allowed. Examiner sits in front of proband and performs 5 consecutive sudden and fast pointing movements in unpredictable directions in a frontal plane, at about 50 % of proband´s reach. Movements have an amplitude of 30 cm and a frequency of 1 movement every 2 s. Proband is asked to follow the movements with his index finger, as fast and precisely as possible. Average performance of last 3 movements is rated.

0 **No dysmetria**
1 **Dysmetria, under/ overshooting target <5 cm**
2 **Dysmetria, under/ overshooting target < 15 cm**
3 **Dysmetria, under/ overshooting target > 15 cm**
4 **Unable to perform 5 pointing movements**

Score	Right	Left
mean of both sides (R+L)/2		

6) Nose-finger test

Rated separately for each side
Proband sits comfortably. If necessary, support of feet and trunk is allowed. Proband is asked to point repeatedly with his index finger from his nose to examiner's finger which is in front of the proband at about 90 % of proband's reach. Movements are performed at moderate speed. Average performance of movements is rated according to the amplitude of the kinetic tremor.

0 **No tremor**
1 **Tremor with an amplitude < 2 cm**
2 **Tremor with an amplitude < 5 cm**
3 **Tremor with an amplitude > 5 cm**
4 **Unable to perform 5 pointing movements**

Score	Right	Left
mean of both sides (R+L)/2		

7) Fast alternating hand movements

Rated separately for each side
Proband sits comfortably. If necessary, support of feet and trunk is allowed. Proband is asked to perform 10 cycles of repetitive alternation of pro- and supinations of the hand on his/her thigh as fast and as precise as possible. Movement is demonstrated by examiner at a speed of approx. 10 cycles within 7 s. Exact times for movement execution have to be taken.

0 **Normal, no irregularities (performs <10s)**
1 **Slightly irregular (performs <10s)**
2 **Clearly irregular, single movements difficult to distinguish or relevant interruptions, but performs <10s**
3 **Very irregular, single movements difficult to distinguish or relevant interruptions, performs >10s**
4 **Unable to complete 10 cycles**

Score	Right	Left
mean of both sides (R+L)/2		

8) Heel-shin slide

Rated separately for each side
Proband lies on examination bed, without sight of his legs. Proband is asked to lift one leg, point with the heel to the opposite knee, slide down along the shin to the ankle, and lay the leg back on the examination bed. The task is performed 3 times. Slide-down movements should be performed within 1 s. If proband slides down without contact to shin in all three trials, rate 4.

0 **Normal**
1 **Slightly abnormal, contact to shin maintained**
2 **Clearly abnormal, goes off shin up to 3 times during 3 cycles**
3 **Severely abnormal, goes off shin 4 or more times during 3 cycles**
4 **Unable to perform the task**

Score	Right	Left
mean of both sides (R+L) / 2		

13.2. Appendix 2: SCAFI

Timed walking test: 8m walk (8MW)

☐ test not performed, reason:_______________________________________

☐ proband unable to walk due to physical limitations

assistive device: ☐ none ☐ one cane /crutches ☐ orthesis ☐ two cane /crutches ☐ wheeled walker

Did situations arise that necessitated repetition of a trial (e.g. proband fell, external interference during walking, examiner forgot to start/ reset stopwatch) ?
____

Other factors that might have affected performance ?
____

Times are only given for two successfully completed trials.

Trial 1 (0.1 sec) ☐ (8MW_T1)	Trial 2 (0.1 sec) ☐ (8MW_T2)

Timed dexterity test: 9-hole peg test (9HPT)

☐ test not performed, reason:_______________________________________

☐ proband unable to perform test due to physical limitations

Did situations arise that necessitated repetition of a trial (e.g. pegboard not sufficiently secured on the table, external interference, examiner forgot to start/ reset stopwatch/ turn pegboard)?_______________________________________

Other factors that might have affected performance ? ________________________

Times are only given for two successfully completed trials for each hand

**DOMINANT HAND**	**NON-DOMINANT HAND**
**Right** ☐<br>**Left** ☐<br><br>**Trial 1** ☐ **9HPTD_T1**<br>*(0.1 sec)*<br>**Trial 2** ☐ **9HPTD_T2**<br>*(0.1 sec)*	**Right** ☐<br>**Left** ☐<br><br>**Trial 1** ☐ **9HPTN_T1**<br>*(0.1 sec)*<br>**Trial 2** ☐ **9HPTN_T2**<br>*(0.1 sec)*

Timed speech task: PATA rate

☐ PATA rate task not performed, reason:________________________________

☐ unable to perform PATA rate task

Did situations arise that necessitated repetition of a trial (e.g. proband coughing, external interference during testing, examiner forgot to start stopwatch/ tape) ?

____

Other factors that might have affected performance ?_________________________

Counts are only given for two successfully completed trials.

Trial 1 ☐ PATA_T1	Trial 2 ☐ PATA_T2

SCA Functional Index.. ☐

13.3. Appendix 3: FARS SCALE PART II / Activities of Daily Living

ACTIVITIES OF DAILY LIVING (increments of 0.5 may be used if strongly felt that a task falls between 2 scores)

1.Speech...

- 0 - Normal
- 1 - Mildly affected. No difficulty being understood.
- 2 - Moderately affected. Sometimes asked to repeat statements.
- 3 - Severely affected. Frequently asked to repeat statements.
- 4 - Unintelligible most of the time.

2. Swallowing...

0 - Normal.
1 - Rare choking (< once a month).
2 - Frequent choking (< once a week, > once a month).
3 - Requires modified food or chokes multiple times a week. Or participant avoids certain foods.
4 - Requires NG tube or gastrostomy feedings.

3. Cutting Food and Handling Utensils..

0 - Normal.
1 - Somewhat slow and clumsy, but no help needed.
2 - Clumsy and slow, but can cut most foods with some help needed. Or needs assistance when in a hurry.
3 - Food must be cut by someone, but can still feed self slowly.
4- Needs to be fed.

4. Dressing...

0 - Normal.
1 - Somewhat slow, but no help needed.
2 -Occasional assistance with buttoning, getting arms in sleeves, etc. or has to modify activity in some way (e.g. Having to sit to get dressed; use velcro for shoes, stop wearing ties, etc.).
3 - Considerable help required, but can do some things alone.
4 -Helpless.

5. Personal Hygiene...

0 - Normal.
1 - Somewhat slow, but no help needed.
2 - Very slow hygienic care or has need for devices such as special grab bars, tub bench, shower chair, etc.
3 - Requires personal help with washing, brushing teeth, combing hair or using toilet.
4 - Fully dependent

6. Falling (assistive device = score 3)...

0 - Normal.
1 - Rare falling (< once a month).
2 - Occasional falls (once a week to once a month).
3 - Falls multiple times a week or requires device to prevent falls.
4 - Unable to stand or walk.

7. Walking (assistive device = score 3)..

0 - Normal.
1 - Mild difficulty, perception of imbalance.
2 - Moderate difficulty, but requires little or no assistance.

3 - Severe disturbance of walking, requires assistance or walking aids.
4 - Cannot walk at all even with assistance (wheelchair bound).

8. Quality of Sitting Position.. ☐
0 - Normal.
1 - Slight imbalance of the trunk, but needs no back support.
2 - Unable to sit without back support.
3 - Can sit only with extensive support (Geriatric chair, posy, etc.).
4 - Unable to sit.

9.Bladder Function (if using drugs for bladder, automatic score of 3)............. ☐
0 - Normal.
1 - Mild urinary hesitance, urgency or retention (< once a month).
2 - Moderate hesitance, urgency, rare retention/incontinence (> once a month, but < once a week).
3 - Frequent urinary incontinence (> once a week).
4 - Loss of bladder function requiring intermittent catheterization/indwelling catheter.

TOTAL ACTIVITIES OF DAILY LIVING SCORE: ☐

Imperial College London

13.4. Appendix 4: Edinburgh handedness score

Handedness Questionnaire

Instructions

For each of the activities below, please indicate:

Which hand you prefer for that activity?
Do you ever use the other hand for the activity?

Which hand do you prefer to use when:		no pref		Do you ever use the other hand?
Writing:	Left ○	○	○ Right	☐ Yes
Drawing:	Left ○	○	○ Right	☐ Yes
Throwing:	Left ○	○	○ Right	☐ Yes
Using Scissors:	Left ○	○	○ Right	☐ Yes
Using a Toothbrush:	Left ○	○	○ Right	☐ Yes
Using a Knife (without a fork):	Left ○	○	○ Right	☐ Yes
Using a Spoon:	Left ○	○	○ Right	☐ Yes
Using a broom (upper hand):	Left ○	○	○ Right	☐ Yes
Striking a Match:	Left ○	○	○ Right	☐ Yes
Opening a Box (holding the lid):	Left ○	○	○ Right	☐ Yes
items below are not on the standard inventory:				
Holding a Computer Mouse:	Left ○	○	○ Right	☐ Yes
Using a Key to Unlock a Door:	Left ○	○	○ Right	☐ Yes
Holding a Hammer:	Left ○	○	○ Right	☐ Yes
Holding a Brush or Comb:	Left ○	○	○ Right	☐ Yes
Holding a Cup while Drinking	Left ○	○	○ Right	☐ Yes

13.5. Appendix 5: Possible scenarios for the motion capture suit task

Interaction with objects to simulate normal every day activities.
We will investigate the unconstrained natural behaviour of a participant's daily activity, therefore the activities listed below are driven by what the participant would normally do and moreover any activity that they do not perform normally will be omitted. The protocol involves the following scenarios of activities of daily living:

Bedroom scenario: Participants are not tied to a specific order of doing things but are told that they will be expected to go through cycles of their normal evening and morning activities at home, including, but not limited to opening and closing the door, talking, reading, calling from a landline phone, putting on and removing clothes, lying down on the bed and packing their bag for work. This scenario will be set in a side room (with bed).

Office scenario: This scenario is the setup for observing how participants work in the office environment. It is approximately one hour long. Participants are provided with a desk, computer, paper and pen, a mouse and keyboard. No specific order of events is expected, but participants are given two tasks to complete but are allowed to work on any other tasks of their own. The scenario involves two tasks:
1. Reading a story printed over two double-sided A4 pages and underlining the first word of every sentence and
2. Using google to find a search query of 4 (or more) terms that produces a single search engine hit.

Breakfast scenario: This scenario is performed in a suitable area with a lunch table. During this recording, we will observe participants as they prepare their own breakfast and clear away the table when ready. Participants are free to have breakfast in any way they prefer. Breakfast components are provided for participants to be able to prepare and eat cereal, prepare and eat toast, drink from a cup, eat an apple and clear the table. Participants are asked to eat one slice of toast with a fork and knife, if possible. For issues of health and safety, they are not allowed to use running water whilst wearing the suit.

Repeated identical daily standard tasks:
Participants will be asked to repeatedly perform tasks from a set of everyday tasks. All trials are from a defined starting posture [(similar to a soldier standing at attention, if possible)]. Each activity will be repeated a number of times (typically 10, but a lower number can be adapted to suit the participants capabilities). Participants will be able to rest at any time during the experiment after informing the experimenter. After every 30 trials a mandatory short resting phase (minimum of 2 mins or as long as the participant desires) is scheduled, depending on how much effort the task requires in order to minimise fatigue in participants. The tasks are as follows: sitting down and standing up (if applicable), lying down and getting up, rolling in bed, pouring water into a cup, using knife and fork to cut toast in half, picking up a tennis ball from the floor, grasping a tennis ball, combing hair at back of head, brushing teeth, pronation and supination, elbow flexion and extension, walking from one room to another (if applicable), handling and opening and closing a door.

13.6. **Appendix 6: Balance Control Tasks (optional)**

The Balance Control tasks involve the use of a Force Plate (Nintendo Wii Balance Board) and a visual display unit. The tasks are shown in the computer screen and oral guidance on how to perform these tasks will be given to the participant before the start of the experiment.

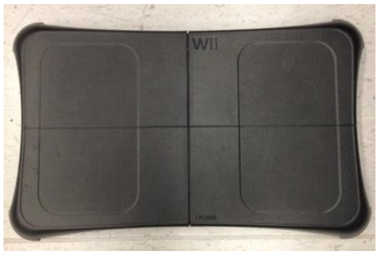

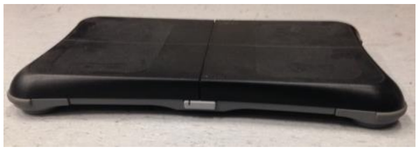

The participants will be asked to stand on the force plate and perform two tasks, directed by the computer. The risks of standing on the force plate are no more than the risks of standing on an elevated plane above the ground. To minimize the risk of falling, the researcher will be next to the participant, should support be needed at any point in time.

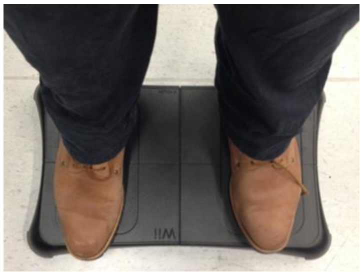

The experiment will be initialised by the researcher, and visual cues will be given on the visual display to the participant for the different tasks.
