# Supplementary material for: A wearable motion capture suit and machine learning predict disease progression in Friedreich’s ataxia
Source: Nat Med. 2023 Jan 19;29(1):86–94. doi: 10.1038/s41591-022-02159-6 (PMC9873563; doi:10.1038/s41591-022-02159-6)
Supplement: Supplementary file 2 — Reporting Summary [file 41591_2022_2159_MOESM2_ESM.pdf]

nature portfolio

Corresponding author(s): A.Aldo Faisal
Last updated by author(s): Oct 25, 2022

Reporting Summary

Nature Portfolio wishes to improve the reproducibility of the work that we publish. This form provides structure for consistency and transparency in reporting. For further information on Nature Portfolio policies, see our Editorial Policies and the Editorial Policy Checklist.

Statistics

For all statistical analyses, confirm that the following items are present in the figure legend, table legend, main text, or Methods section.

n/a	Confirmed	
☐	☒	The exact sample size (*n*) for each experimental group/condition, given as a discrete number and unit of measurement
☐	☒	A statement on whether measurements were taken from distinct samples or whether the same sample was measured repeatedly
☐	☒	The statistical test(s) used AND whether they are one- or two-sided<br>*Only common tests should be described solely by name; describe more complex techniques in the Methods section.*
☐	☒	A description of all covariates tested
☐	☒	A description of any assumptions or corrections, such as tests of normality and adjustment for multiple comparisons
☐	☒	A full description of the statistical parameters including central tendency (e.g. means) or other basic estimates (e.g. regression coefficient) AND variation (e.g. standard deviation) or associated estimates of uncertainty (e.g. confidence intervals)
☐	☒	For null hypothesis testing, the test statistic (e.g. *F*, *t*, *r*) with confidence intervals, effect sizes, degrees of freedom and *P* value noted<br>*Give P values as exact values whenever suitable.*
☒	☐	For Bayesian analysis, information on the choice of priors and Markov chain Monte Carlo settings
☒	☐	For hierarchical and complex designs, identification of the appropriate level for tests and full reporting of outcomes
☐	☒	Estimates of effect sizes (e.g. Cohen's *d*, Pearson's *r*), indicating how they were calculated

Our web collection on statistics for biologists contains articles on many of the points above.

Software and code

Policy information about availability of computer code

Data collection	IGS-180 motion capturing suit software, purpose developed code in MATLAB (R2015b) and MATLAB (R2019b)
Data analysis	Purpose developed code in MATLAB (R2015b) and MATLAB (R2019b). The machine learning code that supports the findings of this study is available for academic purposes at https://doi.org/10.6084/m9.figshare.20440449

For manuscripts utilizing custom algorithms or software that are central to the research but not yet described in published literature, software must be made available to editors and reviewers. We strongly encourage code deposition in a community repository (e.g. GitHub). See the Nature Portfolio guidelines for submitting code & software for further information.

Data

Policy information about availability of data

All manuscripts must include a data availability statement. This statement should provide the following information, where applicable:

- Accession codes, unique identifiers, or web links for publicly available datasets
- A description of any restrictions on data availability
- For clinical datasets or third party data, please ensure that the statement adheres to our policy

The data used in the study are not publicly available due to them containing information that could compromise research participant privacy/consent. Anonymized data can be made available for academic purposes upon request to the corresponding author.

Human research participants

Policy information about studies involving human research participants and Sex and Gender in Research.

Reporting on sex and gender	We recruited nine patients (3 males and 6 females, aged 24-63) and nine age- and gender-matched controls for the study. Gender was included in the study design. Both male and female being eligible for inclusion. The gender was self-reported and recorded together with other demographic features. Given the small number of males, no sex disaggregated analysis was statistically feasible in this exploratory study.
Population characteristics	Baseline variables presented in Table 1.
Recruitment	Potentially eligible participants were recruited verbally in outpatient clinics at the National Hospital for Neurology and Neurosurgery,UCLH (London, UK) and the Imperial College Healthcare NHS Trust (London, UK). Patients who fulfilled the inclusion criteria were given the choice to be included in the study and most patients agreed minimising self selection bias.
Ethics oversight	Our clinical trial was approved by the UK Medicines and Healthcare Products Regulatory Agency (MHRA; EudraCT 2011-002744-27), the Riverside Research Ethics Committee (11/LO/0998) and the Imperial College London Joint Research & Compliance Office (Please see supplementary note for the study protocol).

Note that full information on the approval of the study protocol must also be provided in the manuscript.

Field-specific reporting

Please select the one below that is the best fit for your research. If you are not sure, read the appropriate sections before making your selection.

☒ Life sciences ☐ Behavioural & social sciences ☐ Ecological, evolutionary & environmental sciences

For a reference copy of the document with all sections, see nature.com/documents/nr-reporting-summary-flat.pdf

Life sciences study design

All studies must disclose on these points even when the disclosure is negative.

Sample size	A formal sample size calculation was not appropriate for this exploratory study. A statistical rule of thumb was applied to this adaptive study design as no formal power calculations could be performed. We therefore recruited nine patients (3 males and 6 females, aged 24-63) and nine age- and gender-matched controls which was sufficient to develop the methodology outlined in the paper.
Data exclusions	One of the FA patients dropped out of the study after 2nd visit due to personal reasons however, we still included the collected data in the further analysis where possible. Motion capture suit data was not collected during the last visit of another patient because of technical issues. Blood sample for FXN measurement was not collected for a patient.
Replication	A stricter leave one subject out cross-validation policy (instead of a leave-one-visit-out) was used to ensure the generalization of the results.
Randomization	As this was a natural history study and not an interventional trial, randomization is not applicable. It is in the nature of the wearables study to collect behaviour from subjects who were aware that they wore sensors.
Blinding	As this was a natural history study and not an interventional trial, blinding is not applicable. It is in the nature of the wearables study to collect behaviour from subjects who were aware that they wore sensors.

Reporting for specific materials, systems and methods

We require information from authors about some types of materials, experimental systems and methods used in many studies. Here, indicate whether each material, system or method listed is relevant to your study. If you are not sure if a list item applies to your research, read the appropriate section before selecting a response.

Materials & experimental systems

n/a	Involved in the study
☒	☐ Antibodies
☒	☐ Eukaryotic cell lines
☒	☐ Palaeontology and archaeology
☒	☐ Animals and other organisms
☐	☒ Clinical data
☒	☐ Dual use research of concern

Methods

n/a	Involved in the study
☒	☐ ChIP-seq
☒	☐ Flow cytometry
☒	☐ MRI-based neuroimaging

Clinical data

Policy information about clinical studies
All manuscripts should comply with the ICMJE guidelines for publication of clinical research and a completed CONSORT checklist must be included with all submissions.

Clinical trial registration
EudraCT 2011-002744-27

Study protocol
Available in the supplementary note section of the supplementary materials

Data collection
Data collection happened between 26th November, 2013 and 5th June, 2015 at Imperial College Clinical Research Facility. Measurements were taken four times from the FA patients and once from the controls during the course of the study (Day-1/ Baseline, after 3 weeks, 3 months and 9 months)

Outcomes
Given the novel methodology, it was an exploratory non-interventional study and so did not have predefined outcome measures apart from the clinical scales used which had been previously validated. This was a comparison between clinical scales (SARA and SCAFI) and kinematic bio-markers developed using machine learning algorithms during the course of the study. The study was to determine a good outcome measure for future clinical trials by comparison with existing validated clinical outcome measures. We embarked on this study following a clinical trial (EudraCT 2011-002744-27) for Nicotinamide (Libri et al, Lancet, 2014) as there was clearly a need for better outcome measures in this disease. Approval was granted by the ethics committee to proceed with this study as an amendment to the clinical trial protocol. The study presented here is therefore not a phase II or III clinical trial and as such does not require CONSORT checklist as per guidelines.
